# Shadow-Induced Forgetting in a Game-Based Paradigm on Nonclinical Adults and Its Effects on Consciousness, Emotional Valence, and Temporal Dynamics: Crossover Study

**DOI:** 10.2196/76946

**Published:** 2025-12-30

**Authors:** Yoon Jin Cho, Ka Hyun Kim, Da Won Suh, In Seong Baek, Jung Hun Phee, Jae Yong Yu, Kyung Min Kim, Yu Rang Park

**Affiliations:** 1 College of Medicine, Yonsei University, Seoul, Republic of Korea; 2 Department of Applied Statistics, College of Commerce and Economics, Yonsei University, Seoul, Republic of Korea; 3 Department of Biotechnology, College of Life Science and Biotechnology, Yonsei University, Seoul, Republic of Korea; 4 Department of Statistics and Data Science, College of Commerce and Economics, Yonsei University, Seoul, Republic of Korea; 5 Healthcare AI Assurance Lab, GFRC, Hallym University, Chuncheon-si, Gangwon-do, Republic of Korea; 6 Research Institute for Data Science and AI (Artificial Intelligence), Hallym University, Chuncheon-si, Gangwon-do, Republic of Korea; 7 Division of Data Science, Hallym University, Chuncheon-si, Gangwon-do, Republic of Korea; 8 Department of Emergency Medicine, Hallym University, Chuncheon-si, Gangwon-do, Republic of Korea; 9 Department of Neurology, College of Medicine, Yonsei University, Seoul, Republic of Korea; 10 Department of Biomedical Systems Informatics, College of Medicine, Yonsei University, 50-1, Yonsei-Ro, Seodaemun-gu, Seoul, 03722, Republic of Korea, 82 2-2228-2493

**Keywords:** shadow-induced forgetting, game-based treatment, memory suppression, electroencephalography, hippocampus

## Abstract

**Background:**

Memory suppression transiently disrupts hippocampal activity, leading to suppression-induced forgetting, especially for negative stimuli. However, traditional paradigms such as Think/No-Think rely on explicit control and lack ecological validity. This study introduces a game-based task that implicitly elicits suppression through reversed motor mappings, providing a naturalistic approach to studying memory inhibition.

**Objective:**

This study aims to examine how shadow-induced forgetting (ShIF) varies across short-term and long-term intervals (0 hours, 24 hours, and 72 hours), under conscious versus unconscious exposure, and between positive and negative emotional stimuli.

**Methods:**

This single-center, within-subjects experimental study involved 56 university students (mean age 23.37, SD 1.84 years) and was conducted between December 1, 2023, and March 1, 2024. Participants learned 36 cue-target image pairs varying in emotional valence (positive and negative). They underwent a game-based paradigm where habitual motor responses were disrupted through reversed key mappings to induce an amnesic shadow. During gameplay, selected cues were presented either consciously or unconsciously, while others served as controls. Memory performance was assessed using Metric for Evaluation of Translation with Explicit Ordering scores (semantic similarity) immediately after intervention (0 hour) and again at 24 hours and 72 hours. Electroencephalography was recorded in a subset of 40 participants to examine neural correlates of memory suppression.

**Results:**

ShIF effects were short-term, conscious-dependent, and selective for negative memories. A significant interaction between exposure condition and time (*F*_₃.₆₂,₁₉₉.₃₃_=2.7, *P*=.04, *η*²_*p*_=0.05, 95% CI 0.00-1.00) indicated that the effect varied across time points. Specifically, a significant ShIF effect emerged immediately after the intervention (0 hour) in the conscious condition (*t*_₅₅_=−2.86, *P*=.02, *d*=0.38) but was absent in the unconscious condition and dissipated by 72 hours. Robust main effects of time (*F*_₂,₁₀₉.₉₉_=102.91, *P*<.001, *η*²_*p*_=0.65, 95% CI 0.57-1.00) and emotional valence (*F*_₁,₅₅_=42.43, *P*<.001, *η*²_*p*_=0.44, 95% CI 0.27-1.00) showed that overall recall declined over time and was consistently lower for negative images. Electroencephalography analyses revealed enhanced right frontal beta (FC6, F4, and F8: *P*<.001) and posterior gamma (O1: *P*<.001, O2: *P*<.001, and P8: *P*=.002) activity during suppression of negative cues, reflecting neural inhibition processes underlying ShIF.

**Conclusions:**

ShIF occurs primarily for consciously processed negative memories and diminishes over several days, highlighting the temporal and emotional boundaries of intentional forgetting. This study introduces a game-based approach that extends traditional suppression paradigms and offers an ecologically valid framework for investigating memory control. Importantly, we demonstrate that suppression can be induced through a game-based paradigm. By examining emotional valence, exposure condition, and temporal dynamics, we extend previous work focused only on transient effects and clarify the potential for practical implementation in digital therapeutic applications such as posttraumatic stress disorder treatment.

## Introduction

Memory comprises multidimensional components that can be accessed through specific cues. In daily life, various stimuli prompt memory retrieval; however, when faced with cues linked to negative experiences such as violence, loss, or death, individuals often attempt to consciously suppress these memories [1]. Such suppression can transiently disrupt hippocampal activity, producing effects similar to organic amnesia, including both retrograde and anterograde memory impairments [2-5]—a process known as suppression-induced forgetting [6]. Importantly, these forgetting effects are shown to be particularly pronounced for emotionally negative stimuli [7-10]. This is because negative emotion tends to elicit stronger reactivation and higher arousal [11,12], which narrows the focus of attention to central details while reducing peripheral processing [13], potentially facilitating the forgetting of contextually associated information.

The Think/No-Think (TNT) paradigm has been the most widely used method for experimentally inducing intentional forgetting [14]. In this paradigm, participants are instructed either to recall (Think) or to suppress (No-Think) specific memory associations when presented with trained cue-target pairs. However, the TNT paradigm’s reliance on explicit retrieval cues and participants’ voluntary compliance introduces substantial variability across individuals. Because outcomes depend on one’s metacognitive ability to intentionally suppress retrieval, this paradigm has limited ecological validity and generalizability to everyday memory processes [15]. Revisiting Anderson and Green’s foundational discussion [1], it has been proposed that the neural systems responsible for memory suppression overlap substantially with those engaged during motor inhibition. This theoretical overlap suggests that inhibitory control in memory could also be recruited indirectly through tasks that suppress motor responses.

Building on this perspective, this study introduces a game-based approach that embeds memory suppression within a naturalistic, rule-based environment. During a reverse phase (RP) in the game, participants experienced reversed mappings between arrow keys and character movements while previously learned scene images appeared on the screen. These reversed mappings functioned as a behavioral analogue to the No-Think phase in the TNT paradigm, requiring participants to override prepotent motor responses. By doing so, our paradigm naturally invoked inhibitory control without explicit instructions to suppress recall, thereby providing an implicit and ecologically valid means of eliciting suppression-like effects similar to those observed in traditional suppression-induced forgetting paradigms.

The suppression creates a temporal disruption during which hippocampal processing is actively inhibited, termed an amnesic shadow [16], lasting approximately 5-10 seconds before and after each suppression attempt. This temporal disruption is consistent with earlier findings by Fawcett and Taylor [17], who reported that intentional forgetting can transiently impair the encoding of subsequently presented information, likely due to the temporary engagement of working memory control processes. During this period, newly encoded or reactivated memories may weaken, allowing specific memory traces to be intentionally attenuated through targeted suppression. Related studies have investigated memory weakening through reactivation of unrelated episodic memories during the amnesic shadow, a phenomenon referred to as shadow-induced forgetting (ShIF) [5]. Unlike conventional TNT-based suppression, ShIF occurs indirectly within the amnesic shadow generated by unrelated memories rather than through explicit suppression of target retrieval, and it can also emerge when unrelated memories appear unconsciously [18].

However, empirical evidence confirming memory weakening through the amnesic shadow remains limited. Previous studies [5,18,19] have primarily examined ShIF using negative images, lacking diversity in emotional valence and failing to assess its temporal persistence. Moreover, quantitative analyses of ShIF have seldom addressed its short-term versus long-term dynamics. Therefore, the primary objectives of this study were to investigate how the ShIF effect differs (1) across short-term (0 hour) and long-term (24 hours and 72 hours) contexts, (2) under varying image exposure conditions (conscious vs unconscious), and (3) according to image valence (positive vs negative). We hypothesize that the effect will (1) remain significant in both short- and long-term contexts, (2) be significant in both conscious and unconscious exposure conditions, and (3) be more significant under negative images.

In addition, by incorporating electroencephalography (EEG) and continuous behavioral tracking, we extended prior research on amnesic shadow–related forgetting to a neural level. EEG-based measurements were used to quantify ShIF effects across time, providing an objective index of suppression-related dynamics.

By elucidating the characteristics of ShIF under diverse conditions and presenting quantitative evidence from both short- and long-term perspectives, our study advances the current understanding of this phenomenon and contributes to its potential future application in mitigating distress associated with intrusive memories commonly experienced in posttraumatic stress and obsessive-compulsive disorders.

## Methods

### Overview of Basic Methodology

**Figure 1.**
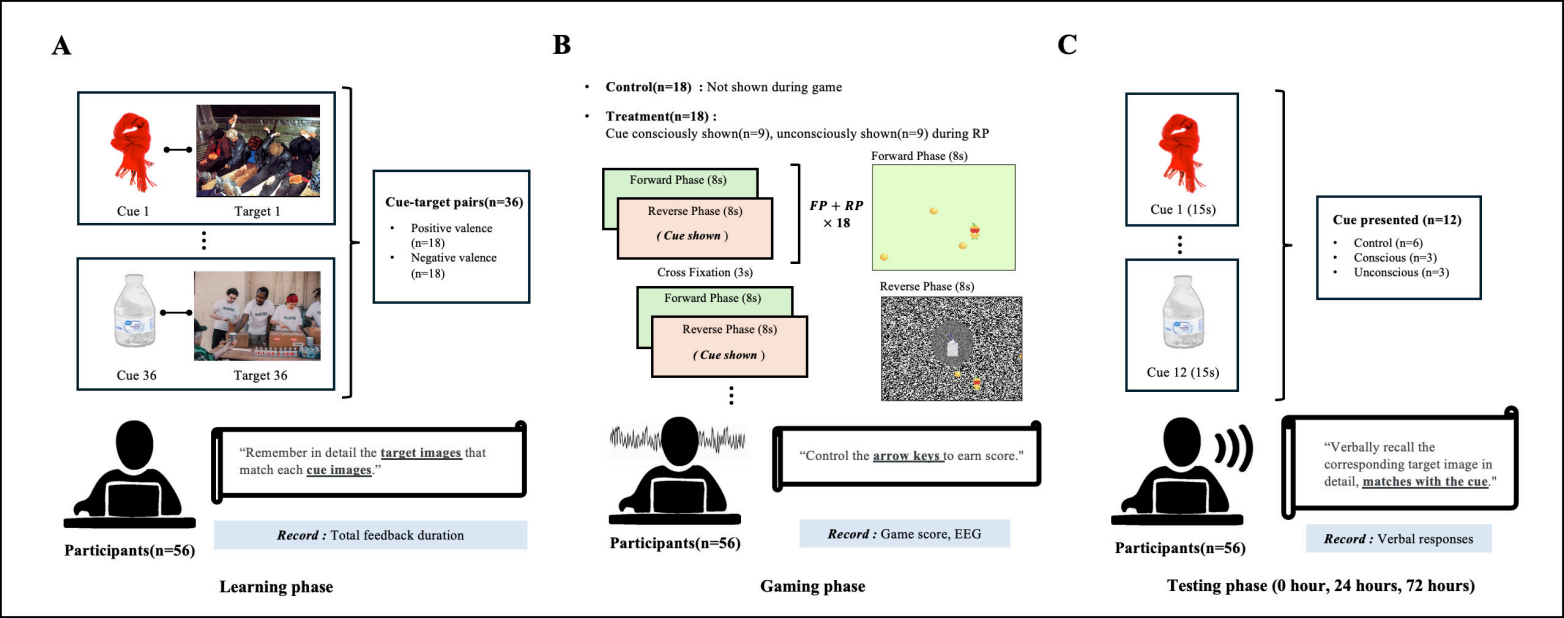
Overall procedure and flowchart of the experiment. (**A**) In the Learning phase, the researcher presented 36 cue-target pairs (18 positive and 18 negative images) to the participants in sequence, instructing them to concentrate on the cue and memorize the target (step 1). Subsequently, participants fully acquired the cue-target pairs via a self-feedback mechanism (step 2). (**B**) In the Gaming phase, participants engaged in a game intended to induce memory weakening. The prelearned cues in (**A**) were displayed in the background during the game. Among the 36 cue-target pairings, 18 pairs belonged to the control group and were not presented during the game. The remainder belonged to the treatment group, with 9 pairs presented as stimuli that participants could perceive (conscious), while the other 9 pairs were masked stimuli that participants could not perceive (unconscious). EEG measurements were recorded throughout the game. (**C**) The Testing phase was designed to assess whether the cue-target pairing acquired before the game had diminished in strength. The assessment was conducted 3 times: shortly after the Gaming phase, 24 hours later, and 72 hours later. To assess the degree of forgetting, participants were randomly presented with 6 cues from the control group, 3 cues from treatment group 1 (conscious), and 3 cues from treatment group 2 (unconscious) during each testing phase, drawn from a total of 36 prelearned cue-target pairs. The descriptions of the target images provided by each participant were subsequently evaluated based on their Metric for Evaluation of Translation with Explicit Ordering score. EEG: electroencephalography; FP: forward phase; RP: reverse phase.

A within-subjects, prospective design was used to assess memory performance based on 3 within-subject variables: exposure condition (consciously cued, unconsciously cued, and control), emotional valence (positive and negative), and test time point (0 hour, 24 hours, and 72 hours). In this design, the participants acted as their own controls and were exposed to all levels of these variables. The study was conducted in 3 phases: Learning, Gaming, and Testing. In the Learning phase, participants learned 36 cue-target pairs using a self-feedback process. The target images had either positive or negative emotional valence. In the Gaming phase, participants engaged in a game designed to induce amnesic shadows, in which 18 of the previously learned cues were exposed to the participants either in a conscious or unconscious manner. Participants were tested on the previously learned cue-target pairs at three time points: (1) immediately after, (2) 24 hours after, and (3) 72 hours after the Gaming phase. The experimental procedure is outlined in Figure 1.

We followed the TREND (Transparent Reporting of Evaluations with Nonrandomized Designs) statement [20] to ensure comprehensive and transparent reporting of this within-subject experimental study. A completed TREND checklist is provided.

### Participants

Recruitment of participants was done through convenience sampling via surveys targeting students at Yonsei University, Seoul, South Korea, from December 1, 2023, to March 1, 2024, which complied with ethical regulations for research on human participants. The surveys were posted on Yonsei University’s bulletin boards and online community.

Then, the final participants were selected according to the inclusion criteria: (1) undergraduate or graduate students aged 19‐29 years enrolled in Yonsei University, (2) corrected vision of 0.5 or better in both eyes, (3) no known health issues, and (4) Korean language proficiency. The study analyzed a final sample of 56 participants (Figure S1 in Multimedia Appendix 1). Initially, 85 potential participants completed an online prescreening survey. During the online prescreening survey, participants were asked whether they were currently undergoing any form of medical or psychological treatment, and none reported having an active condition. Nineteen participants were excluded on the basis of predetermined criteria (age, language, and vision). The remaining 66 individuals were enrolled after providing written informed consent. For the analysis, 10 participants were excluded because of technical issues, allocation errors, voluntary withdrawals, and incomplete data.

The final 56 participants had a mean age of 23.37 (SD 1.84) years, and 62.5% (35/56) of the participants were female. Their mean corrected vision was 0.99 (SD 0.21) (Table S1 in Multimedia Appendix 1). All participants met the inclusion criteria (aged 19‐29 years, fluent in Korean, currently enrolled in university, and having a corrected vision of 0.5 or better).

### Materials

#### Target Images

A total of 36 target images were used for learning. The selection criteria for these images were based on the presence of appropriate gists inside each image and the clarity of the plot. The images were sourced from the International Affective Picture System [21] and several online commercial platforms, such as iStock Photo, with reference to previous studies [22,23]. Among the 36 images, 18 had a positive emotional valence (eg, babies and families, romance, adventure, or sports [23]) and the other 18 were negative (eg, physical and sexual assault, witnessing injuries and death, natural disasters, and serious accidents [5]). The emotional valence associated with each image was established through consensus among 5 experimenters and an independent judgment group of 12 individuals.

#### Cues

Each target image was paired with a cue chosen to simulate natural involuntary recall of the target image. Each cue was selected from the object items embedded in the respective target image [9] and had to be irrelevant to the gist of the target image. The cues were determined by consensus among the 5 experimenters and an independent judgment group of 12 individuals.

#### Target Descriptions

To compute the Metric for Evaluation of Translation with Explicit Ordering (METEOR) score and BERTScore, descriptions for each target image were extracted through the following process. First, textual descriptions of each target image were collected from 12 subjects who participated in the pilot study. Compensation was provided to participants to regulate the quality of their descriptions. For each target image, a unified description was extracted by prompting GPT 4.0, with all descriptions gathered from the 12 participants. Descriptions were standardized to a length of approximately 300 characters using a large language model. Five experimenters achieved agreement on the final target descriptions.

#### Gamification of the TNT Paradigm

In the game, participants controlled the character’s movements using the arrow keys and earned points by successfully dodging balls launched from various directions. The game was developed using JavaScript on the HTML (Hypertext Markup Language) template (see the “Data Availability” section for details).

Each game set comprised 2 phases: a forward phase (FP) lasting 8 seconds and an RP lasting 8 seconds, resulting in a total duration of 16 seconds (FP + RP) per set.

In the FP, the 4 arrow keys (up/down and right/left) corresponded to the movement direction of the character. In the RP, either the up/down or right/left arrow keys were reversed, causing the character to move in the direction opposite to what was intended. The reversal pattern was randomized for each set.

During the RP, a cue was presented on the screen in either a conscious or unconscious manner. The unconscious manner was achieved through repeated presentation of the cue for 16.7 milliseconds, masked before and after by a 183.3-millisecond white Gaussian noise mask (Figure S2 in Multimedia Appendix 1).

The undetectability of the unconsciously presented cues was assessed in a separate group of 47 participants. Upon completion of the Learning and Gaming phases, the participants were asked the following question: “During the reverse phase of the game, when the cue was not presented consciously, what did you perceive?” No specific object was reported by any participant. Typical responses included statements such as “a flickering screen” or “something passed by, but I could not tell what it was.”

#### Randomization

The sequence of image presentation during the game and test was established through controlled randomization for all participants. The exposure condition (consciously cued, unconsciously cued, and control) of the 36 cue-target pairs underwent controlled randomization to guarantee an even distribution among the subjects.

### Procedure

#### Setting

The entirety of the procedure was conducted at Yonsei University Campus Town S-Cube No. 2 in Seodaemun-gu, Seoul. The experiment and, simultaneously, data collection began in November 2023 and ended on March 3, 2024.

#### Learning Phase

In the Learning phase, the participants learned 36 pairs of cue-target images. Each pair was displayed consecutively on the screen for 3 seconds, during which participants were instructed to memorize the details of the target image and associate it with the paired cue. Afterward, the participants went through a self-feedback process; this time, the cue was presented on the screen independently, and participants were instructed to press a “yes” or “no” button depending on whether they could recall the paired target image well enough. Upon pressing either of the 2 buttons, or after 3 seconds of no response, the subsequent cue was displayed on the screen. During the self-feedback process, cues that participants were unable to recall were represented. The Learning phase ended when the participant determined that all cue-target image pairs had been learned. The experimental manipulation was performed using PsychoPy [24].

#### Gaming Phase

The game was played on a laptop. Each participant played 19 game sets, with the initial set designated for practice. A fixation cross was displayed for 3 seconds between each set. Of the 36 previously learned cues, 18 (associated with 9 positive and 9 negative targets) were randomly selected and presented during the game (treatment), whereas the remaining 18 were not (control). Of the 18 cues, 9 were presented in a conscious manner (conscious), while the other 9 were presented in an unconscious manner (unconscious). The conscious and unconscious cues were presented alternately. The allocation of the exposure conditions (conscious, unconscious, and control) of the 36 cues was randomized for each participant. EEGs were conducted during the Gaming phase.

#### Testing Phase

In the Testing phase, each cue was displayed consecutively on the screen for 15 seconds, during which the participants were instructed to verbally describe the corresponding target image, incorporating as many elements as possible. Responses were recorded using a smartphone device with the participants’ prior consent. Each participant underwent 3 tests, with 12 cue-target image pairs assessed in each test: (1) immediately after, (2) 24 hours after, and (3) 72 hours after the Gaming phase. Across the 3 sessions, all 36 cue-target pairs were tested once without repetition, thereby covering the entire set of learned associations. Participants were not informed about the presence of subliminally presented cues to preserve the purity of the implicit learning effects and to avoid metacognitive contamination [25] or explicit memory encoding [26], which could induce reflective monitoring or expectancy biases and thereby interfere with unconscious processing. Experimental manipulation was carried out using PsychoPy [24].

### Data and Assessment

From the experiment, participants’ game scores, total feedback duration, EEG data, and verbal responses were collected and processed, as seen in Figure 1. Primarily, verbal responses were processed into the outcome variables: METEOR score, the Gist score, and the BERTScore. EEG data were also collected as the outcome variable. The exposures consisted of 3 within-subject factors: exposure condition (consciously cued, unconsciously cued, or control), emotional valence (positive or negative), and test time point (0 hour, 24 hours, or 72 hours). Two predictors were included as covariates to control for individual differences in engagement and learning effort: participants’ game score and total feedback duration. Several potential confounders were also identified, including stress induced by flickering stimuli, unassessed clinical or trauma history, variability in concentration and gaming experience, a limited number of items per cell in the 3×3×2 design, the absence of a neutral baseline condition, and potential assessment imprecision due to the small independent norming sample.

#### Verbal Responses

The participants’ verbal recall responses collected during the Testing phase were the primary source of data for all outcome variables. All responses (n=2016, 56 participants × 36 responses from each) were audio-recorded, transcribed verbatim in Korean using Clova Note, and then translated into English using DeepL. Then, the scores were calculated using the following 2 evaluation scales. To adjust for variances between participants, the scores for all responses were standardized—mean 0 (SD 1)—for each participant. Considering the small sample size, the median was selected as the representative value.

To quantitatively assess participants’ recall performance, we designated the METEOR score as the primary outcome variable, supplemented by 2 additional outcome variables —Gist score [27] and BERTScore [28]—for validation purposes. The additional metrics were included not as alternative outcomes but to examine whether METEOR produced results consistent in overall pattern while providing a more detailed and objective measure of recall accuracy. This cross-metric validation was conducted to establish the construct validity and measurement reliability of the METEOR approach. Details for each metric are as follows.

#### Cross-Metric Validation

To evaluate the validity of using METEOR as the primary measure of recall performance, we examined its correlations with both the human-rated Gist scores and the embedding-based BERTScore across all responses (n=2016). As summarized in Table S2 in Multimedia Appendix 1*,* METEOR demonstrated strong positive correlations with both Gist (*r*=0.773, 95% CI 0.755-0.790; *P*<.001) and BERTScore (*r*=0.772, 95% CI 0.753-0.789; *P*<.001). These findings indicate that METEOR captures variance consistent with both human judgments and semantic embedding similarity, supporting its construct validity as an automated and linguistically grounded measure of recall accuracy.

Importantly, GPT-4 was used solely to standardize the linguistic structure of target descriptions, not to generate or evaluate recall scores, ensuring that the scoring process remained objective and bias-free.

#### EEG Data

Among the 56 participants, the EEG data from 16 were excluded because of poor signals. The portable EEG device EpocX (Emotiv, Inc) was used for data collection. Data were recorded from the frontal (AF3, AF4, F7, F3, F4, F8, FC5, and FC6), temporal (T7 and T8), parietal (P7 and P8), and occipital (O1 and O2) areas according to the international 10‐20 system. The sampling frequency was 128 per second.

To remove the effects of line noise above 50‐60 Hz and cathode ray tube noise, the data were filtered using a lower passband edge of 0.1 Hz and a higher passband edge of 45 Hz [29]. A finite impulse response filter was used, as described previously [30]. Accordingly, MNE-Python’s (MNE Project) finite impulse response filter was applied with a bandpass setting between 0.1 Hz and 45 Hz [31,32]. The average was used as the EEG reference [33]. For analysis, the power spectrum distribution of each epoch was calculated for the delta (0.5‐4 Hz), theta (4‐8 Hz), alpha (8‐13 Hz), beta (13‐30 Hz), and gamma (30‐100 Hz) frequency bands using the Welch method with a Hanning taper [34,35].

### Statistical Analysis

The outcomes of the 56 participants who completed the entire experiment were analyzed. Statistical analyses were performed using R (version 4.4.0; R Core Team). All data are available in the Open Science Framework (see the “Data Availability” section). The level of statistical significance was set at α=.05, and the CI at 95%.

#### Simulation-Based Post Hoc Power Analysis

To address potential concerns regarding the small number of cue-target pairs per condition (approximately 2‐3 items per cell, 36 pairs across 18 cells) and the sample size (n=56), a simulation-based post hoc power analysis was conducted using the Superpower package in R (Comprehensive R Archive Network). This approach allowed estimation of observed power for the within-subjects designs used in this study.

Two models were evaluated: (1) the primary 3 × 3 within-subjects design (Treatment × Time), collapsing over emotion, and (2) the full 3×3×2 design (Treatment × Time × Emotion) to examine the impact of emotional valence.

For the 3×3 design, using empirical means, SD (0.605), and a correlation of *r*=0.5, 5000 Monte Carlo simulations with α=.05 and Greenhouse-Geisser correction were performed. The main effect of Time showed high power (100%, *η*²_*p*_=0.639), the Treatment× Time interaction showed moderate power (72.6%, *η*²_*p*_=0.064), and the main effect of Treatment showed low power (15.8%, *η*²_*p*_=0.027). Estimated marginal means contrasts revealed moderate power for day 1 differences (69.8%‐75.0%, Cohen *f*=0.35‐0.37), consistent with the observed short-term interference pattern (full data reported in Table S3 in Multimedia Appendix 1).

For the full 3×3×2 design, incorporating emotion, power was reduced for the Treatment main effect (5.4%, *η*²_*p*_=0.019) and most interaction terms (<34%, *η*²_*p*_<0.04), although Time and Emotion effects remained robust (100%, *η*²_*p*_=0.620 and 0.422, respectively). Certain estimated marginal means contrasts showed moderate detectability (eg, day 1 negative, conscious vs unconscious: 54.6%, *f*=0.29). These results indicate that including emotion reduces sensitivity for small treatment effects, and findings involving this factor should be interpreted cautiously (full data reported in Table S4 in Multimedia Appendix 1).

This analysis supports the focus on temporal dynamics in the 3×3 design as the primary outcome while acknowledging the exploratory nature of analyses in the full 3×3×2 model.

### Data Analysis

Two factorial repeated-measures designs were used to examine the effects of the game-based ShIF. In the first analysis, exposure condition (conscious, unconscious, and control) and test time point (0 hour, 24 hours, and 72 hours) were included as 3-level within-subject factors. This analysis focused on the main effect of exposure condition (ie, conscious < control, unconscious < control), the main effect of time, and their interaction (ie, whether the effect of exposure condition varied across days). All post hoc pairwise comparisons were Bonferroni adjusted.

In the second analysis, emotional valence (positive and negative) was added as an additional within-subject factor, resulting in a 3×3×2 design. This model evaluated whether the short-term memory interference observed immediately after game exposure persisted across the follow-up sessions and whether this pattern differed depending on emotional valence. Post hoc pairwise comparisons were Bonferroni adjusted.

To confirm the robustness of these results, repeated-measures ANCOVA was conducted for each analysis using the same factorial structure, controlling for individual differences in task engagement. Participants’ game score and total feedback duration were included as covariates. Missingness occurred only in these covariates: game score was missing for 3 participants (3/56, 5.4%) and feedback duration for 3 participants (3/56, 5.4%), with 1 participant overlapping, yielding 5 out of 56 unique cases (8.9%). Missingness satisfied missing completely at random (χ^2^_2_=2.87; *P*=.24). Given this covariate-only missingness and the missing completely at random assumption, we analyzed the ANCOVA as a complete-case dataset (n=51) using type III sums of squares with Greenhouse-Geisser corrections where necessary. Post hoc comparisons were Bonferroni adjusted.

### EEG Analysis

To examine the statistical significance of the intervention, we tested whether there was a significant difference between the conscious and unconscious phases in negative images, as well as between the conscious and unconscious phases in positive images. Paired testing was conducted to eliminate individual differences and minimize variations arising from the game progression. Specifically, 40 participants each played the 2 types of games 9 and 18 times in total. All EEG data were segmented within a 2-second time window. Segmentation was performed after preprocessing to avoid filter artifacts at the edges. All studies were performed equally in the 2 groups. To compare the differences between the conscious and unconscious phases, the ith EEG from the conscious phase was paired with the corresponding ith unconscious phase EEG from the same individual in chronological order.

Similarly, to assess the difference between conscious and unconscious states, the EEG from the ith conscious phase was paired with the EEG from the ith unconscious phase for each participant. After data pairing, a 2-sided Wilcoxon signed rank test was performed for each pair. To control for the potential inflation of type I error caused by multiple pairwise comparisons across emotional conditions and EEG channels, a Bonferroni correction was applied.

### Ethical Considerations

This study was reviewed and approved by the institutional review board (IRB) of the Severance Hospital Human Research Protection Center (approval number: 4-2023-0638). All procedures involving human participants were conducted in accordance with the ethical standards of the Declaration of Helsinki and the institutional guidelines issued by the Yonsei University Health System, eIRB-provided document (Multimedia Appendix 2). Written informed consent was obtained from all participants prior to their participation. The consent form described the study purpose, the voluntary nature of participation, the right to withdraw at any time, and the use of anonymized data for research and publication. For the secondary analysis of behavioral and EEG data, the IRB approval explicitly permitted reuse of the deidentified data without requiring additional consent. All participant data were anonymized prior to analysis. Personally identifiable information such as names, email addresses, or voice recordings was removed or replaced with coded identifiers. Verbal responses were transcribed and stored using pseudonymous participant IDs, and all EEG recordings were delinked from any personal identifiers. Data were stored on encrypted institutional servers accessible only to the research team. Participants who took part in the main experiment received monetary compensation of 30,000 KRW (approximately US $20) for their time and effort. Those who participated in the supplementary awareness validation test received a proportional amount based on the duration of their participation. Compensation procedures complied with the institutional policy for human subject research. No identifiable images of individual participants are included in the manuscript or supplementary materials. All figures and photographs display either schematic representations or deidentified data. If any image contained potentially identifiable human features, explicit written consent for publication was obtained from the individual prior to inclusion. The study protocol detailing the full experimental procedures is provided in Multimedia Appendix 3, and the statistical analysis plan used in this study is described in Multimedia Appendix 4.

## Results

### Overview

To validate the effect of game-based ShIF, we designed a game structured in 2 phases: an FP, in which the character’s movement corresponded to the arrow keys, and an RP, in which the character’s movement was inverted. For comparison, among the 36 cue-target pairs prelearned by 56 participants (21 men; mean 23.4 years, SD 1.84 years; Table S1 in Multimedia Appendix 1), half of the pairs were randomly assigned to the Conscious or Unconscious group and presented in the RP, while the remaining pairs were designated as the control group.

### Time-Dependent Effects of Game-Based ShIF

**Figure 2.**
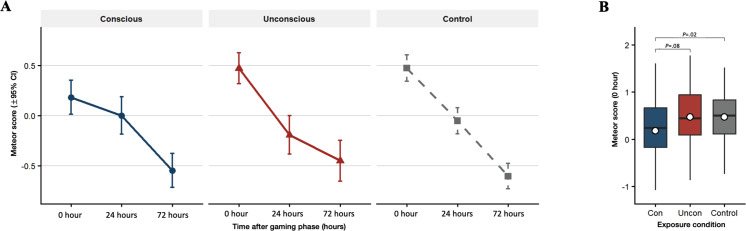
Time-dependent effects of shadow-induced forgetting across exposure conditions. (**A**) Interaction plot showing changes in Metric for Evaluation of Translation with Explicit Ordering (METEOR) scores across exposure conditions (conscious, unconscious, and control) over 3 time points (0 hour, 24 hours, and 72 hours). Error bars represent 95% CIs. (**B**) METEOR scores across exposure conditions at the immediate test (0 hour). The boxes show the IQR with the median. Whiskers represent the minimum and maximum values. White dots denote the mean per condition. Brackets indicate Bonferroni-adjusted 2-tailed paired *t* test.

A two-way repeated-measures ANOVA was conducted with exposure condition (conscious, unconscious, and control) and time (0 hour, 24 hours, and 72 hours) as within-subject factors. A significant interaction between exposure condition and time was observed (*F*_3.62,199.33_=2.7, *P*=.04, *η*^2^_*ρ*_=0.05, 95% CI 0.00-1.00), suggesting that the effect of exposure condition varied across time points, although the main effect of exposure condition was not significant (*F*_1.89,103.74_=0.58, *P*=.55, *η*^2^_*ρ*_=0.01, 95% CI 0.00-1.00) (Figure 2A). Importantly, follow-up analyses revealed that only the immediate test (0 hour) exhibited that the METEOR scores varied significantly across the control, conscious, and unconscious conditions (*F*_1.7,93.59_=4.874, *P*=.01, *η*^2^_*g*_=0.056, 95% CI 0.00-1.00). Specifically, a ShIF effect was observed in the conscious condition, with METEOR scores significantly lower in the conscious group than in the control group (*t*_55_=−2.86, *P*=.02, Cohen *d*=−0.382, 95% CI for *d* −0.658 to −0.110). In contrast, the unconscious group exhibited no significant difference from the control condition (*t*_55_=−0.015, *P*≥.99, Cohen *d*=−0.002, 95% CI for *d* −0.266 to 0.262) and trending higher than the conscious group (*t*_55_=−2.28, *P*=.08, Cohen *d*=−0.304, 95% CI for *d* −0.576 to −0.035). (Figure 2B). The analysis also revealed a strong main effect of time (*F*_1.98,108.93_=84.73, *P*<.001, *η*²_*p*_=0.61, 95% CI 0.51-1.00) indicating a progressive decline in METEOR scores over days.

To ensure the robustness of these findings, the same analyses were conducted while controlling for individual differences in task engagement, using participants’ game scores and total learning time as covariates. The two-way repeated-measures ANCOVA results yielded a comparable pattern, showing a significant interaction between exposure condition and time (*F*_3.54,169.71_=2.64, *P*=.04, *η*^2^_*ρ*_= 0.05, 95% CI 0.00-1.00) and main effect of time (*F*_1.97,94.42_=83.2, *P*<.001, *η*^2^_*ρ*_=0.63, 95% CI 0.54-1.00). Post hoc comparisons again revealed lower METEOR scores in the conscious condition than in the control condition (*t*_48_=−2.975, *P*=.01, Cohen *d*=−0.429, 95% CI for *d* −0.723 to−0.132) in the immediate test (0 hour), confirming that the ShIF effect in the conscious condition remained significant after adjustment for individual differences in task engagement. The conscious condition also tended to score lower than the unconscious condition (*t*_48_=−2.356, *P*=.07, Cohen *d*=−0.340, 95% CI for *d* −0.629 to −0.048), whereas no significant ShIF effect was observed for the unconscious condition when compared with control (*t*_48_=0.037, *P*≥.99). Neither game score (*F*_1,48_=0.18, *P*=.675, *η*^2^_*ρ*_=0.003, 95% CI 0.00-1.00) nor total feedback duration (*F*_1,48_=0.72, *P*=.98, *η*^2^_*ρ*_<0.001, 95% CI 0.00-1.00) showed significant main effects, and none of the interactions involving these covariates were significant (Table S6 in Multimedia Appendix 1).

Applying the same analyses to gist- and BERT-based similarity measures yielded consistent and statistically significant patterns, replicating the results observed with the METEOR scores (Tables S5 and S6 and Figures S3 and S4 in Multimedia Appendix 1).

### Influence of Emotional Valence on the ShIF Effect

**Figure 3.**
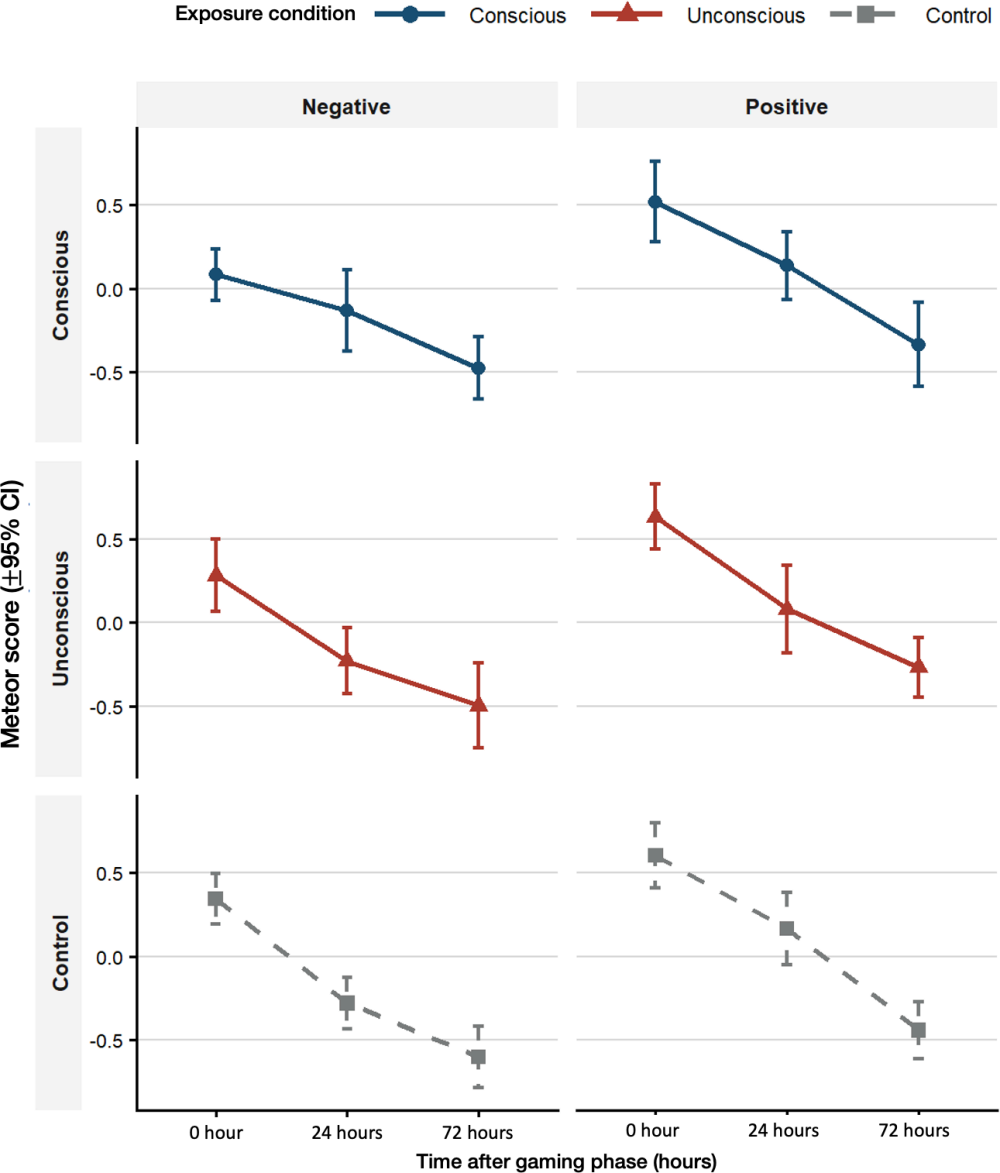
Interaction plot of exposure condition, emotional valence, and time (three-way repeated-measures ANOVA). Mean Metric for Evaluation of Translation with Explicit Ordering scores (±95% CI) are plotted by exposure condition (conscious, unconscious, and control) and emotional valence (positive and negative) across 3 time points (0 hour, 24 hours, and 72 hours). Error bars represent 95% CIs.

To further explore the role of emotion in the ShIF effect, we conducted a three-way repeated-measures ANOVA including emotional valence (positive and negative) as an additional within-subject factor, along with exposure condition and time (Figure 3). The analysis revealed significant main effects of emotional valence (*F*_1,55_=42.43, *P*<.001, *η*^2^_*ρ*_=0.44, 95% CI 0.27-1.00) and time (*F*_2,109.99_=102.91, *P*<.001, *η*^2^_*ρ*_=0.65, 95% CI 0.57-1.00), indicating that recall performance declined over days and was consistently lower for negative than for positive images. Post hoc comparisons for the emotion factor confirmed that negative images were recalled less accurately than positive images across all test sessions (positive valence vs negative valence: *t*_55_=6.514; *P*<.001). Pairwise comparisons for the time factor showed a progressive decline in recall performance from 0 hour to 24 hours (*t*_55_=7.70; *P*<.001) and from 24 hours to 72 hours (*t*_55_=6.628; *P*<.001).

When emotional valence was incorporated into the model, the previously significant interaction between exposure condition and time was no longer statistically significant (*F*_3.63,199.84_=1.34, *P*=.26, *η*^2^_*ρ*_<0.001; see Table S7 in Multimedia Appendix 1 for details). Nevertheless, the inclusion of emotional valence provided additional insight into how the ShIF effect manifested across emotional contexts. At the immediate test (0 hour) following the game intervention, the pattern of results was generally consistent with the previous analysis, as shown by exploratory comparisons within each valence condition. For negative images, the conscious condition (mean 0.083, SD 0.574) showed lower mean METEOR scores than the control condition (mean 0.342, SD 0.57) (mean difference=−0.259, 95% CI −0.542 to 0.024), whereas for positive images, the conscious (mean 0.517, SD 0.9) and control (mean 0.602, SD 0.726) conditions were more comparable (mean difference=−0.085, 95% CI −0.452 to 0.282). Although formal post hoc comparisons were not conducted, this descriptive trend suggests that the short-term ShIF effect may have been more pronounced for negative stimuli under conscious exposure.

To account for potential variability in task engagement, we further included game score and total feedback duration as covariates in a repeated-measures ANCOVA to control for potential baseline differences. The adjusted model yielded highly comparable results with ANOVA, confirming that the main effects of emotion (*F*_1,48_=43.94, *P*<.001, *η*^2^_*ρ*_=0.44, 95% CI 0.27-1.00) and time (*F*_1,48_=97.93, *P*<.001, *η*^2^_*ρ*_=0.65, 95% CI 0.57-1.00) remained robust after controlling for these factors. Exposure conditions (*F*_1,84,88.5_=0.1, *P*=.89, *η*^2^_*ρ*_=0.004, 95% CI 0.00-1.00) and the interactions remained nonsignificant (Table S8 in Multimedia Appendix 1). Neither game score (*F*_1,48_=0.04, *P*=.84, *η*^2^_*ρ*_<0.001) nor total feedback duration (*F*_1,48_=0.72, *P*=.40, *η*^2^_*ρ*_=<0.001) exerted significant effects, supporting that the observed effects were independent of task performance or engagement.

Applying the same analyses to gist and BERT measures yielded a consistent pattern replicating the METEOR-based results (Tables S7-S11 in Multimedia Appendix 1). Full details are provided in Multimedia Appendix 1.

### EEG Assessments

**Figure 4.**
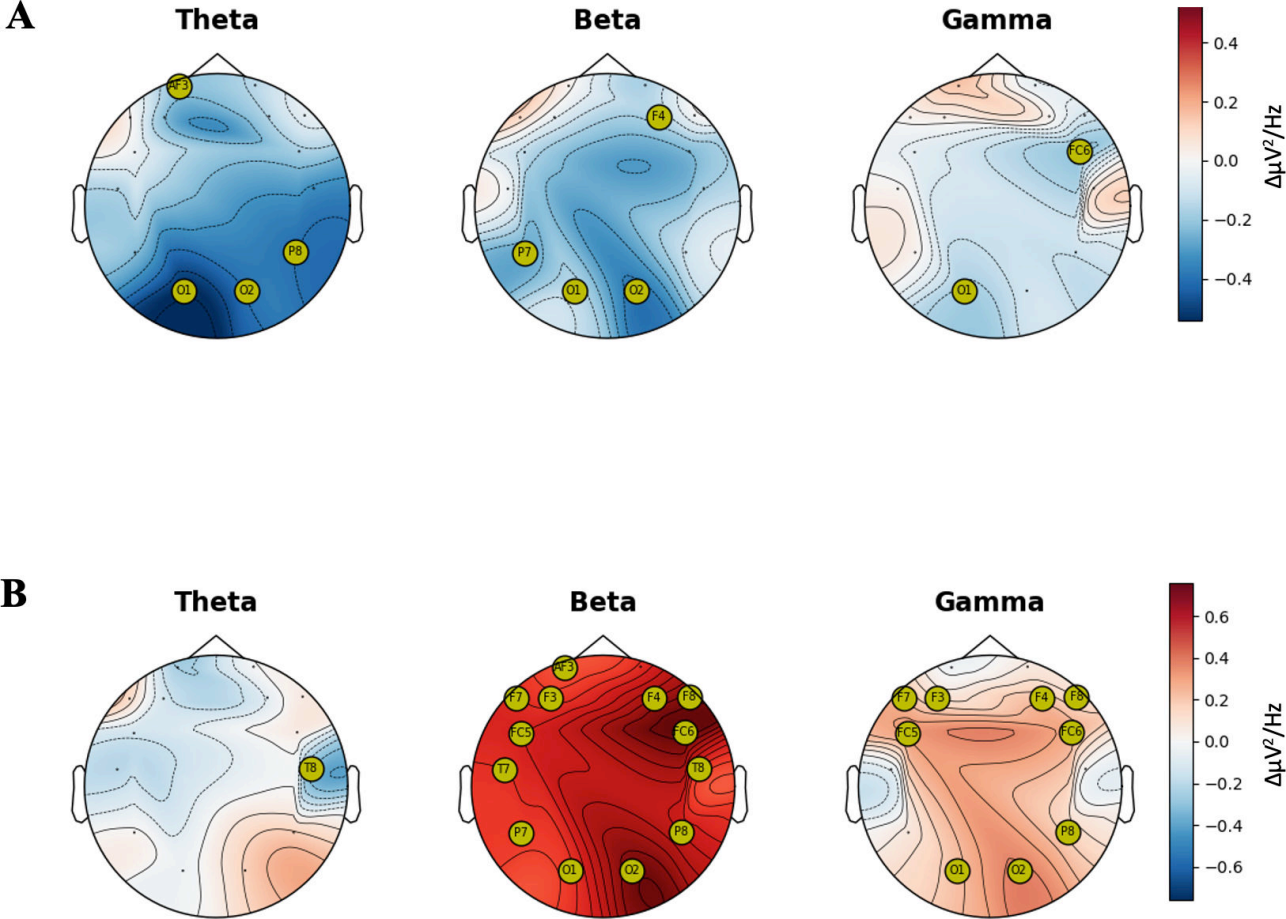
The median difference in electroencephalography power spectrum density between groups. The median of the difference in the power spectrum density between groups is plotted topographically on the scalp with sensor locations. (**A**) The median of the difference in the power spectrum density between “Conscious” and “Unconscious.” (**B**) The median of the difference in the power spectrum density between “Negative” and “Positive.” The channels marked with yellow circles are those where the Wilcoxon signed-rank test resulted in *P* values <.05. White areas indicate no difference between the 2 groups, red areas indicate a higher median power spectrum density for the negative or conscious group, and blue areas indicate a higher median power spectrum density for the positive or unconscious group.

EEG measurements were conducted on the participants during the game, and power spectrum densities were compared. Details of the statistical methods and measurement techniques are provided in the “Methods” section. First, a comparison of EEG spectrum activation between the unconscious and conscious phases showed a relatively higher activation in the unconscious phase for EEG channels near the occipital region of the beta and theta spectra, likely due to stress from flickering stimuli [36,37]. A topographical plot of the power spectrum density difference between the conscious and unconscious phases is shown in Figure 4A. The channels with significant differences are marked with yellow circles. Results from the Wilcoxon signed rank test demonstrated higher activation patterns during the unconscious game, especially in channels near the occipital region. (O1: *P*=.02 and O2: *P*=.009 for theta spectrum, O1: *P*=.037 and O2: *P*=.02 for beta spectrum, and O1: *P*=.004 for gamma spectrum) (Table S12 in Multimedia Appendix 1).

Second, power spectrum analysis of EEG signals recorded in response to positive and negative images revealed that the brainwave spectrum bands exhibited significantly higher activation when viewing negative images than when viewing positive ones (Figure 4B). Notably, a significant difference was observed in the right frontal beta band power. This finding aligns with the previously observed tendency of memory attenuation [38] for negative images, further emphasizing the ShIF effect. Indeed, the results of the Wilcoxon signed rank test demonstrated highly significant activation patterns when revealed in negative images. For the beta waves, highly significant activation patterns were found in the right frontal channels (FC6: *P*<.001, F4: *P*<.001, and F8: *P*<.001). A significant increase in beta waves was also noted in the left frontal region (AF3: *P*=.03, F3: *P*=.002, F7: *P*<.001, FC5: *P*<.001, and T7: *P*<.001) as well as in the occipital and parietal regions (O1: *P*=.006, O2: *P*<.001, P8: *P*<.001, and P7: *P*=.009). For the gamma band, significantly increased activation was observed in the posterior region (O1: *P*<.001, O2: *P*<.001, and P8: *P*=.002) and in the centrofrontal area (F7: *P*<.001, C5: *P*<.001, F3: *P*=.009, F4: *P*<.001, FC6: *P*<.001, and F8: *P*=.03) (Table S13 in Multimedia Appendix 1).

## Discussion

### Principal Findings

In this study, we examined how exposure conditions and image valence influenced ShIF across 3 time points. Our work builds on the ShIF framework introduced by Zhu et al [5,18], which posits that unrelated episodic memories reactivated during the amnesic shadow, a period of reduced hippocampal activity, can be weakened. We successfully observed short-term ShIF for consciously cued images during a period of game-based amnesic shadows, but the effects of unconsciously cued images differed from those of a previous study [18], highlighting the need for further research on unconscious processing. Also, to our knowledge, this study is the first to show that ShIF occurs selectively for negative memories. Using EEG data, we provided the first evidence that inhibitory neural activity increases during the suppression of negative memories, directly supporting the mechanism underlying ShIF. Furthermore, by tracking the effects of ShIF over several days, we expanded ShIF to different temporal and emotional contexts.

Our first and second hypotheses, which state that ShIF effects will be significant in (1) both short- and long-term contexts and (2) in both conscious and unconscious exposure conditions, were not supported. In the short term (0 hour), we observed significant ShIF effects for consciously cued images, as evidenced by lower memory performance scores compared with control images, consistent with previous findings [5,18]. However, unlike a previous study [18], unconsciously cued images did not show significant ShIF effects. EEG analysis revealed that frontal beta power, a marker of inhibitory mechanisms linked to hippocampal downregulation [38-41], was not significantly different between conscious and unconscious conditions, suggesting that hippocampal activity may be similarly downregulated across conditions [42]. The lack of ShIF effects in the unconscious condition suggests that different interfering factors in the memory suppression process need further examination. Stress induced by the unconscious exposure condition in our game may explain this discrepancy. Specifically, the visual flickering used to deliver unconscious stimuli can act as a stressor [36,37], potentially impairing the effectiveness of memory suppression [43,44]. Unlike traditional paradigms, the flickering stimuli in our game were designed to be perceived unconsciously but may also function as visual distractors, elevating stress levels. Our EEG analysis shows increased occipital theta and beta band activity during the unconscious condition associated with stress responses to the visual stimuli [45-47], suggesting that the flickering stimuli, intended to deliver unconscious cues, may have acted as stress-inducing visual distractions, disrupting the ShIF mechanisms required for effective memory suppression. Future research should explore alternative unconscious exposure techniques to reduce stress while preserving the efficacy of the ShIF paradigm.

Over the long term, by 72 hours, the ShIF effect on consciously cued images diminished, with no significant differences observed at 72 hours. This finding is consistent with previous research showing that intentional forgetting effects tend to dissipate over time [48,49]. Although the ShIF effect for unconsciously cued images was not statistically significant, the trend observed at 24 hours suggests a potential delayed effect. This may tentatively align with the Negative Compatibility Effect theory [50] which proposes that inhibitory responses to subliminally activated memories can emerge over time. However, given the lack of statistical significance, a larger sample size is needed to verify this possible delayed effect.

Furthermore, we found that ShIF effects were significant only for images with negative valence. Negative memories are more likely to be intrusively reactivated and are more susceptible to suppression [11,12]. Our EEG results corroborated this finding, showing increased gamma band activity during negative cue presentation associated with processing unpleasant, high-arousal stimuli [51,52]. Significantly enhanced beta power, including in the frontal region, is linked to intentional forgetting and inhibitory processes [38-41]. This suggests that negative memories elicit stronger suppression processes, making them more susceptible to ShIFs.

In addition, memory performance scores were consistently and significantly lower for negative images at all time points (0 hour, 24 hours, and 72 hours). Higher arousal from negative stimuli may narrow attention, explaining their consistently lower recall and the lasting influence of valence on memory retention [53].

Our research has 3 main contributions to both the theoretical understanding and the practical applications of memory suppression mechanisms. First, by comparing the effects of ShIF under various conditions of awareness and emotional valence, we demonstrated that ShIF is more effective for consciously cued negative memories. Nevertheless, while our paradigm may have theoretical relevance for understanding intrusive memory processes in disorders such as posttraumatic stress disorder (PTSD), depression, and obsessive-compulsive disorder [54-59], cautious clinical application is needed. The emotional intensity and consolidation strength of real traumatic memories are far greater than those of experimentally induced ones [60], and inhibitory control may differ across clinical populations. Our findings primarily highlight a methodological and conceptual framework in a naturalistic, game-based setting, rather than providing direct clinical evidence.

Second, we introduced a novel game-based approach to induce amnesic shadows using reversed directional keys to trigger memory suppression. This method builds on the idea that suppressing motor responses engages inhibitory processes similar to those involved in memory suppression [12]. Our approach effectively induces the amnesic shadow without relying on the traditional TNT paradigm through the motor conflict in our game that imitates working memory challenges and motor sequence suppression in previous studies [15,61]. Moreover, this game-based method has several advantages. It reduces the experimenter bias inherent in the TNT paradigm [62] and may be more applicable in clinical situations where directly suppressing unwanted memories is challenging. The intuitiveness of games may be more engaging and less confrontational for individuals reluctant to participate in traditional therapeutic methods.

Finally, we used the METEOR score as a more accurate and objective evaluation metric for assessing memory performance [63,64]. Although METEOR is not a conventional memory metric, our validation analyses revealed strong agreement between METEOR and both human and embedding-based similarity scores, supporting its reliability as a semantic measure. The consistent—but sometimes more liberal—sensitivity of METEOR likely reflects its higher tolerance for semantic paraphrasing rather than inflated detection of differences.

### Limitations

Despite these contributions, our study has limitations. First, individual differences among participants may have influenced the results in multiple ways. Although we implemented a feedback cycle to ensure consistent memorization, variations in participants’ concentration, gaming experience, and skill levels may have affected their memory performance [65]. Likewise, no formal psychiatric or trauma-related screening (eg, PTSD, depression, or anxiety measures) was conducted beyond a basic prescreening question about current medical or psychological treatment. Unassessed differences in trauma history or emotional sensitivity could also have shaped responses to negative images and subsequent memory outcomes. Future studies should control for these individual-level factors by diversifying game tasks to sustain engagement and incorporating standardized mental health screening.

Second, methodological limitations arise from the statistical power and design of the study. The experiment involved 36 cue-target pairs distributed across a 3×3×2 within-subjects design, resulting in only 2‐3 items per cell. Although the total number of observations (n=56 participants × 36 items = 2016) appears large, the low item count per cell amplifies variability and limits sensitivity to small treatment effects, particularly for interactions involving emotional valence or unconsciously cued images. To examine this, we conducted a simulation-based post hoc power analysis using the Superpower R package (Comprehensive R Archive Network). Results indicated that the primary 3×3 design (Treatment × Time) had high power to detect medium-sized effects, particularly for day 1 contrasts where ShIF effects for consciously cued images were observed. In contrast, incorporating emotional valence in the full 3×3×2 design substantially reduced power for small treatment effects and interactions, which likely contributed to nonsignificant trends for unconsciously cued images at 24 hours. These findings highlight that while the short-term ShIF effects for consciously cued images are robust, caution is warranted in interpreting nonsignificant effects in more complex combinations, and future studies with larger samples and more items per cell are needed to fully investigate potential delayed ShIF effects under unconscious cueing.

Third, our analysis primarily focused on the weakening of cue-target associations rather than directly measuring the intrinsic strength of the memories. Although disrupting these associations is clinically relevant for reducing involuntary recall of traumatic memories [55], future research should consider the recall of the memory using independent cues to determine whether the memories themselves are weakened. In addition, recall accuracy in this study was assessed using a unified description derived from a small independent sample (n=12) rather than participant-specific references. While this approach ensured consistency across items, it likely reflected the “typical” recallable content of each image rather than each participant’s unique reconstruction. We intentionally avoided collecting participant-specific descriptions before the main task, as verbalizing image content could have influenced later recall performance. Future studies should incorporate individualized reference descriptions in separate pre- or postsessions and better isolate genuine memory strength and recall accuracy.

Furthermore, the usage of image stimuli from multiple sources and the limited norming procedure may be considered. Although this approach allowed us to select images that met specific criteria—namely, containing a context-irrelevant object suitable for use as a cue—it also introduced variability in stimulus characteristics and restricted the generalizability of the emotional ratings due to the small norming sample. Several prior studies have similarly relied on stimuli from multiple databases rather than a single standardized source [22,23], suggesting the feasibility of this approach. This approach also enabled selecting images that met specific criteria—namely, containing a context-irrelevant object suitable for use as a cue—but led to variability in stimulus characteristics and restricted the generalizability of the emotional ratings due to the small norming sample. In addition, the absence of a neutral baseline condition limits the ability to determine whether the observed effects are truly valence-specific or reflect general emotional processing. This decision was made to increase sensitivity to positive-negative contrasts and to avoid potential ambiguity associated with “neutral” stimuli, which are known to vary widely across individuals and contexts [66,67]. Future research should address these issues by recruiting a larger and more diverse group of raters, developing a unified standardized database that fulfills both emotional and contextual control requirements, and incorporating neutral stimuli to establish a clearer reference point for valence-dependent effects. Future research should recruit a larger and more diverse group of raters, developing a unified standardized database that fulfills both emotional and contextual control requirements, and incorporate neutral stimuli to establish a clearer reference point for valence-dependent effects.

Finally, our study focused on the effects of a single, 8-second game-induced amnesic shadow on recently learned memories. This limited intervention may not fully capture the potential of ShIF. Intentional forgetting may have long-term effects on consolidated memories [68], and multiple attempts at suppression can amplify the extent of forgetting [8,69-73]. Future studies should explore whether repeated game-based ShIF sessions over extended periods have greater memory suppression effects, which may enhance the clinical applicability of ShIF in long-term memory management.

### Conclusions

Our study demonstrates that ShIF is modulated by the awareness and valence of memories and that ShIF can be efficiently induced in the short term by a game-based approach, especially for negative and consciously cued images. EEG analyses provided critical validation, revealing increased inhibitory neural activity during the suppression of negative memories and directly supporting the neural mechanisms underlying ShIF. The delayed ShIF effect observed for unconsciously cued images highlights the complex dynamics of memory suppression over time. These findings contribute to a deeper understanding of the memory suppression mechanisms of ShIF and open new avenues for its practical application in clinical settings.

Importantly, this study confirms that suppression effects can be obtained through a game-based paradigm, suggesting a more engaging and ecologically valid alternative to traditional explicit suppression tasks. Moreover, unlike previous studies that have primarily focused on transient effects limited to consciously processed negative stimuli, our investigation examined emotional valence, exposure condition, and temporal dynamics. We thereby extend the theoretical and practical scope of ShIF. Taken together, these results provide preliminary evidence supporting the potential translation of ShIF mechanisms into digital therapeutic applications, such as game-based interventions for PTSD and related conditions.

## Supplementary material

10.2196/76946 Multimedia Appendix 1 Supplementary participant characteristics and detailed numerical outputs (eg, descriptive statistics, ANCOVA tables, and post hoc results).

10.2196/76946 Multimedia Appendix 2 Information for research subjects consent form.

10.2196/76946 Multimedia Appendix 3 Study protocol.

10.2196/76946 Multimedia Appendix 4 Statistical analysis plan.

10.2196/76946 Checklist 1 TREND statement checklist.

## Abbreviations

EEG: electroencephalography
FP: forward phase
IRB: institutional review board
METEOR: Metric for Evaluation of Translation with Explicit Ordering
NT: No-think
PTSD: posttraumatic stress disorder
RP: reverse phase
ShIF: shadow-induced forgetting
TNT: Think/No-Think
TREND: Transparent Reporting of Evaluations with Nonrandomized Designs
