# Supplementary material for: Shadow-Induced Forgetting in a Game-Based Paradigm on Nonclinical Adults and Its Effects on Consciousness, Emotional Valence, and Temporal Dynamics: Crossover Study
Source: JMIR Serious Games. 2025 Dec 30;13:e76946. doi: 10.2196/76946 (PMC12753131; doi:10.2196/76946)
Supplement: Multimedia Appendix 1 [file games-v13-e76946-s001.docx]

**Multimedia Appendix 1**

**Figure S1.** Participant flow.


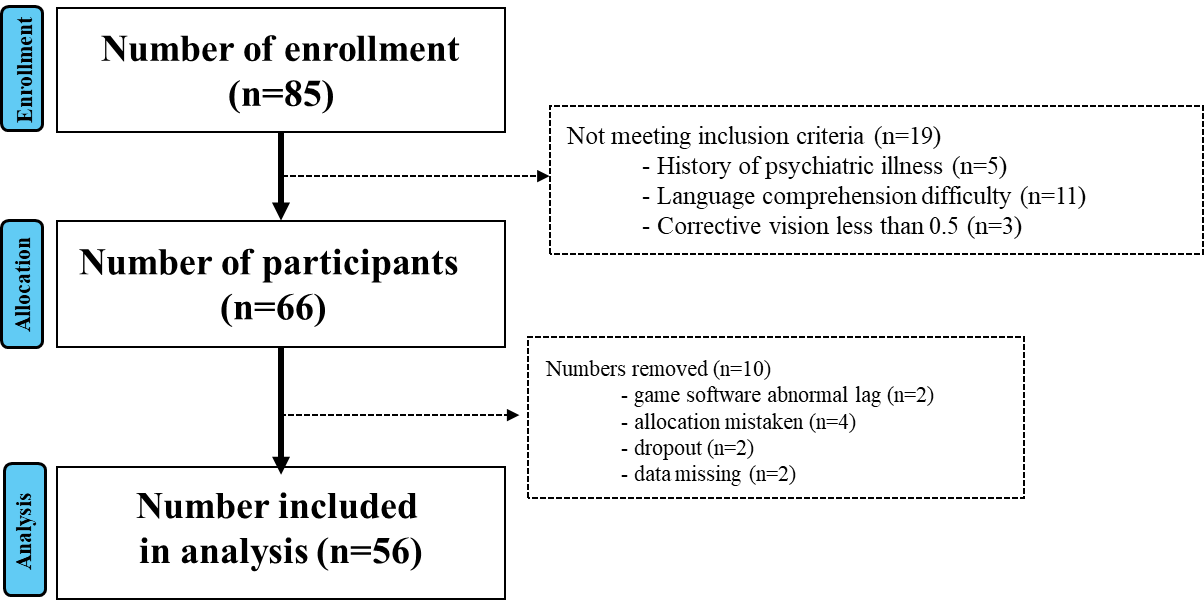


**Figure S2.** Game user interface.

The images below represent the user interface of the game’s forward phase and reverse phase, respectively.


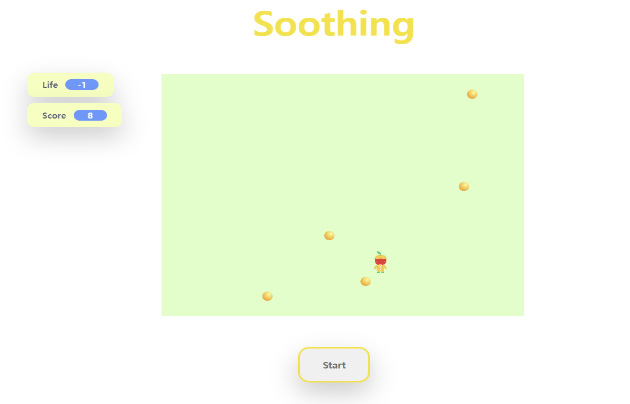

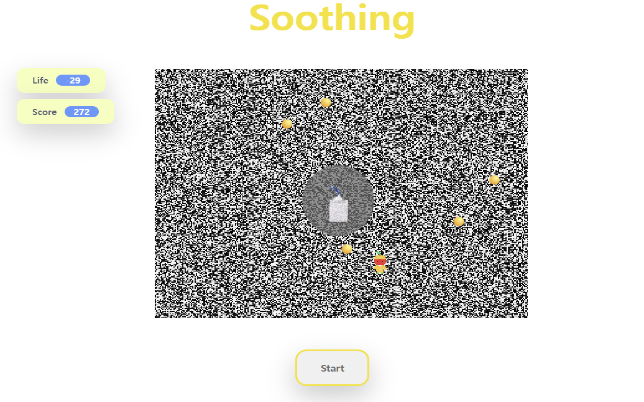


**Table S1.** Demographic profile.

| **Category** | | **All Participants(N=56)** | | |  |
| --- | --- | --- | --- | --- | --- |
|  | **Age,years** |  |  | 23.37(1.84) |  |
|  | **Gender(%)** | Female | | 62.5 |  |
|  | **Corrected Vision** |  |  | 0.99(0.21) |  |

**Table S2.** Correlation coefficients between scoring metrics.

| **Comparison** | **n** | **r** | **95% CI** | **p-value (FDR-adjusted)** |
| --- | --- | --- | --- | --- |
| METEOR vs GIST | 2016 | 0.773 | [0.755, 0.790] | <0.001 (<0.001) |
| METEOR vs BERT | 2016 | 0.772 | [0.753, 0.789] | <0.001 (<0.001) |

**Note.** n = sample size; r = Pearson correlation coefficient; CI = confidence interval; p-values are reported with FDR (Benjamini–Hochberg) adjustment.

**Table S3.** Observed power and effect sizes for 3×3 design (N=56).

| Effect/Contrast | Power (%) | Effect Size | Notes |
| --- | --- | --- | --- |
| Omnibus ANOVA |  | Partial η² |  |
| Treatment | 15.8 | 0.027 (small) | Low overall |
| Time | 100.0 | 0.639 (large) | Strong temporal changes |
| Treatment × Time | 72.6 | 0.064 (medium) | Moderate interaction |
| EMM (Treatment within Time) |  | Cohen's f |  |
| Day 1: con - ctl | 69.8 | 0.348 (medium) | Detectable Day 1 differences |
| Day 1: con - unc | 75.0 | 0.366 (medium) | Strongest contrast |
| Day 1: ctl - unc | 6.4 | 0.113 (small) | Negligible |
| Day 2: con - ctl | 6.6 | 0.114 (small) | Low |
| Day 2: con - unc | 33.2 | 0.221 (small-medium) | Moderate |
| Day 2: ctl - unc | 19.8 | 0.174 (small) | Low |
| Day 4: con - ctl | 6.4 | 0.120 (small) | Low |
| Day 4: con - unc | 12.8 | 0.143 (small) | Low |
| Day 4: ctl - unc | 24.6 | 0.184 (small) | Low |

Note. Emotion collapsed; based on experimental data; 5,000 simulations.

**Table S4.** Observed power and effect sizes for 3×3×2 design (N=56).

| Effect/Contrast | Power (%) | Effect Size | Notes |
| --- | --- | --- | --- |
| Omnibus ANOVA |  | Partial η² |  |
| Treatment | 5.4 | 0.019 (small) | Low overall |
| Time | 100.0 | 0.620 (large) | Strong temporal changes |
| Emotion | 100.0 | 0.422 (large) | Strong emotion differences |
| Treatment × Time | 33.8 | 0.039 (small) | Moderate-weak |
| Treatment × Emotion | 2.8 | 0.017 (small) | Negligible |
| Time × Emotion | 23.0 | 0.038 (small) | Weak |
| Treatment × Time × Emotion | 13.8 | 0.026 (small) | Weak three-way |
| EMM Treatment Contrasts (within Time × Emotion) |  | Cohen's f |  |
| Day1_neg con - ctl (p_1) | 29.4 | 0.210 (small-medium) | Moderate in Day1 neg |
| Day1_neg con - unc (p_2) | 54.6 | 0.294 (medium) | Highest detectable |
| Day1_neg ctl - unc (p_3) | 6.6 | 0.117 (small) | Negligible |
| Day2_neg con - ctl (p_4) | 12.0 | 0.134 (small) | Low |
| Day2_neg con - unc (p_5) | 8.2 | 0.127 (small) | Low |
| Day2_neg ctl - unc (p_6) | 6.4 | 0.115 (small) | Low |
| Day4_neg con - ctl (p_7) | 11.4 | 0.134 (small) | Low |
| Day4_neg con - unc (p_8) | 16.0 | 0.163 (small) | Low |
| Day4_neg ctl - unc (p_9) | 7.2 | 0.122 (small) | Low |
| Day1_pos con - ctl (p_10) | 4.6 | 0.117 (small) | Low |
| Day1_pos con - unc (p_11) | 4.0 | 0.109 (small) | Low |
| Day1_pos ctl - unc (p_12) | 6.6 | 0.120 (small) | Low |
| Day2_pos con - ctl (p_13) | 4.2 | 0.104 (small) | Low |
| Day2_pos con - unc (p_14) | 15.8 | 0.150 (small) | Low |
| Day2_pos ctl - unc (p_15) | 10.2 | 0.121 (small) | Low |
| Day4_pos con - ctl (p_16) | 6.0 | 0.118 (small) | Low |
| Day4_pos con - unc (p_17) | 10.0 | 0.133 (small) | Low |
| Day4_pos ctl - unc (p_18) | 29.6 | 0.209 (small-medium) | Moderate in Day4 pos |

Note. Based on experimental data (2,016 obs); 5,000 simulations.

**Table S5.** Results for two-way repeated measures ANOVA(N=56).

|  | Meteor | | | |  | Gist | | | |  | Bert | | | |
| --- | --- | --- | --- | --- | --- | --- | --- | --- | --- | --- | --- | --- | --- | --- |
|  | MSE | F | η²ₚ | p |  | MSE | F | η²ₚ | p |  | MSE | F | η²ₚ | p |
| Exposure condition | 0.40 | 0.58 | 0.01 | 0.552 |  | 0.50 | 0.08 | 0.001 | 0.91 |  | 0.43 | 0.17 | 0.003 | 0.834 |
| time | 0.41 | 84.73 | 0.61 | <0.001 |  | 0.68 | 50.32 | 0.48 | <0.001 |  | 0.6 | 70.59 | 0.56 | <0.001 |
| Exposure condition : Time | 0.46 | 2.70 | 0.05 | 0.037 |  | 0.45 | 2.58 | 0.04 | 0.043 |  | 0.46 | 3.03 | 0.05 | 0.021 |

Mean Square Error (MSE), F-values, partial eta squared (η²ₚ), and p-values are reported for each effect.

**Table S6.** Results for two-way repeated measures ANCOVA (N=56).

|  | Meteor | | | |  | Gist | | | |  | Bert | | | | |
| --- | --- | --- | --- | --- | --- | --- | --- | --- | --- | --- | --- | --- | --- | --- | --- |
|  | MSE | F | η²ₚ | p |  | MSE | F | η²ₚ | p |  | MSE | F | η²ₚ | p |  |
| Game score | 0.15 | 0.18 | 0.003 | 0.675 |  | 0.26 | 1.18 | 0.02 | 0.282 |  | 0.22 | 0.06 | 0.001 | 0.807 |  |
| Feedback duration | 0.15 | 0.00 | <0.001 | 0.983 |  | 0.26 | 3.43 | 0.07 | 0.07 |  | 0.22 | 0.4 | 0.008 | 0.529 |  |
| Exposure condition | 0.43 | 0.48 | 0.01 | 0.61 |  | 0.54 | 0.04 | <0.001 | 0.955 |  | 0.46 | 0.13 | 0.002 | 0.87 |  |
| time | 0.41 | 83.2 | 0.63 | <0.001 |  | 0.69 | 47.95 | 0.5 | <0.001 |  | 0.57 | 68.6 | 0.59 | <0.001 |  |
| Exposure condition : Time | 0.45 | 2.64 | 0.05 | 0.042 |  | 0.47 | 1.68 | 0.03 | 0.165 |  | 0.46 | 2.49 | 0.05 | 0.048 |  |
| Game score: Time | 0.38 | 1.12 | 0.02 | 0.328 |  | 0.69 | 0.98 | 0.02 | 0.371 |  | 0.57 | 2.85 | 0.06 | 0.066 |  |
| Feedback duration: Time | 0.38 | 2.84 | 0.06 | 0.064 |  | 0.69 | 3.62 | 0.07 | 0.035 |  | 0.57 | 2.83 | 0.03 | 0.067 |  |
| Game score: Exposure condition | 0.43 | 0.03 | <0.001 | 0.963 |  | 0.54 | 0.46 | 0.009 | 0.621 |  | 0.46 | 0.58 | 0.01 | 0.556 |  |
| Feedback duration: Exposure condition | 0.43 | 0.58 | 0.01 | 0.554 |  | 0.54 | 0.53 | 0.01 | 0.577 |  | 0.46 | 1.25 | 0.03 | 0.289 |  |
| Game score: exposure condition: time | 0.45 | 1.55 | 0.03 | 0.197 |  | 0.47 | 1.68 | 0.01 | 0.165 |  | 0.46 | 0.65 | 0.01 | 0.623 |  |
| Feedback duration: exposure condition: time | 0.45 | 1.41 | 0.03 | 0.235 |  | 0.47 | 1.80 | 0.04 | 0.14 |  | 0.46 | 1.12 | 0.02 | 0.346 |  |

Mean Square Error (MSE), F-values, partial eta squared (η²ₚ), and p-values are reported for each effect.

**Figure S3.** Interaction plot: exposure condition x time (Gist, Bert score).


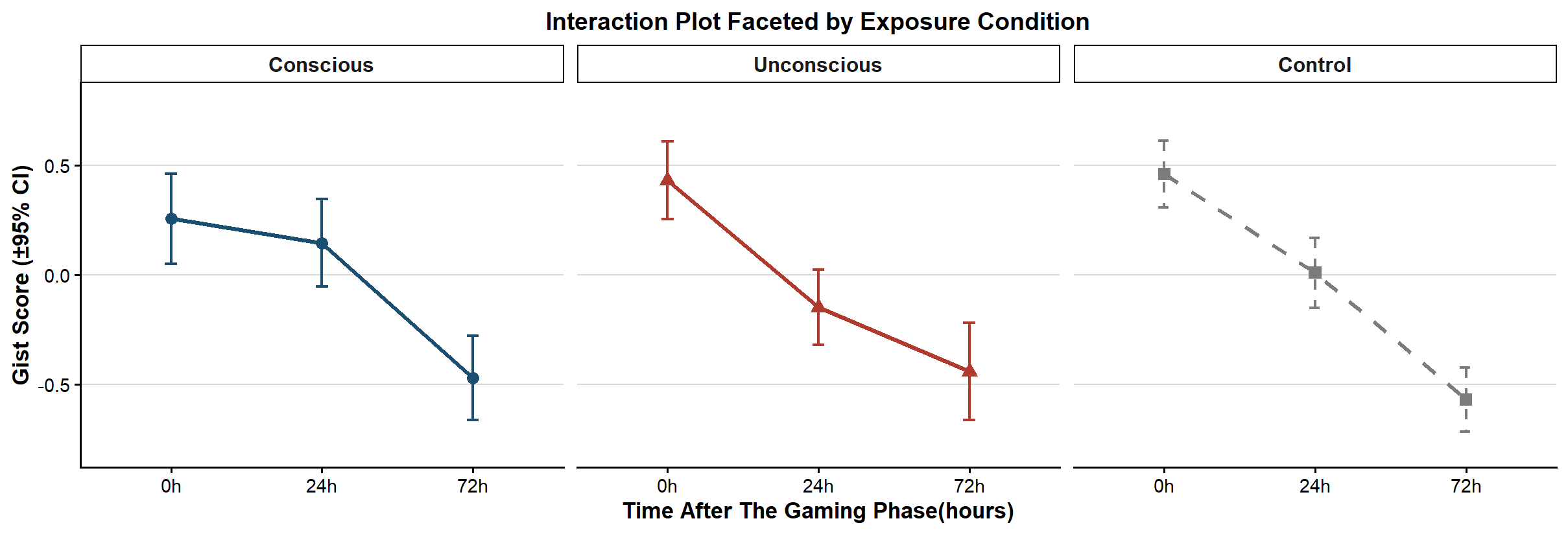


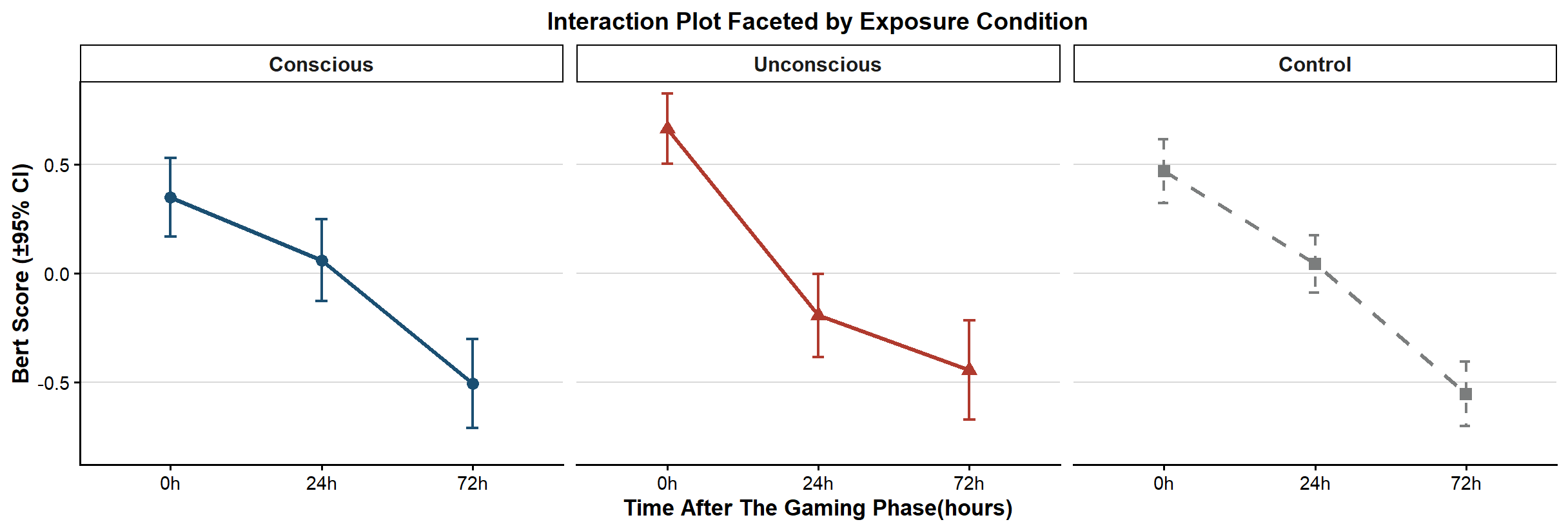


**Figure S4.** Interaction plot: exposure condition x valence x time (Gist, Bert score).

| 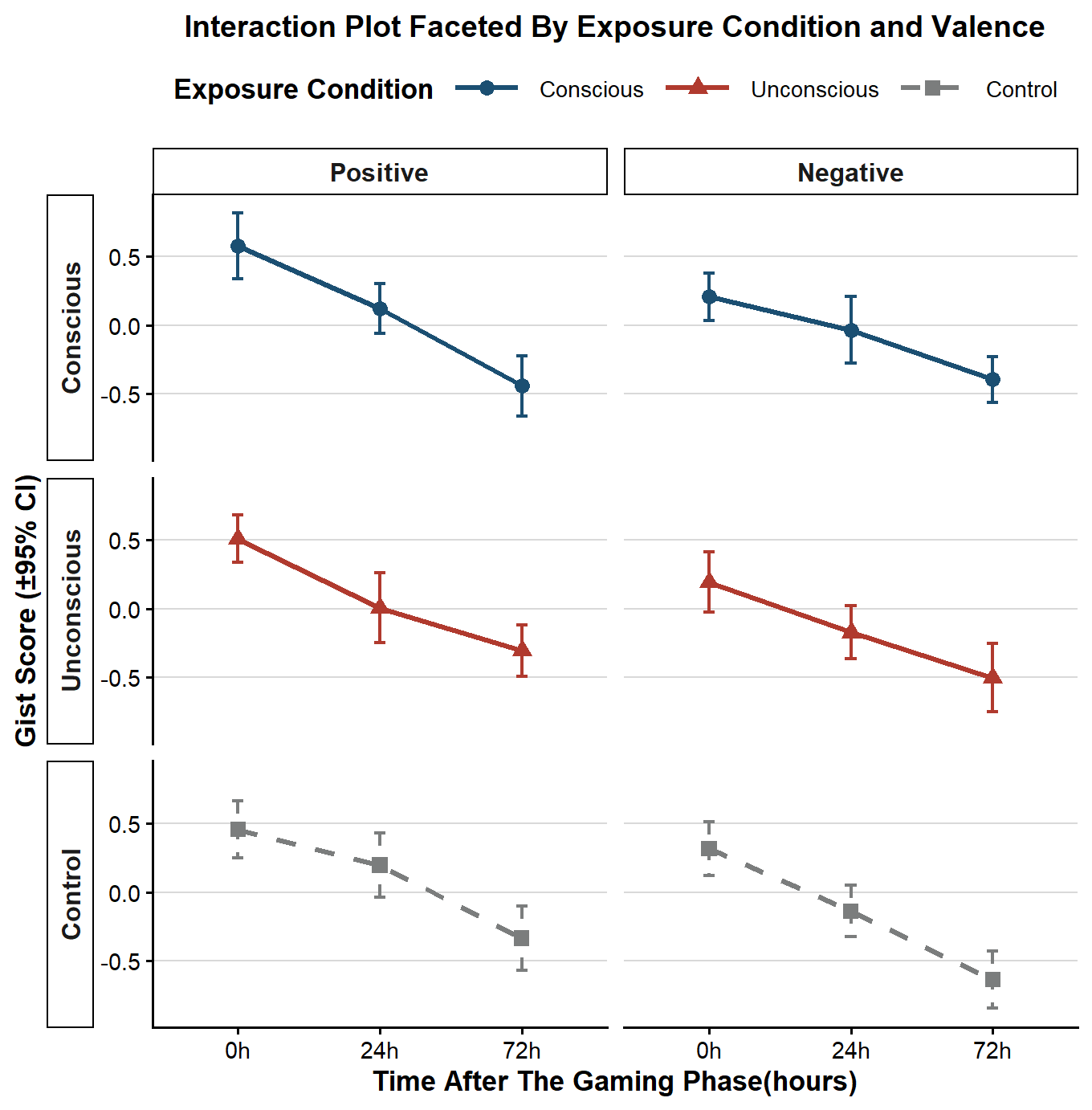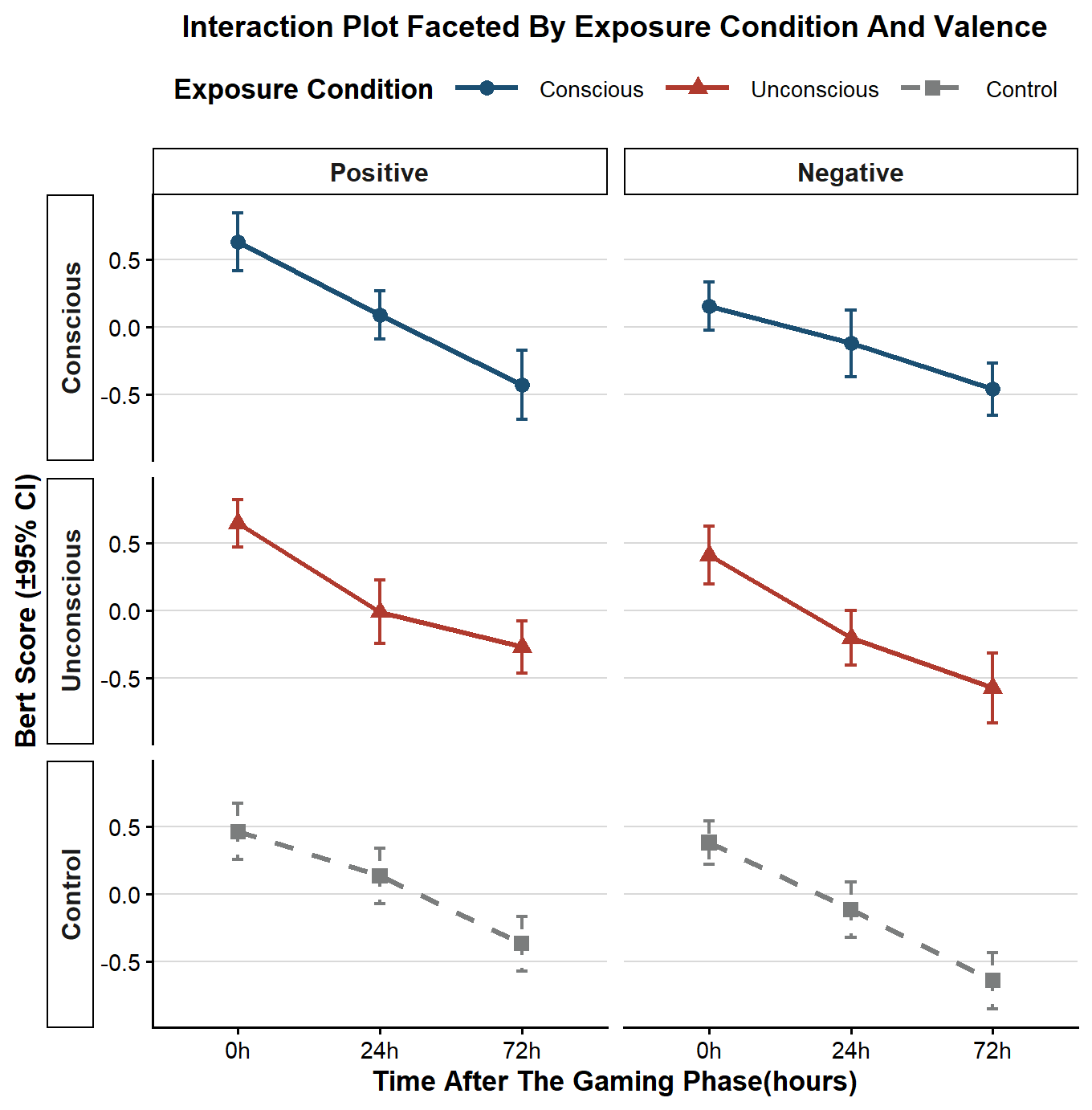 |
| --- |

**Table S7.** Results for three-way repeated measures ANOVA(N=56).

|  | Meteor | | | |  | Gist | | | |  | Bert | | | |
| --- | --- | --- | --- | --- | --- | --- | --- | --- | --- | --- | --- | --- | --- | --- |
|  | MSE | F | η²ₚ | p |  | MSE | F | η²ₚ | p |  | MSE | F | η²ₚ | p |
| Exposure condition | 0.57 | 0.24 | 0.004 | 0.769 |  | 0.64 | 0.36 | 0.006 | 0.696 |  | 0.58 | 0.11 | 0.002 | 0.886 |
| Valence | 0.5 | 42.43 | 0.44 | <0.001 |  | 0.46 | 25.51 | 0.32 | <0.001 |  | 0.39 | 33.63 | 0.38 | <0.001 |
| time | 0.59 | 102.91 | 0.65 | <0.001 |  | 0.73 | 77.49 | 0.58 | <0.001 |  | 0.76 | 92.09 | 0.63 | <0.001 |
| Exposure condition : Valence | 0.93 | 0.01 | <0.001 | 0.989 |  | 0.82 | 0.27 | 0.004 | 0.757 |  | 0.84 | 0.06 | 0.001 | 0.917 |
| Exposure condition : Time | 0.69 | 1.34 | 0.02 | 0.258 |  | 0.55 | 0.56 | 0.01 | 0.681 |  | 0.62 | 0.97 | 0.02 | 0.419 |
| Valence : Time | 0.74 | 0.1 | 0.02 | 0.336 |  | 0.8 | 0.41 | 0.007 | 0.665 |  | 0.86 | 0.1 | 0.001 | 0.897 |
| Exposure Condition : Valence : Time | 0.56 | 0.47 | 0.008 | 0.749 |  | 0.66 | 1.07 | 0.02 | 0.371 |  | 0.6 | 1.47 | 0.03 | 0.214 |

Mean Square Error (MSE), F-values, partial eta squared (η²ₚ), and p-values are reported for each effect.

**Table S8.** Results for three-way repeated measures ANCOVA(N=56).

|  | Meteor | | | |  | Gist | | | |  | Bert | | | |
| --- | --- | --- | --- | --- | --- | --- | --- | --- | --- | --- | --- | --- | --- | --- |
|  | MSE | F | η²ₚ | p |  | MSE | F | η²ₚ | p |  | MSE | F | η²ₚ | p |
| Exposure condition | 0.63 | 0.1 | 0.002 | 0.893 |  | 0.66 | 0.28 | 0.006 | 0.745 |  | 0.6 | 0.13 | 0.003 | 0.87 |
| Valence | 0.46 | 43.94 | 0.48 | <.001 |  | 0.46 | 21.34 | 0.31 | <.001 |  | 0.37 | 30.36 | 0.39 | <.001 |
| Time | 0.57 | 97.39 | 0.67 | <.001 |  | 0.74 | 73.71 | 0.61 | <.001 |  | 0.71 | 92.17 | 0.66 | <.001 |
| Exposure condition:Valence | 0.95 | 0 | <.001 | 0.992 |  | 0.81 | 0.26 | 0.006 | 0.773 |  | 0.82 | 0.05 | 0.001 | 0.932 |
| Exposure condition:Time | 0.67 | 1.4 | 0.03 | 0.241 |  | 0.57 | 0.55 | 0.01 | 0.675 |  | 0.63 | 0.89 | 0.02 | 0.466 |
| Valence:Time | 0.75 | 1.5 | 0.03 | 0.229 |  | 0.82 | 0.76 | 0.02 | 0.469 |  | 0.83 | 0.14 | 0.003 | 0.86 |
| Exposure condition:Valence:Time | 0.57 | 0.36 | 0.008 | 0.825 |  | 0.7 | 0.8 | 0.02 | 0.521 |  | 0.62 | 1.77 | 0.04 | 0.14 |
| Game score | 0.16 | 0.04 | <.001 | 0.84 |  | 0.18 | 0.29 | 0.006 | 0.591 |  | 0.17 | 0.41 | 0.008 | 0.525 |
| Feedback duration | 0.16 | 0.72 | 0.01 | 0.401 |  | 0.18 | 0.88 | 0.02 | 0.352 |  | 0.17 | 0.1 | 0.002 | 0.756 |
| Game score:Exposure condition | 0.63 | 0.23 | 0.005 | 0.781 |  | 0.66 | 0.24 | 0.005 | 0.774 |  | 0.6 | 0.78 | 0.02 | 0.457 |
| Feedback duration:Exposure condition | 0.63 | 0.19 | 0.004 | 0.809 |  | 0.66 | 1.89 | 0.04 | 0.16 |  | 0.6 | 1.19 | 0.02 | 0.306 |
| Game score:Valence | 0.46 | 0.99 | 0.02 | 0.326 |  | 0.46 | 0.36 | 0.007 | 0.553 |  | 0.37 | 0.14 | 0.003 | 0.709 |
| Feedback duration:Valence | 0.46 | 0.36 | 0.008 | 0.549 |  | 0.46 | 0.14 | 0.003 | 0.715 |  | 0.37 | 0.21 | 0.004 | 0.647 |
| Game score:Time | 0.57 | 1.04 | 0.02 | 0.357 |  | 0.74 | 1.89 | 0.04 | 0.159 |  | 0.71 | 3.69 | 0.07 | 0.03 |
| Feedback duration:Time | 0.57 | 2.86 | 0.06 | 0.063 |  | 0.74 | 3.51 | 0.07 | 0.036 |  | 0.71 | 4.72 | 0.09 | 0.012 |
| Game score:Exposure condition:Valence | 0.95 | 0.86 | 0.02 | 0.415 |  | 0.81 | 1.61 | 0.03 | 0.205 |  | 0.82 | 2.04 | 0.04 | 0.142 |
| Feedback duration:Exposure condition:Valence | 0.95 | 0.01 | <.001 | 0.989 |  | 0.81 | 0.54 | 0.01 | 0.584 |  | 0.82 | 0.26 | 0.005 | 0.743 |
| Game score:Exposure condition:Time | 0.67 | 0.55 | 0.01 | 0.677 |  | 0.57 | 0.56 | 0.01 | 0.673 |  | 0.63 | 0.86 | 0.02 | 0.482 |
| Feedback duration:Exposure condition:Time | 0.67 | 1.27 | 0.03 | 0.287 |  | 0.57 | 0.61 | 0.01 | 0.636 |  | 0.63 | 0.22 | 0.004 | 0.917 |
| Game score:Valence:Time | 0.75 | 0.45 | <.001 | 0.636 |  | 0.82 | 0.39 | 0.008 | 0.677 |  | 0.83 | 1.51 | 0.03 | 0.227 |
| Feedback duration:Valence:Time | 0.75 | 0.67 | 0.01 | 0.511 |  | 0.82 | 0.29 | 0.006 | 0.745 |  | 0.83 | 1.14 | 0.02 | 0.323 |
| Game score:Exposure condition:Valence:Time | 0.57 | 0.55 | 0.01 | 0.69 |  | 0.7 | 0.58 | 0.01 | 0.666 |  | 0.62 | 0.62 | 0.01 | 0.644 |
| Feedback duration:Exposure condition:Valence:Time | 0.57 | 0.69 | 0.01 | 0.595 |  | 0.7 | 0.8 | 0.02 | 0.518 |  | 0.62 | 0.64 | 0.01 | 0.63 |

**Table S9.** Mean and standard deviation for scores immediately after the game intervention(0h).

|  | **Full** | | |  | **Negative** | | |  | **Positive** | | |
| --- | --- | --- | --- | --- | --- | --- | --- | --- | --- | --- | --- |
|  | **Con** | **Uncon** | **Ctl** |  | **Con** | **Uncon** | **Ctl** |  | **Con** | **Uncon** | **Ctl** |
| **Meteor** | 0.182 | 0.472 | 0.473 |  | 0.083 | 0.281 | 0.342 |  | 0.517 | 0.631 | 0.602 |
|  | (0.633) | (0.571) | (0.489) |  | (0.574) | (0.804) | (0.57) |  | (0.9) | (0.726) | (0.726) |
| **Gist** | 0.256 | 0.431 | 0.46 |  | 0.208 | 0.194 | 0.316 |  | 0.578 | 0.51 | 0.456 |
|  | (0.767) | (0.663) | (0.572) |  | (0.648) | (0.815) | (0.722) |  | (0.889) | (0.642) | (0.783) |
| **Bert** | 0.348 | 0.664 | 0.469 |  | 0.156 | 0.41 | 0.379 |  | 0.631 | 0.646 | 0.46 |
|  | (0.671) | (0.602) | (0.545) |  | (0.657) | (0.808) | (0.606) |  | (0.791) | (0.653) | (0.777) |

Treatment(Treat), Conscious(Con), Unconscious(Uncon), Control(Ctl)

**Table S10.** Mean and standard deviation for scores from 24 hours after the game intervention.

|  | **Full** | | |  | **Negative** | | |  | **Positive** | | |
| --- | --- | --- | --- | --- | --- | --- | --- | --- | --- | --- | --- |
|  | **Con** | **Uncon** | **Ctl** |  | **Con** | **Uncon** | **Ctl** |  | **Con** | **Uncon** | **Ctl** |
| **Meteor** | 0.001 | -0.193 | -0.052 |  | -0.133 | -0.231 | -0.280 |  | 0.136 | 0.079 | 0.165 |
|  | (0.693) | (0.716) | (0.484) |  | (0.912) | (0.736) | (0.577) |  | (0.756) | (0.971) | (0.8) |
| **Gist** | 0.146 | -0.149 | 0.009 |  | -0.033 | -0.172 | -0.139 |  | 0.122 | 0.005 | 0.196 |
|  | (0.746) | (0.638) | (0.594) |  | (0.911) | (0.73) | (0.701) |  | (0.685) | (0.953) | (0.868) |
| **Bert** | 0.06 | -0.193 | 0.043 |  | -0.119 | -0.203 | -0.116 |  | 0.089 | -0.01 | 0.134 |
|  | (0.707) | (0.712) | (0.493) |  | (0.923) | (0.749) | (0.761) |  | (0.667) | (0.871) | (0.761) |

**Table S11.** Mean and standard deviation for scores from 72 hours after the game intervention.

|  | **Full** | | |  | **Negative** | | |  | **Positive** | | |
| --- | --- | --- | --- | --- | --- | --- | --- | --- | --- | --- | --- |
|  | **Con** | **Uncon** | **Ctl** |  | **Con** | **Uncon** | **Ctl** |  | **Con** | **Uncon** | **Ctl** |
| **Meteor** | -0.547 | -0.450 | -0.603 |  | -0.475 | -0.498 | -0.602 |  | -0.335 | -0.27 | -0.442 |
|  | (0.629) | (0.758) | (0.474) |  | (0.691) | (0.944) | (0.685) |  | (0.944) | (0.667) | (0.636) |
| **Gist** | -0.471 | -0.442 | -0.57 |  | -0.396 | -0.504 | -0.64 |  | -0.442 | -0.306 | -0.336 |
|  | (0.716) | (0.828) | (0.542) |  | (0.62) | (0.933) | (0.78) |  | (0.824) | (0.7) | (0.873) |
| **Bert** | -0.507 | -0.445 | -0.555 |  | -0.456 | -0.572 | -0.641 |  | -0.426 | -0.269 | -0.368 |
|  | (0.76) | (0.849) | (0.556) |  | (0.719) | (0.971) | (0.771) |  | (0.946) | (0.727) | (0.749) |

**Table S12.** The difference in the Power Spectrum Density between “conscious and unconscious” (conscious – unconscious, Wilcoxon two-sided test, n=1440).

| Channel | Frequency band | Median(PSD) | Statistic | P-value |  |
| --- | --- | --- | --- | --- | --- |
| AF3 | Delta | 0.134 | 516662 | 0.894 |  |
| AF3 | Theta | -0.206 | 485261 | 0.034 | * |
| AF3 | Alpha | -0.075 | 488098 | 0.052 |  |
| AF3 | Beta | 0.014 | 512924 | 0.712 |  |
| AF3 | Gamma | 0.141 | 495258 | 0.136 |  |
| F7 | Delta | -0.026 | 510959 | 0.621 |  |
| F7 | Theta | 0.002 | 502316 | 0.297 |  |
| F7 | Alpha | -0.225 | 485349 | 0.034 | * |
| F7 | Beta | 0.089 | 509533 | 0.559 |  |
| F7 | Gamma | 0.017 | 506590 | 0.441 |  |
| F3 | Delta | 0.301 | 502744 | 0.310 |  |
| F3 | Theta | -0.233 | 492573 | 0.097 |  |
| F3 | Alpha | -0.042 | 496267 | 0.154 |  |
| F3 | Beta | -0.052 | 508619 | 0.521 |  |
| F3 | Gamma | 0.025 | 501523 | 0.275 |  |
| FC5 | Delta | 0.534 | 491622 | 0.086 |  |
| FC5 | Theta | -0.039 | 494721 | 0.128 |  |
| FC5 | Alpha | -0.075 | 488539 | 0.056 |  |
| FC5 | Beta | -0.139 | 506699 | 0.445 |  |
| FC5 | Gamma | -0.051 | 510597 | 0.605 |  |
| T7 | Delta | 0.610 | 512473 | 0.690 |  |
| T7 | Theta | -0.154 | 490564 | 0.074 |  |
| T7 | Alpha | -0.270 | 478162 | 0.010 | * |
| T7 | Beta | -0.024 | 505426 | 0.398 |  |
| T7 | Gamma | 0.011 | 516048 | 0.864 |  |
| P7 | Delta | 0.289 | 504690 | 0.373 |  |
| P7 | Theta | -0.233 | 493247 | 0.106 |  |
| P7 | Alpha | -0.037 | 508666 | 0.522 |  |
| P7 | Beta | -0.269 | 486117 | 0.039 | * |
| P7 | Gamma | 0.016 | 514796 | 0.802 |  |
| O1 | Delta | 1.096 | 489689 | 0.065 |  |
| O1 | Theta | -0.542 | 481842 | 0.019 | * |
| O1 | Alpha | -0.219 | 489746 | 0.066 |  |
| O1 | Beta | -0.146 | 485960 | 0.038 | * |
| O1 | Gamma | -0.173 | 474174 | 0.005 | ** |
| O2 | Delta | -0.077 | 516638 | 0.893 |  |
| O2 | Theta | -0.394 | 477784 | 0.009 | ** |
| O2 | Alpha | -0.309 | 481050 | 0.017 | * |
| O2 | Beta | -0.384 | 480313 | 0.015 | * |
| O2 | Gamma | -0.103 | 496482 | 0.158 |  |
| P8 | Delta | 0.636 | 502690 | 0.309 |  |
| P8 | Theta | -0.399 | 484275 | 0.029 | * |
| P8 | Alpha | -0.218 | 478851 | 0.011 | * |
| P8 | Beta | -0.111 | 495200 | 0.135 |  |
| P8 | Gamma | -0.114 | 508356 | 0.510 |  |
| T8 | Delta | 0.063 | 514027 | 0.764 |  |
| T8 | Theta | -0.331 | 492647 | 0.098 |  |
| T8 | Alpha | -0.120 | 497297 | 0.174 |  |
| T8 | Beta | -0.183 | 498278 | 0.194 |  |
| T8 | Gamma | 0.043 | 506567 | 0.440 |  |
| FC6 | Delta | -0.138 | 514877 | 0.806 |  |
| FC6 | Theta | -0.180 | 493878 | 0.115 |  |
| FC6 | Alpha | -0.064 | 495701 | 0.144 |  |
| FC6 | Beta | -0.238 | 488599 | 0.056 |  |
| FC6 | Gamma | -0.206 | 472072 | 0.003 | ** |
| F4 | Delta | 0.050 | 518138 | 0.969 |  |
| F4 | Theta | -0.153 | 502420 | 0.301 |  |
| F4 | Alpha | -0.172 | 486948 | 0.044 | * |
| F4 | Beta | -0.150 | 482759 | 0.023 | * |
| F4 | Gamma | 0.019 | 517101 | 0.916 |  |
| F8 | Delta | 0.969 | 494407 | 0.123 |  |
| F8 | Theta | -0.053 | 517799 | 0.951 |  |
| F8 | Alpha | -0.132 | 495405 | 0.139 |  |
| F8 | Beta | -0.020 | 518052 | 0.964 |  |
| F8 | Gamma | -0.087 | 496706 | 0.162 |  |
| AF4 | Delta | 0.120 | 517869 | 0.955 |  |
| AF4 | Theta | -0.143 | 504367 | 0.362 |  |
| AF4 | Alpha | -0.055 | 498349 | 0.196 |  |
| AF4 | Beta | -0.164 | 499021 | 0.211 |  |
| AF4 | Gamma | -0.031 | 509295 | 0.549 |  |

* p<0.05 ** p<0.01 *** p<0.001

**Table S13.** The difference in the Power Spectrum Density between “positive and negative” (negative – positive, Wilcoxon two-sided test, n=1440).

| Channel | Frequency band | Median(PSD) | Statistic | P-value |  |
| --- | --- | --- | --- | --- | --- |
| AF3 | Delta | -0.987 | 499105 | 0.213 |  |
| AF3 | Theta | -0.243 | 508446 | 0.513 |  |
| AF3 | Alpha | 0.036 | 511028 | 0.624 |  |
| AF3 | Beta | 0.094 | 484453 | 0.030 | * |
| AF3 | Gamma | -0.023 | 511737 | 0.656 |  |
| F7 | Delta | -2.539 | 472413 | 0.003 | ** |
| F7 | Theta | 0.112 | 516963 | 0.909 |  |
| F7 | Alpha | 0.1 | 489819 | 0.067 |  |
| F7 | Beta | 0.253 | 465527 | <0.001 | *** |
| F7 | Gamma | 0.231 | 449729 | <0.001 | *** |
| F3 | Delta | -0.288 | 502431 | 0.301 |  |
| F3 | Theta | -0.131 | 506888 | 0.452 |  |
| F3 | Alpha | 0.201 | 495610 | 0.142 |  |
| F3 | Beta | 0.197 | 469853 | 0.002 | ** |
| F3 | Gamma | 0.121 | 477806 | 0.009 | ** |
| FC5 | Delta | -0.766 | 488147 | 0.052 |  |
| FC5 | Theta | -0.142 | 492968 | 0.102 |  |
| FC5 | Alpha | 0.178 | 478598 | 0.011 | * |
| FC5 | Beta | 0.313 | 443573 | <0.001 | *** |
| FC5 | Gamma | 0.312 | 429448 | <0.001 | *** |
| T7 | Delta | -0.676 | 512427 | 0.688 |  |
| T7 | Theta | -0.202 | 497320 | 0.174 |  |
| T7 | Alpha | 0.211 | 485092 | 0.033 | * |
| T7 | Beta | 0.24 | 474094 | 0.005 | ** |
| T7 | Gamma | -0.051 | 503847 | 0.345 |  |
| P7 | Delta | 0.351 | 518147 | 0.969 |  |
| P7 | Theta | 0.013 | 504522 | 0.367 |  |
| P7 | Alpha | 0.479 | 444328 | <0.001 | *** |
| P7 | Beta | 0.189 | 477750 | 0.009 | ** |
| P7 | Gamma | 0.081 | 500270 | 0.241 |  |
| O1 | Delta | -0.612 | 500554 | 0.249 |  |
| O1 | Theta | -0.029 | 509282 | 0.548 |  |
| O1 | Alpha | 0.416 | 480625 | 0.016 | * |
| O1 | Beta | 0.214 | 475562 | 0.006 | ** |
| O1 | Gamma | 0.22 | 466479 | <0.001 | *** |
| O2 | Delta | -0.349 | 517738 | 0.948 |  |
| O2 | Theta | 0.17 | 502470 | 0.302 |  |
| O2 | Alpha | 0.211 | 484258 | 0.029 | * |
| O2 | Beta | 0.691 | 425211 | <0.001 | *** |
| O2 | Gamma | 0.373 | 432249 | <0.001 | *** |
| P8 | Delta | -0.111 | 513973 | 0.762 |  |
| P8 | Theta | 0.168 | 513659 | 0.747 |  |
| P8 | Alpha | -0.018 | 514335 | 0.779 |  |
| P8 | Beta | 0.399 | 456855 | <0.001 | *** |
| P8 | Gamma | 0.163 | 469340 | 0.002 | ** |
| T8 | Delta | -1.682 | 449803 | <0.001 | *** |
| T8 | Theta | -0.32 | 477138 | 0.008 | ** |
| T8 | Alpha | -0.023 | 512021 | 0.669 |  |
| T8 | Beta | 0.23 | 468773 | 0.002 | ** |
| T8 | Gamma | 0.01 | 490279 | 0.071 |  |
| FC6 | Delta | -0.058 | 509490 | 0.557 |  |
| FC6 | Theta | 0.052 | 502179 | 0.293 |  |
| FC6 | Alpha | 0.063 | 487531 | 0.048 | * |
| FC6 | Beta | 0.757 | 419932 | <0.001 | *** |
| FC6 | Gamma | 0.268 | 445790 | <0.001 | *** |
| F4 | Delta | 0.033 | 514981 | 0.811 |  |
| F4 | Theta | -0.068 | 498581 | 0.201 |  |
| F4 | Alpha | 0.296 | 474375 | 0.005 | ** |
| F4 | Beta | 0.554 | 391824 | <0.001 | *** |
| F4 | Gamma | 0.208 | 428117 | <0.001 | *** |
| F8 | Delta | -1.054 | 487769 | 0.049 | * |
| F8 | Theta | 0.057 | 514166 | 0.771 |  |
| F8 | Alpha | 0.071 | 502948 | 0.316 |  |
| F8 | Beta | 0.662 | 447444 | <0.001 | *** |
| F8 | Gamma | 0.117 | 483012 | 0.024 | * |
| AF4 | Delta | -0.871 | 501251 | 0.267 |  |
| AF4 | Theta | -0.012 | 496180 | 0.153 |  |
| AF4 | Alpha | -0.114 | 504835 | 0.378 |  |
| AF4 | Beta | 0.264 | 489378 | 0.063 |  |
| AF4 | Gamma | 0.071 | 502752 | 0.310 |  |

**Additional Data S1.** Some examples of Gist scores compared to METEOR score.

| **Subject number** | **Image number** | **Participant’s utterance** | **Gist score** | **METEOR score** |
| --- | --- | --- | --- | --- |
| 46 | 2 | It was a picture of a woman in a bathing suit standing on a beach with a parasol set up and playing under it, and a guy lying on top of it with a little bit of beach fur and stuff. | 0.000 | 0.120 |
| 9 | 2 | Men and women are playing on the beach. | 1.000 | 0.053 |
| 46 | 2 | It was a picture of a woman in a bathing suit standing on a beach with a parasol set up and playing under it, and a guy lying on top of it with a little bit of beach fur and stuff. | 0.333 | 0.120 |
| 29 | 2 | You’re on the beach with a parasol and it’s sunny. | 1.000 | 0.064 |
| 46 | 2 | It was a picture of a woman in a bathing suit standing on a beach with a parasol set up and playing under it, and a guy lying on top of it with a little bit of beach fur and stuff. | 0.333 | 0.120 |
| 33 | 2 | It was that ocean with people playing in it. | 1.000 | 0.032 |
| 23 | 6 | This was on the TV, and this was on the left side of the room, and the family was watching a sporting event on the TV, like a soccer game or something, and the room was kind of dark, and it was just the TV on. | 0.667 | 0.202 |
| 15 | 6 | It was a scene where people were cheering while watching a game on TV. | 1.000 | 0.136 |
| 23 | 6 | This was on the TV, and this was on the left side of the room, and the family was watching a sporting event on the TV, like a soccer game or something, and the room was kind of dark, and it was just the TV on. | 0.667 | 0.202 |
| 46 | 6 | This is not a soccer game and I don’t remember this. | 1.000 | 0.070 |
| 23 | 6 | This was on the TV, and this was on the left side of the room, and the family was watching a sporting event on the TV, like a soccer game or something, and the room was kind of dark, and it was just the TV on. | 0.667 | 0.202 |
| 65 | 6 | I think it was three guys watching the game and celebrating, and there was a couch. | 1.000 | 0.135 |
| 38 | 10 | I think it was one of those birthday or something like that where there’s a girl in the middle and there’s a guy next to her and there’s like three of them and they’re all happy. | 0.000 | 0.101 |
| 11 | 10 | people laughing. | 0.667 | 0.007 |
| 38 | 10 | I think it was one of those birthday or something like that where there’s a girl in the middle and there’s a guy next to her and there’s like three of them and they’re all happy. | 0.000 | 0.101 |
| 60 | 10 | It was a routine scene. | 1.000 | 0.014 |
| 13 | 12 | It was a scene where another man was covering one woman’s eyes like she was giving her a surprise gift, and she was about to hand over a gift box in a red box around her. | 0.667 | 0.159 |
| 70 | 12 | People drinking and having fun, | 1.000 | 0.021 |
| 45 | 14 | I think this was a music classroom at a music school or something, and there’s a chalkboard in the back with music or something, and a kid in the front with a guitar, and there’s a teacher and a bunch of kids. | 0.000 | 0.187 |
| 9 | 14 | It’s music class, I have children, and I have a teacher. | 0.667 | 0.128 |
| 45 | 14 | I think this was a music classroom at a music school or something, and there’s a chalkboard in the back with music or something, and a kid in the front with a guitar, and there’s a teacher and a bunch of kids. | 0.000 | 0.187 |
| 21 | 14 | acting | 0.333 | 0.009 |
| 61 | 17 | I remember a scene where a group of children, several of them wearing blue headbands, seemed to be boarding a yellow school bus. There was a woman standing at the front of the bus and the children looked like they were going to kindergarten of some sort. | 0.667 | 0.259 |
| 10 | 17 | Young students are on the school bus, and a female teacher is greeting the student. | 1.000 | 0.095 |
| 61 | 17 | I remember a scene where a group of children, several of them wearing blue headbands, seemed to be boarding a yellow school bus. There was a woman standing at the front of the bus and the children looked like they were going to kindergarten of some sort. | 0.667 | 0.259 |
| 64 | 17 | It was a photo of children boarding a vehicle that appeared to be a school bus. | 0.750 | 0.095 |
| 61 | 17 | I remember a scene where a group of children, several of them wearing blue headbands, seemed to be boarding a yellow school bus. There was a woman standing at the front of the bus and the children looked like they were going to kindergarten of some sort. | 0.667 | 0.259 |
| 143 | 17 | It looks like a school bus, with teachers on top of it and going to school. | 1.000 | 0.133 |
| 69 | 17 | In front of an American yellow school bus, a teacher is lined up on the right and students on the left. The first student and teacher are making a fist bump, and the third student is wearing a blue headband. | 0.000 | 0.307 |
| 25 | 17 | A girl is wearing a blue colored headband. | 0.250 | 0.043 |
| 66 | 19 | A group of men surround a group of wounded people. In one corner of the group of wounded people, people are holding guns and trying to shoot the people beyond them, there are flyers or something on the wall, and the whole thing is gray. | 0.000 | 0.219 |
| 8 | 19 | Police and people are fighting over this cat flyer. | 0.750 | 0.017 |
| 66 | 19 | A group of men surround a group of wounded people. In one corner of the group of wounded people, people are holding guns and trying to shoot the people beyond them, there are flyers or something on the wall, and the whole thing is gray. | 0.000 | 0.219 |
| 10 | 19 | Some rioters were rioting, and soldiers were waiting behind the walls to put it down. | 0.250 | 0.090 |
| 66 | 19 | A group of men surround a group of wounded people. In one corner of the group of wounded people, people are holding guns and trying to shoot the people beyond them, there are flyers or something on the wall, and the whole thing is gray. | 0.000 | 0.219 |
| 11 | 19 | I remember being in a war. I remember one person pointing a gun at him. | 0.250 | 0.059 |
| 66 | 19 | A group of men surround a group of wounded people. In one corner of the group of wounded people, people are holding guns and trying to shoot the people beyond them, there are flyers or something on the wall, and the whole thing is gray. | 0.000 | 0.219 |
| 15 | 19 | It was a scene where people were fighting in an alley or something, | 0.750 | 0.034 |
| 52 | 20 | Now the young black kids were now holding out their hands for something, there were several of them, and they were holding out their hands and they were like begging for something, or trying to stop something. | 0.000 | 0.135 |
| 10 | 20 | Several black children are bursting into tears. | 0.667 | 0.071 |
| 36 | 24 | The guy in white was in the middle, and then there was another guy on the far left, and then there was a woman hanging between them, and I think she passed out and was hanging on. | 0.333 | 0.075 |
| 63 | 24 | A doctor was transporting a patient. | 0.500 | 0.029 |
| 23 | 28 | This guy in the pants was pointing a gun and there were two people in the direction that he was pointing and they both had their hands up in the air, surrendering or something. | 0.667 | 0.132 |
| 144 | 28 | Two men were fighting | 1.000 | 0.035 |
| 30 | 28 | This is a picture of three men, three middle-aged men, one of whom is being slightly dragged, and this is a picture of a cargo truck that has completely tipped over and is lying on the road. | 0.333 | 0.105 |
| 144 | 28 | Two men were fighting | 1.000 | 0.035 |
| 51 | 34 | There’s a car that’s flipped over and it’s a black car, and it’s a black car that’s flipped over and it’s just kind of upside down, and there’s a bunch of people there trying to figure it out. | 0.000 | 0.091 |
| 21 | 34 | The car turned upside down. There were several rescuers. | 1.000 | 0.024 |
| 23 | 35 | It was a situation where there was a white car and a white yacht on a cliff and the car had fallen off the cliff and the people’s yacht was on the cliff and the people were trying to pull the car up. | 0.750 | 0.179 |
| 145 | 35 | The car flipped over and I was about to fall out. | 1.000 | 0.040 |
| 66 | 35 | It looks like something like a boat hit a car on a cliff on a city street and the car is about to fall off the cliff. There is a big tree on the right and several people are standing on the cliff looking down. | 0.500 | 0.158 |
| 64 | 35 | A car is hanging precariously as if it will fall from a cliff-like space. | 1.000 | 0.060 |
| 66 | 35 | It looks like something like a boat hit a car on a cliff on a city street and the car is about to fall off the cliff. There is a big tree on the right and several people are standing on the cliff looking down. | 0.500 | 0.158 |
| 145 | 35 | The car flipped over and I was about to fall out. | 1.000 | 0.040 |
| 52 | 36 | This is now in a hotel room or something, and there’s a guy with tights on and glasses inside, and he’s got a gun or something, and he’s now sitting on the bed, and there’s not just that gun, there’s multiple guns in front of him. | 0.750 | 0.174 |
| 53 | 36 | I can’t think of the exact image, but it reminds me of a hospital. | 1.000 | 0.033 |
| 52 | 36 | This is now in a hotel room or something, and there’s a guy with tights on and glasses inside, and he’s got a gun or something, and he’s now sitting on the bed, and there’s not just that gun, there’s multiple guns in front of him. | 0.750 | 0.174 |
| 64 | 36 | It was a poor quality photo of a man with a gun on his bed. | 1.000 | 0.061 |
