# Supplementary material for: Shadow-Induced Forgetting in a Game-Based Paradigm on Nonclinical Adults and Its Effects on Consciousness, Emotional Valence, and Temporal Dynamics: Crossover Study
Source: JMIR Serious Games. 2025 Dec 30;13:e76946. doi: 10.2196/76946 (PMC12753131; doi:10.2196/76946)
Supplement: Multimedia Appendix 2 [file games-v13-e76946-s002.pdf]

연구대상자용 설명문

- **연구 제목** : PTSD 환자 증상 완화를 위한 '특이적 기억 약화' 효과를 가진 비침습적 게임 치료제의 유효성 검증
- **연구 책임자** : 박유랑

본 연구는 향후 PTSD 환자 치료를 위한 디지털 치료제 개발을 위하여, '특이적 기억 약화' 효과를 가진 비침습적 게임 치료의 유효성 검증을 위한 연구입니다.

이 설명문은 해당 연구에 대한 귀하의 충분한 이해를 돕기 위하여 작성된 것으로, 설명서와 동의서를 신중하게 읽어보시고 참여 결정 여부를 결정해주시기 바랍니다.

본 연구의 참여 여부를 결정하기에 앞서, 본 연구의 목적과 절차, 어떤 사람들이 이 연구에 참여하는지, 연구 참여를 통해 기대할 수 있는 이익과 위험하거나 불편한 점은 무엇인지 등에 대하여 귀하께서 충분히 설명을 듣고 이해하는 것이 중요합니다. 서면상 이해가 어려운 부분에 대해서는 이 연구를 수행하는 연구원에게 설명해 줄 것을 요구하실 수 있습니다. 귀하의 자발적 의사로 연구에 참여 여부를 결정할 수 있으며, 필요하다면 가족이나 친구들과 의논해 보실 수 있습니다. 참여하지 않기로 결정하더라도 귀하에게 아무런 불이익도 없을 것입니다

귀하의 서명은 귀하가 본 연구에 대해 그리고 위험성에 대해 설명을 들었음을 의미하며, 이 문서에 대한 귀하의 서명은 귀하께서 자신(또는 법정대리인)이 본 연구에 참가를 원한다는 것을 의미합니다.

1. 이번 연구가 이루어지는 배경과 목적

외상 후 스트레스 장애(Post Traumatic Stress Disorder, PTSD)는 심각한 충격을 불러 일으키는 외상 사건(Traumatic event)을 직•간접적으로 경험함으로써 나타날 수 있는 심리적 장애입니다. 충격적인 사건의 재경험과 이와 관련된 상황 및 자극으로 인한 해리 현상이나 공황발작, 환청 및 지각 이상을 경험할 수 있으며, 연관 증상으로 공격적 성향, 충동조절 장애, 우울증, 약물 남용 또는 집중력 및 기억력 저하 등의 인지기능 문제가 나타날 수 있습니다.

전 세계적으로 PTSD 환자의 수는 급속도로 증가하는 추세이며, 2021년 기준 국내 PTSD 환자 수는 12,879명으로, 이는 2017년에 비해 63% 증가한 수치입니다. 또한 일생 동안 한 번 이상 트라우마를 경험하는 한국 청장년은 89.9%에 달할 정도로 PTSD는 더 이상 소수의 질병이 아닙니다.

하지만 이러한 PTSD 치료를 위한 현재의 치료 방식은 트라우마-중심 치료법의 경우 환자에게 트라우마를 일으키는 외상 사건을 재경험하게 하는 매우 '침습적'인 방식이며, 명상 또는 관계성 훈련 등의 간접적 치료 방법의 경우 그 치료 효능이 불확실합니다.

따라서 본 연구는 기존의 PTSD 치료가 갖는 '높은 중도 이탈률'과 '낮은 효능'을 야기하는 요인을 극복하는 PTSD 치료제 개발을 위하여, '망각적 그늘(amnesic shadow)'과 '무의식 자극(unconscious reactivation)' 이라는 이론적 기반을 갖는, '특이적 기억 약화' 효과를 가진 '비침습적' 게임 개발을 목표로 합니다.

2. 연구에 참여하는 대상자의 수, 기간과 장소

본 연구는 사전 설문을 통하여 선별한 건강 상의 특이사항이 없으며, 한국어를 유창하게 구사 가능한, 교정 시력 0.5 이상, 만 19~29세 까지의 서울 소재 일반 대학생 150명을 대상으로 이루어집니다. 연구 대상자는 최초 방문 시 헤드셋 형태의 EEG(Electro Encephalo Graphy) 측정 기기를 착용한 상태에서 본 연구를 위해 설계된 게임인 '화살표 피하기 게임'을 연구원의 관찰 하에 약 30~40분 동안 진행하게 됩니다. 연구는 '서울시 서대문구

신촌역로 29 연세대학교 캠퍼스타운 에스큐브 2호점'에서 대면으로 수행되며, 최초 방문 이후에 총 2회의 추가 방문이 요구됩니다. 2회차와 3회차 방문은 최초 방문 이후로부터 각각 24시간과 72시간 뒤에 이루어지며, 이때 연구 대상자는 간단한 질문에 대해 구술 답변을 수행하게 되며, 1회차 방문 시 소요시간은 약 40분, 2회차와 3회차 방문 시 소요시간은 약 20분 내외입니다.

3. 연구의 절차 및 방법

이 연구에서 이루어지는 절차는 다음과 같습니다.

(1) 연구 참여를 희망하는 연구 대상자에 한하여 사전 설문 조사를 통해 귀하의 성별, 연령대, 재학 중인 학교 등 기본정보를 확인합니다. 설문 조사결과에 따라 연구 참여 가능여부가 결정됩니다.

(2) 귀하의 연구 참여가 결정되셨다면, 게임 진행 전 연구 참여 동의 과정을 진행합니다. 동의 전 연구에 관하여 궁금한 사항이 있다면 연구자에게 질문하여 주십시오.

(3) 동의 완료 후 게임(화살표 피하기 게임)은 다음의 절차로 진행 됩니다. 1) '학습 단계'에서는 총 36쌍의 이미지가 제시되며, 제한된 시간 동안 제시된 이미지에 대한 암기가 요구됩니다. 약 10~15분 간 진행됩니다. 2) '게임 단계'에서는 본 연구를 위하여 설계된 '화살표 피하기 게임'을 진행합니다. 약 5분 간 진행됩니다. 3) '테스트 단계'에서는 '학습 단계'에서 학습한 이미지 쌍에 대한 암기 수준을 평가합니다. 순서로 진행합니다. 약 15~20분 간 진행됩니다. 따라서 해당 게임 절차는 총 30~40분 가량 소요될 수 있습니다. 대면으로 이루어지는 본 실험은 녹화되며 연구의 목적으로만 사용될 예정입니다.

본 과정에서 헤드셋 형태의 뇌파 측정 기기를 착용하게 됩니다. 해당 기기를 머리에 착용하는 과정에서 두피의 국소부위에 소량의 젤을 바를 수 있습니다. 젤의 용도는 두피의 각질 제거를 위함이며, 인체와 피부에는 아주 무해합니다. 기기를 머리에 부착하는 과정에서 머리카락이 조금 헝클어질 수 있으며, 생리식염수에 적셔진 스티로폼이 두피에 직접 닿을 수 있습니다. 생리식염수 및 스티로폼은 인체와 피부에 아주 무해합니다.

4. 연구에 참여 하여 기대할 수 있는 이익

귀하가 본 연구에 참여함으로 인해 발생하는 직접적인 이익은 없으나, 본 연구의 결과를 통해 '특이적 기억 약화' 효과를 가진 게임에 대한 근거를 제시함으로써 기존의 PTSD 치료의 효능을 낮추고, 중도 이탈률을 높이는 '침습적'이라는 한계를 극복한 게임 기반의 PTSD 디지털 치료제 개발에 기여할 수 있을 것으로 기대합니다.

5. 연구 참여에 대한 사례

총 3회의 연구 중 2회 연구에 참여하시는 분께 5 만원의 사례비가 지급되며, 3회 모두 참여하시는 분께 7만원의 사례비가 지급됩니다.

6. 연구에 참여하여 예상되는 위험 및 불편

연구 과정 중 '학습 단계'에서 제시되는 이미지 중 정서적/감성적 불편함을 야기할 수 있는 장면이 포함되어 있을 수 있습니다. 이를 최소화하기 위해 모든 장면 이미지에 대한 폭력성, 자극성 수치를 일정 수준 초과하지 않도록 맞추었습니다. 연구 과정 동안 혹시 모를 상황에 대비하여 상시 연구원이 함께할 것이며, 혹시라도 연구 과정 중의 불편함으로 인해 참여 중단을 희망하신다면 언제든지 중도 포기가 가능합니다.

7. 연구 참여 도중 중도탈락

연구 시작 전 귀하에게 해당 연구의 목적과 방법 등에 대한 충분한 정보가 제공될 것입니다. 만일, 연구 진행 이전 또는 도중에 연구 참여 의사가 변경될 경우, 연구 담당자에게 연락을 주시면 언제든 연구 참여를 포기할 수 있습니다.

8. 정보 수집 및 제공

본 동의서에 서명함으로써 귀하는 연구진이 귀하의 개인(민감)정보를 수집하고 사용하는데 동의하게 됩니다. (※ 자세한 사항은 아래 내용을 참조하여 주십시오.)

1) 개인정보의 수집 · 이용 목적

- 기준을 충족하는 연구 대상자의 선별 및 향후 비침습적 PTSD 디지털 치료제

개발에서 해당 게임의 기억 약화 효과에 대한 근거를 제시하기 위한 통계자료로써 개인정보를 사용합니다.

2) 수집하려는 개인(민감)정보의 항목

개인정보	성명, 성별, 연령, 연락처, 재학 정보
민감정보	교정 시력, 뇌파 정보(EEG 측정치)

3) 개인(민감)정보의 보유 및 이용 기간

- 귀하의 개인(민감)정보는 연구를 위해 2 년간 사용되며 수집된 개인정보는 개인정보보호법에 따라 적절히 관리 됩니다.

4) 동의를 거부할 권리 및 동의 거부에 따른 불이익

불이익의 내용

- 귀하는 위 개인(민감)정보 수집 및 이용, 제공에 대한 수락 여부를 자유롭게 결정할 수 있습니다. 귀하의 자녀의 개인(민감)정보 수집 및 이용, 제공에 수락하지 않는 경우에도 귀하와 귀하의 아동에 그 어떠한 불이익도 발생하지 않습니다. 그러나 동의를 거부할 경우 본 연구 참여에 제한을 받을 수 있습니다.

5) 연구에서 수집된 대상 개인정보의 타인제공 여부와 만일 제공한다면 개인 식별정보를 포함하는지 여부

- 이 연구에서 수집하는 위의 개인(민감)정보는 본 연구 목적 이외의 목적으로 사용되거나 타인에게 제공되지 않습니다. 기타 논문 투고 및 연구 공개 과정에서 해당 연구의 데이터 활용 시에는 개인 식별 정보를 제외한 통계자료만을 활용합니다.

연세대학교 YONSEI UNIVERSITY

9. 개인정보 및 기록에 대한 비밀보장

귀하가 이 연구에 참여하는 동안에 수집되는 개인정보는 비밀로 보장될 것이며, 연구의 결과가 보고서로 작성되거나 출판, 또는 발표되는 경우에도 귀하의 신원을 파악할 수 있는 기록은 비밀 상태로 유지됩니다.

이 연구를 진행하는 자, 모니터/점검하는 자, 연구심의위원회 등은 귀하의 비밀 보장을 침해하지 않고 관련 규정이 정하는 범위 안에서 연구의 실시 절차와 자료의 신뢰성을 검증하기 위해 귀하의 병력청취 기록을 직접 열람할 수 있습니다. 귀하는 본 동의서 서식에 서명함으로써 귀하의 이러한 자료의 직접 열람을 허용하시게 됩니다.

수집한 자료는 유출되지 않도록 보관, 관리되며, 개인정보의 보유 및 이용 기간이 만료된 시점, 저장 매체, 인쇄물 등 어떠한 형태의 자료도 모두 지체없이 파기 합니다. 본 연구의 자료는 통계법 제 33 조(비밀보호)에 의거하여 연구자 외 열람, 유출, 누출이 불가능하도록 데이터베이스에 관련 연구자만 접속할 수 있도록 암호화하여 보관합니다. 자료의 보관은 연구가 종결된 후 2 년 보관 후 폐기하도록 합니다. 자료 관리 과정에서 문제점이 발견되는 경우 신속한 조치를 통해 문제 상황을 해결하겠습니다.

10. 참여/철회의 자발성

귀하는 언제든지 본 연구 참여에 대해 동의를 철회 할 수 있으며, 이 경우, 연구 참여는 종료되고 연구진은 귀하의 연구와 관련하여 추가적인 정보를 수집하지 않을 것이며, 이전까지 수집된 정보는 즉각 폐기되며, 향후 연구에서 모두 제외됩니다. 연구에 참여하지 않거나 중도에 그만 두기로 결정하더라도, 귀하께 어떠한 불이익도 발생하지 않을 것이며 귀하께서 원래 받을 수 있는 이익에 어떠한 손실 또한 없을 것입니다. 귀하께서 연구에 계속해서 참여할 지를 결정하는 데에 영향을 주는 새로운 정보가 수집될 경우, 연구자는 이 정보를 귀하께 적시에 알려드릴 것입니다. 또한 모든 연구 분석에서 사용되는 자료는 귀하의 동의가 완료된 자료만을 활용합니다.

11. 연구 문의

본 연구에 대해 질문이 있거나 연구 중간에 문제가 생길 시 다음 연구 담당자에게 언제든지 연락하여 주십시오.

연구자 성명 : 박유랑

연구자 주소 : 서울특별시 서대문구 연세로 50-1 (03722, 02-2228-2493)

실무 연구자 전화번호 : 010-5218-3284

대상자로서 귀하의 권리에 대하여 질문이 있는 경우에는 연구자에게 말씀하시거나 다음의 번호로 문의하실 수 있습니다.

세브란스병원 연구심의위원회(IRB) ☎ 02-2228-0430~4

세브란스병원 임상연구보호센터(HPC) ☎ 02-2228-0450~4

연세대학교
YONSEI UNIVERSITY

동 의 서

연구제목: PTSD 환자 증상 완화를 위한 '특이적 기억 약화' 효과를 가진 비침습적 게임 치료제의 유효성 검증

1. 나는 본 연구의 설명문을 읽었으며 담당 연구원과 이에 대하여 의논하였습니다.

2. 나는 위험과 이득에 관하여 들었으며 나의 질문에 만족할 만한 답변을 얻었습니다.

3. 나는 이 연구에 참여하는 것에 대하여 자발적으로 동의합니다.

4. 나는 이 연구에서 얻어진 나에 대한 정보를 현행 법률과 기관생명윤리위원회 규정이 허용하는 범위 내에서 연구자가 수집하고 처리하는데 동의합니다.

5. 나는 담당 연구자나 위임 받은 대리인이 연구를 진행하거나 결과 관리를 하는 경우와 연구기관, 연구비지원기관 및 보건복지부 지정 공용기관생명윤리위원회가 실태 조사를 하는 경우에는 비밀로 유지되는 나의 개인 신상 정보를 직접적으로 열람하는 것에 동의합니다.

6. 나는 언제라도 이 연구의 참여를 철회할 수 있고 이러한 결정이 나에게 어떠한 해도 되지 않을 것이라는 것을 압니다.

7. 나의 서명은 이 동의서의 사본을 받았다는 것을 뜻하며 연구 참여가 끝날 때까지 사본을 보관하겠습니다.

연구대상자	성명:	서명:	서명일:
동의취득자	성명:	서명:	서명일:

Participant Information Sheet

- **Study Title**: Efficacy Verification of a Non-Invasive Game Therapeutic Intervention With a "Specific Memory Weakening" Effect for Alleviating PTSD Symptoms
- **Principal Investigator:** Yu-Rang Park

This study aims to verify the efficacy of a non-invasive game-based therapeutic intervention designed to produce a "specific memory weakening" effect, which may contribute to future digital therapeutics for patients with PTSD.
This information sheet is provided to help you fully understand the study. Please read both the information sheet and consent form carefully before deciding whether to participate.

Before deciding to participate, it is important that you fully understand the purpose and procedures of this study, the characteristics of individuals eligible to participate, and the potential benefits, risks, or discomforts associated with participating.
If you have difficulty understanding the written information, you may request further explanation from the researcher.
Participation is entirely voluntary, and you may consult with your family or friends if needed. Choosing not to participate will not result in any disadvantage.

Your signature indicates that you have received information about this study and its risks, and that you voluntarily agree to participate.

1. Background and Purpose of the Study

Post-Traumatic Stress Disorder (PTSD) is a psychological condition that can occur after direct or indirect exposure to a traumatic event. Individuals may experience re-experiencing of the traumatic event, dissociation, panic attacks, hallucinations, or perceptual disturbances. Associated symptoms may include aggressiveness, impulse-control difficulties, depression, substance misuse, and cognitive impairments such as reduced concentration and memory problems.

Globally, the number of PTSD patients continues to rise. As of 2021, 12,879 individuals in Korea were diagnosed with PTSD—a 63% increase from 2017. Moreover, approximately 89.9% of young adults in Korea experience at least one traumatic event during their lifetime, indicating that PTSD is no longer a rare condition.

However, current PTSD treatments have significant limitations:
- Trauma-focused therapies require patients to revisit traumatic experiences, making them highly invasive.
- Indirect interventions (such as meditation or relational training) have uncertain efficacy.

To address the high dropout rates and low efficacy associated with existing PTSD treatments, this study aims to develop a non-invasive game-based therapeutic approach based on the concepts of amnesic shadow and unconscious reactivation, designed to induce specific memory weakening.

2. Number of Participants, Study Duration, and Location

This study will involve 150 healthy Korean-speaking university students (ages 19–29) in Seoul, with corrected visual acuity of at least 0.5.
During the first visit, participants will wear a headset-type EEG (Electroencephalography) device while playing the "Arrow Avoidance Game," which was designed specifically for this study. The game session lasts approximately 30–40 minutes under researcher supervision.

The study will be conducted face-to-face at the following location:
"Yonsei University Campus Town S-Cube Center, Seodaemun-gu, Sinchon Station Road 29, Seoul."

After the initial visit, participants will return for two additional visits at 24 and 72 hours post-session.
- Visit 1 duration: ~40 minutes
- Visit 2 & 3 duration: ~20 minutes each
During follow-up visits, participants will answer brief oral questions.

3. Study Procedures and Methods
The procedures are as follows:

(1) Individuals who wish to participate will complete a screening questionnaire including gender, age, and school affiliation. Eligibility will be determined based on the results.
(2) If eligible, participants will undergo the consent process prior to gameplay. Participants may ask any questions before agreeing.
(3) After consent, the game ("Arrow Avoidance Game") will proceed as follows:

1) Learning Phase
   Participants will memorize 36 image pairs presented for a limited time.
   Duration: ~10–15 minutes.
2) Game Phase
   Participants will play the "Arrow Avoidance Game" designed for this study.
   Duration: ~5 minutes.
3) Test Phase
   Participants' memory of the image pairs from the Learning Phase will be assessed.
   Duration: ~15–20 minutes.

Total gameplay duration: ~30–40 minutes.

The experiment will be recorded and used only for research purposes.

Participants will wear a headset EEG device. A small amount of gel may be applied to localized scalp regions to remove keratin; this gel is completely safe.

Hair may become slightly disheveled during device placement. A saline-soaked styrofoam pad may come into direct contact with the scalp; both the saline and styrofoam materials are safe.

4. Expected Benefits

There is no direct personal benefit from participation.

However, the results may contribute to the development of a non-invasive, game-based PTSD digital therapeutic by providing evidence for the "specific memory weakening" mechanism.

5. Compensation

Participants who complete 2 out of 3 sessions will receive 50,000 KRW.

Participants who complete all 3 sessions will receive 70,000 KRW.

6. Potential Risks and Discomforts

Some images presented during the Learning Phase may cause mild emotional or affective discomfort.

To minimize discomfort, all images were screened to ensure they do not exceed predefined thresholds for violence or intensity.

A researcher will be present throughout the study.

Participants may withdraw at any time if they feel discomfort.

7. Early Withdrawal

Participants will be fully informed of the study purpose and methods before beginning.

If a participant wishes to withdraw before or during the study, they may do so freely by contacting the study staff.

8. Collection and Use of Personal Information

By signing this consent form, you agree to the collection and use of your personal (including sensitive) information for research purposes.

1) Purpose of Collection and Use

- To screen eligible participants
- To use anonymized statistical data when developing future non-invasive PTSD digital therapeutics focusing on memory weakening effects

2) Types of Personal (Sensitive) Information Collected

- Personal information: name, gender, age, contact information, school enrollment
- Sensitive information: corrected visual acuity, EEG data

3) Retention and Use Period

- Personal and sensitive information will be retained for 2 years and managed according to the Personal Information Protection Act.

4) Right to Refuse and Possible Disadvantages

- You may freely refuse collection or use of your (or your child's) personal information without disadvantage.
- However, refusal may restrict participation in this study.

5) Provision of Collected Information to Third Parties

- Collected personal/sensitive information will NOT be used for purposes other than this study, nor provided to third parties.
- Publications and presentations will use only anonymized statistical data without identifiable information.

9. Confidentiality

All personal information collected during the study will remain confidential.
If results are published or presented, no identifiable information will be disclosed.
Individuals involved in study oversight (study staff, monitors, IRB personnel) may access your records to verify study integrity, without violating confidentiality regulations.
By signing this form, you agree to allow such access.

연세대학교 YONSEI UNIVERSITY

All data will be stored securely and destroyed without delay once the retention period expires.

Data will be encrypted and stored according to Article 33 of the Statistics Act to prevent any unauthorized access.

All data will be destroyed 2 years after study completion.

If any issues arise during data management, corrective action will be taken immediately.

10. Voluntary Participation and Withdrawal

You may withdraw your consent at any time.

If you withdraw, no further data will be collected, and previously collected data will be destroyed and excluded from all future analyses.

Withdrawal will not result in any disadvantage or loss of benefits.

If new information arises that may influence your decision to continue, the research team will inform you promptly.

Only data collected after your consent will be used in analyses.

11. Contact Information

If you have questions or encounter issues during the study, please contact:

Researcher Name: Yu-Rang Park

Researcher Address: 50-1 Yonsei-ro, Seodaemun-gu, Seoul (03722)

Phone: 02-2228-2493

Research Staff Mobile: 010-5218-3284

For questions regarding participant rights, contact:

Severance Hospital Institutional Review Board (IRB): 02-2228-0430~4

Severance Hospital Human Research Protection Center (HPC): 02-2228-0450~4

YONSEI UNIVERSITY

CONSENT FORM

Study Title: Efficacy Verification of a Non-Invasive Game Therapeutic Intervention With a "Specific Memory Weakening" Effect for Alleviating PTSD Symptoms

1. I have read the information sheet and discussed it with the researcher.
2. I have been informed of the risks and benefits and received satisfactory answers to my questions.
3. I voluntarily agree to participate in this study.
4. I consent to the collection and processing of my personal information within the limits permitted by law and IRB regulations.
5. I agree that the principal investigator or delegated personnel, the research institution, funding agency, and government-designated bodies may access my confidential personal information for monitoring and oversight purposes.
6. I understand that I may withdraw from the study at any time without penalty.
7. My signature indicates that I have received a copy of this consent form, which I will keep until the end of the study.

Participant

Name:

Signature:

Date:

Person Obtaining Consent

Name:

Signature:

Date:

연세대학교
YONSEI UNIVERSITY
