# Supplementary material for: Shadow-Induced Forgetting in a Game-Based Paradigm on Nonclinical Adults and Its Effects on Consciousness, Emotional Valence, and Temporal Dynamics: Crossover Study
Source: JMIR Serious Games. 2025 Dec 30;13:e76946. doi: 10.2196/76946 (PMC12753131; doi:10.2196/76946)
Supplement: Multimedia Appendix 3 [file games-v13-e76946-s003.doc]

# Supplementary Data #2

# STUDY PROTOCOL

# Verification of the Efficacy of a Non-invasive Game Therapeutic Intervention with “Specific Memory Attenuation” Effects to Alleviate PTSD Symptoms

**Principal Investigator:**

Prof. Park Yu-Rang

Department of Biomedical Systems Informatics
Yonsei University College of Medicine

**Authors:**
Yoon-Jin Cho, Da-Won Seo, Ga-Hyun Kim, In-Seong Baek

**Supported by:**

Yonsei University College of Medicine

# TABLE OF CONTENTS

Page

[PROTOCOL TITLE](#__RefHeading___Toc323806624) 1

[1. OVERVIEW](#__RefHeading___Toc323806630)

[1.1 Background of the Study 4](#__RefHeading___Toc323806631)

[1.2 Objective of the Study 4](#__RefHeading___Toc323806632)

1.3 Principal Investigator and Co-Investigators 5

1.4 Institution Name and Address 5

1.5 Funding Sources 5

1.6 Study Period 5

1.7 Study Participants 6

1.8 Number of Participants and Basis for Calculation 6

1.9 Recruitment of Participants 6

1.10 Informed Consent 7

[2. METHOD](#__RefHeading___Toc323806636)

[2.1 Procedure 7](#__RefHeading___Toc323806650)

[2.1.1 Learning Phase 8](#__RefHeading___Toc323806652)

[2.1.2 Gaming Phase 11](#__RefHeading___Toc323806653)

[2.1.3 Test Phase 15](#__RefHeading___Toc323806654)

3. [DATA COLLECTION](#__RefHeading___Toc323806624) 16

4. [ANALYSIS PROCESS](#__RefHeading___Toc323806624) 16

[4.1 Overview 16](#__RefHeading___Toc323806650)

[4.2 Analysis Method 17](#__RefHeading___Toc323806650)

5. STATISTICAL [CONSIDERATIONS](#__RefHeading___Toc323806624) 17

[5.1 Observed variables 17](#__RefHeading___Toc323806650)

[5.2 Criteria and Methods for Evaluating Effectiveness 19](#__RefHeading___Toc323806650)

[5.3 Data Analysis and Statistical Methods 20](#__RefHeading___Toc323806650)

6. [PARTICIPANT RIGHTS AND CONFIDENTIALITY](#__RefHeading___Toc323806624) 22

[6.1 Anticipated Adverse Effects, Precautions, and Measures 22](#__RefHeading___Toc323806650)

[6.2 Withdrawal of Consent and Dropouts 22](#__RefHeading___Toc323806650)

[6.3 Risks and Benefits to Participants 22](#__RefHeading___Toc323806650)

[6.4 Compensation for Participation 22](#__RefHeading___Toc323806650)

[6.5 Safety Measures and Personal Information Protection 23](#__RefHeading___Toc323806650)

[6.6 Quality Control and Reliability Assurance (Data Safety Monitoring Plan) 23](#__RefHeading___Toc323806650)

7. [REFERENCES](#__RefHeading___Toc323806624) 23

**OVERVIEW**

**Background of the Study**

Traumatic memories can be intrusively triggered by various factors in daily life, causing distress [1]. Therefore, providing a safe and effective method to weaken the pairing between trauma and its associated triggers is of paramount importance, particularly for individuals suffering from post-traumatic stress disorder (PTSD). This study assumes the existence of an object associated with the memory (e.g., a victim of assault by a person wearing a “yellow hoodie” may form a pairing between the “yellow hoodie” and the traumatic memory) and proposes a game-based process designed to weaken this object-memory association. The process can be summarized as presenting a cue associated with the memory to be weakened during a “Reverse Game” that reduces hippocampal activity. By playing the game and experiencing hippocampal activity reduction, the memory stored in the hippocampus is reactivated by presenting the associated cue during reduced hippocampal activity, thereby weakening the object-memory pairing.

This study is significant for two reasons:

1. It verifies the induction of memory attenuation using a game-based process that is not monotonous, thus suggesting the possibility of providing “enjoyable therapy” for trauma patients.
2. By verifying the effectiveness of both consciously perceivable (Conscious) and unconsciously perceivable (Unconscious) stimulus presentations during the “stimulus presentation” process, the study demonstrates that cues can be presented subliminally (preconsciously) to mitigate the intrusive nature often associated with trauma treatments.

**1.2. Objective of the Study**

The objective of this study is to conduct an experiment on adults in their 20s and verify if memory attenuation occurs. The hypothesis is as follows: The unconscious or conscious presentation of certain cues during the game has induced memory attenuation of the target scene.

- - R_con Gist accuracy < Control Gist accuracy
  - R_con Similarity accuracy < Control Similarity accuracy
  - R_con Efficiency accuracy < Control Efficiency accuracy
  - R_uncon Gist accuracy < Control Gist accuracy
  - R_uncon Similarity accuracy < Control Similarity accuracy
  - R_uncon Efficiency accuracy < Control Efficiency accuracy

**1.3. Principal Investigator and Co-Investigators**

- - Principal Investigator: Prof. Park Yu-Rang (Associate Professor, Department of Biomedical Systems Informatics, Yonsei University College of Medicine) – Overall supervision and consultation
  - Co-Investigator: Mr. Baek In-Seong (Undergraduate student, Department of Biotechnology, Yonsei University) – Study planning and data analysis
  - Co-Investigator: Ms. Jo Yoon-Jin (Undergraduate student, Pre-medical Program, Yonsei University) – Study planning and data analysis
  - Co-Investigator: Ms. Park Nan (Undergraduate student, Department of Biotechnology, Yonsei University) – Study planning and data analysis
  - Co-Investigator: Mr. Seo Da-Won (Undergraduate student, Department of Biotechnology, Yonsei University) – Program implementation and study execution
  - Co-Investigator: Ms. Kim Ga-Hyun (Undergraduate student, Department of Applied Statistics, Yonsei University) – Clinical statistics and data analysis

**1.4. Institution Name and Address**

Yonsei University Campus Town S-Cube, No. 2, 29 Sinchonyeok-ro, Seodaemun-gu, Seoul, Republic of Korea

**1.5. Funding Sources**

- - Yonsei University Higher Education Innovation Institute Workstation
  - 2023 Integrated Medical Scientist Training Competition

**1.6. Study Period**

Expected study period: From the date of IRB approval until June 30, 2024.

|  | 23.09 | 23.10 | 23.11 | 23.12 | 241 | 24.2 | 24.3 | 24.04 | 24.05 | 24.06 |
| --- | --- | --- | --- | --- | --- | --- | --- | --- | --- | --- |
| IRB approval |  |  |  |  |  |  |  |  |  |  |
| Obtaining consent/Recruiting research participants |  |  |  |  |  |  |  |  |  |  |
| Experimental progress and research analysis |  |  |  |  |  |  |  |  |  |  |

**1.7. Study Participants**

The study aims to recruit a total of 150 participants, who must meet the following criteria:

1. Inclusion Criteria:
   - Yonsei University undergraduate/graduate students or alumni aged between 19 and 29
   - Corrected visual acuity of 0.5 or better in both eyes
   - No known health issues
   - Korean language proficiency
2. Exclusion Criteria:
   - Participants whose feedback cycle duration in the learning phase is abnormally long or short (falling outside the IQR of the distribution for all participants) are considered outliers and excluded.
   - If fitting the EEG device takes longer than 10 minutes, or if EEG data are not recorded properly, the participant is excluded from EEG analysis.

**1.8. Number of Participants and Basis for Calculation**

The target number of 150 participants was determined with reference to a previous study [2] that measured EEG activation in adult males to assess the therapeutic effect of neurofeedback (NF) training.

**1.9. Recruitment of Participants**

Recruitment announcements specifying participation conditions (age, corrected vision, Korean language proficiency) will be posted on the Yonsei University bulletin board and the Yonsei University online community (<https://everytime.kr/>). The announcement will clearly state the research objective—“Verification of memory attenuation through gaming”—and mention participant compensation. Volunteers will be encouraged to contact the research team for further information.

After confirming participants’ willingness, informed consent will be obtained in person. Participants will be reminded that they may withdraw at any time without any disadvantages. Compensation is structured as follows: KRW 50,000 if participants complete up to Test Phase 2, and KRW 70,000 if they complete up to Test Phase 3. No compensation is provided for completing only Test Phase 1.

**1.10. Informed Consent**

During recruitment, prospective participants will be informed of the consent procedure. Researcher Seo Da-Won will explain the study’s purpose, methods, and procedures in detail to any participant expressing willingness to participate. If they agree to participate, written informed consent will be obtained before commencing the study.

**2. Methods**

**2.1. Procedure**

The study will be conducted on healthy college students who voluntarily wish to participate. The experiment consists of three phases: Learning Phase (LP), Gaming Phase (GP), and Test Phase (TP).

- **Learning Phase (LP)**: Participants learn cue-target pairings through a self-feedback process.
- **Gaming Phase (GP)**: Participants play a “dodge obstacle game” designed to induce memory attenuation. During the reverse phase of the game, cues associated with certain memories are presented, aiming to weaken the memory pairing.
- **Test Phase (TP)**: Conducted three times (immediately after the gaming phase, 24 hours later, and 3 days later) to verify whether the cue-target pairing has been weakened.

Among the 36 cue-target pairs, 18 serve as a control group (not presented during the game), and the other 18 serve as the experimental group. Of these, 9 are presented consciously, and the remaining 9 are presented unconsciously (masked so that participants cannot consciously perceive them).


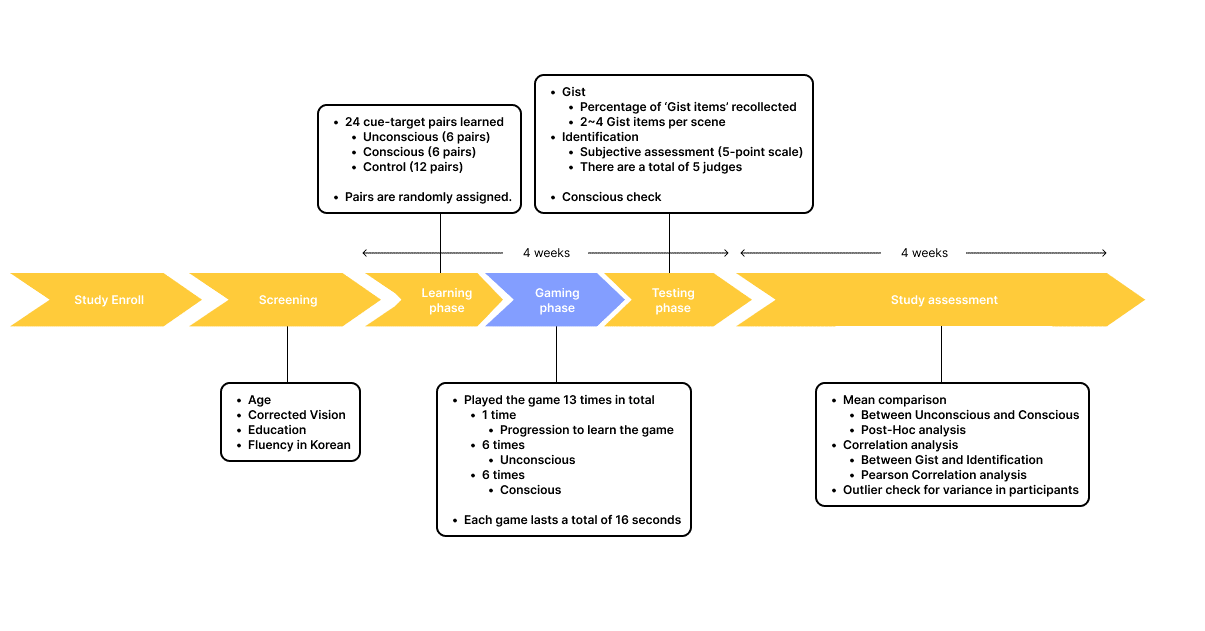


EEG and heart rate measurements will be taken during the gaming phase to verify the game’s effectiveness, and memory attenuation will be assessed using gist, similarity, and efficiency accuracy measures.

All tests will be conducted at Yonsei University Campus Town S-Cube No. 2 in Seodaemun-gu, Seoul. The experiments will be administered by researcher Seo Da-Won. Other researchers (Baek In-Seong, Park Nan, Jo Yoon-Jin, Kim Ga-Hyun) may observe as needed. All experimental procedures will be recorded via a webcam with participants’ prior consent.

**2.1.1. Learning Phase (LP)**

Participants view a cue and a target scene paired together and are instructed to remember the target scene and its details associated with the cue. After initial exposure, participants undergo a self-feedback process, pressing “O” (yes) if they recall the pairing clearly, or “X” (no) if not, allowing repeated exposure until they feel all pairs are learned.

|  | **Cue** | **Target** |
| --- | --- | --- |
| Explanation | - The object must be included in the target scene, but must not be associable with its situational context. - The object must be clearly and distinctly recognizable within the target scene. | - The target scene must possess a contextual narrative (e.g., a war scenario). - The target scene must contain two or more gist items. A “gist item” refers to an element essential to the scene’s story, which cannot be changed or removed without altering the main theme. - Each gist item must be distinctly and clearly identifiable. |

The researcher presents the cue and target to the participant in sequence using PowerPoint, instructing the participant to memorize the target by looking at the cue (Step 1). Afterwards, the participant undergoes a self-feedback process to fully master the cue-target pairs (Step 2). The procedure is as follows.

|  | **Powerpoint template** | **Explanation** | **Participant’s performance** |
| --- | --- | --- | --- |
| Step1 | 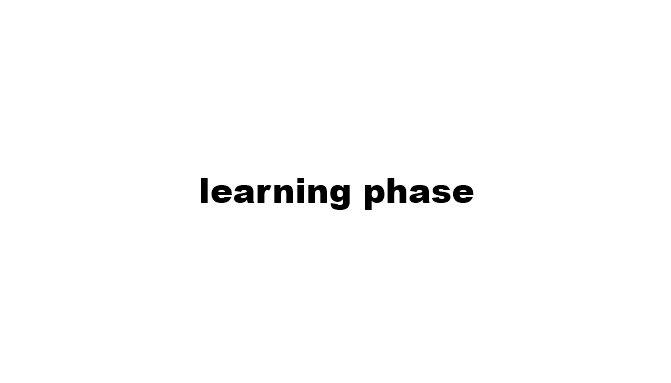 | - The experimenter explains the purpose of the learning phase as follows: “You will now enter the learning phase, during which you will study the provided dataset. After a simple game, some portions of the learned dataset will be randomly selected and presented as a simple quiz. We aim to examine the relationship between the game and the learning through this experiment.” | - A PowerPoint presentation is shown to the participant. On the left side of the screen, a cue is displayed, and on the right side, a target scene is presented. Following the instructions, the participant studies the cue and tries to remember the target scene and its specific elements. - Initial learning of the cue-target pairs takes place here. |
|  | 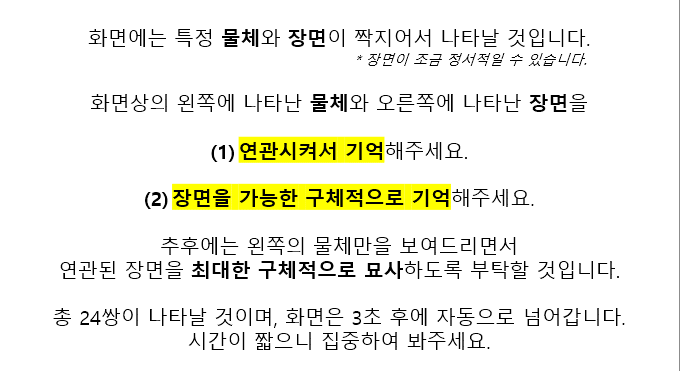 | - The participant is informed that specific objects and scenes are paired on the screen. - The participant is instructed to remember the object on the left in association with the scene on the right, as this association will be tested in later stages of the experiment. - The experimenter explains the display time and subsequent confirmation process as indicated on the screen. |  |
|  | 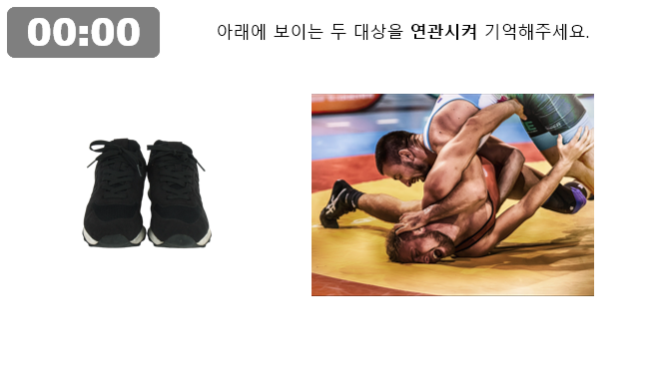 | - Each cue-target pair is displayed for 3 seconds, with the cue on the left and the target on the right. |  |
| Step2 | 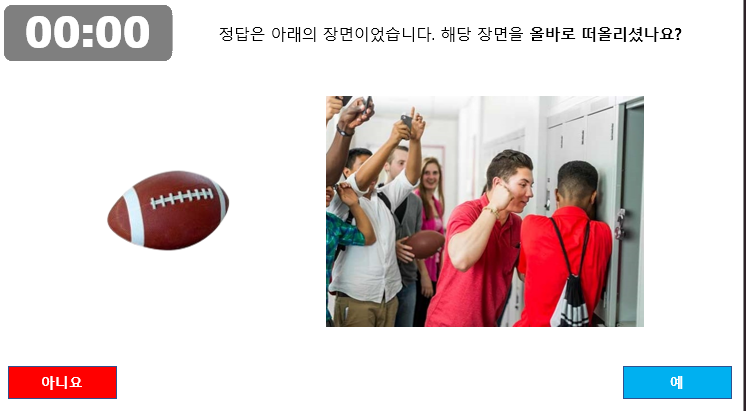 | - The experimenter explains that this step involves self-feedback on the previously learned object-scene pairings. - The previously learned objects and their corresponding scenes are presented in turn. If the participant can specifically recall the scene upon seeing the object, they press “O”; if not, they press “X.” Any pairing marked with “X” is re-presented during the learning phase for reinforcement. | - Through the repetition of the pairings presented in Step 1, the participant reinforces the association between the cue and the target. - When the participant believes they have fully learned all cue-target pairs, the learning phase ends. |

**2.1.2. Gaming Phase (GP)**

- **GAME**

In this study, the “obstacle-avoidance game” is used with the aim of presenting certain images during a “reverse phase,” in which the direction of the character’s movement does not match the direction of the arrow keys pressed by the participant, inducing response inhibition. The game is provided to participants via a laptop, and the participant uses the laptop’s arrow keys to play.

This game was implemented using the web-based cloud IDE platform Goorm IDE (<https://ide.goorm.io/>). The game’s UI design is as follows:

| 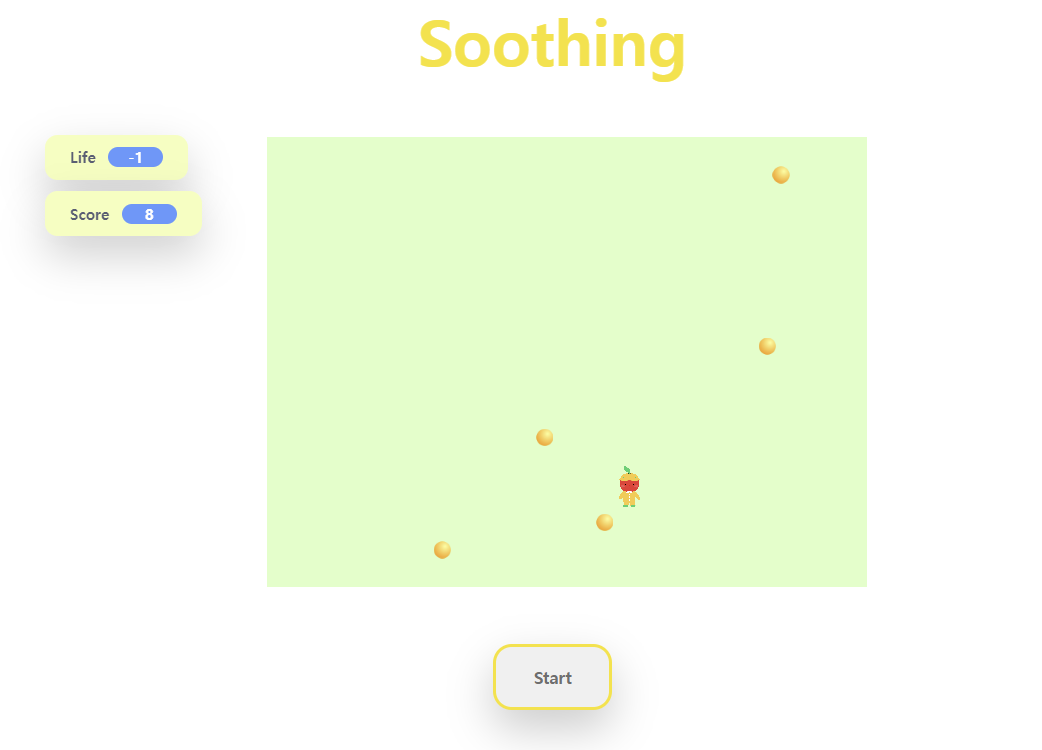  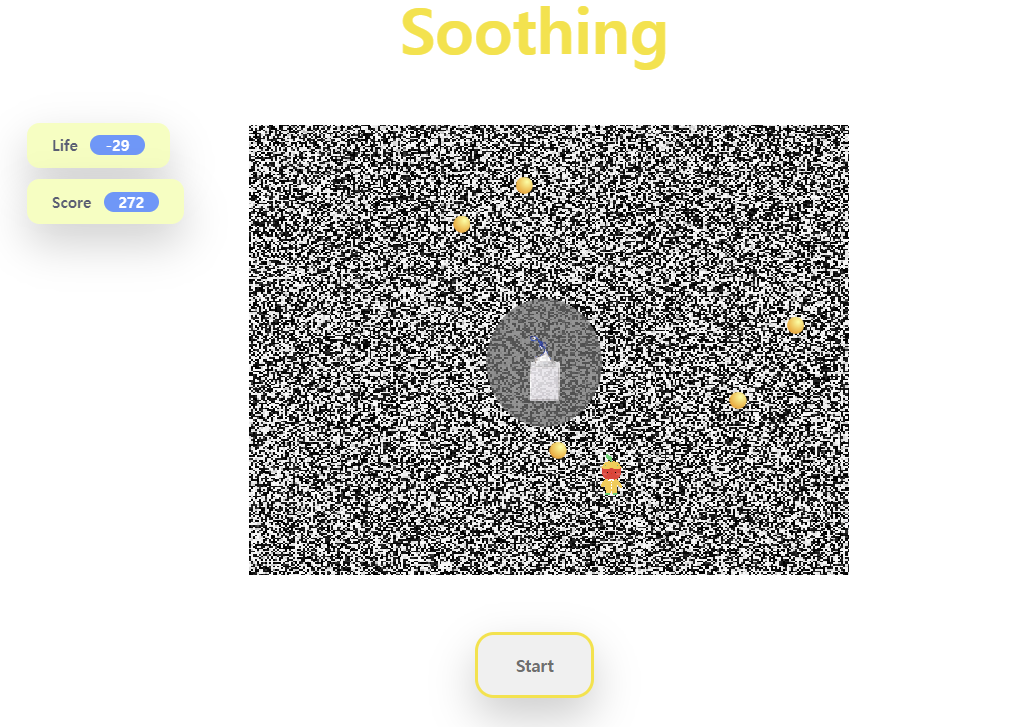 |
| --- |
| - The obstacle-avoidance game requires the character to evade obstacles coming from all directions. For 8 seconds during the forward phase, the direction of the character’s movement matches the arrow keys. Afterwards, for 8 seconds during the reverse phase, the arrow keys function in the opposite direction, causing the character to move contrary to the participant’s intended input. - During the reverse phase, cues learned in the learning phase are presented in the game’s background. Randomly, 9 times the participant is presented with a consciously perceivable (Conscious) stimulus, and 9 times with a stimulus that is not consciously perceivable (Unconscious). The method for presenting the unconscious stimulus is adapted from a previous study [3]: the image is shown for 0.0167 seconds with 90% background transparency and 25% luminance. - The game is played at a 1:0.9 width-to-height aspect ratio. If the game character hits an obstacle, the life count decreases. The participant can check their remaining life in real-time. - To minimize boredom, a sound effect is played each time the character is hit by an obstacle, causing life to decrease. If the character avoids obstacles and they hit the wall, the participant’s score increases by the number of obstacles hitting the wall. - One round of the game consists of 8 seconds of the forward phase and 8 seconds of the reverse phase, totaling 16 seconds. |

The sequence of each 16-second round is as follows:

| 1 | Forward Phase  (8 seconds) | To avoid obstacles, the participant presses the left or right arrow keys to move the character. If the character is hit by an obstacle, the life score decreases by 1. |
| --- | --- | --- |
| 2 | Reverse Phase  (8 seconds) | The rule that the character must move left or right to avoid obstacles remains the same, but the direction in which the character moves is set to be opposite to the direction of the arrow keys pressed by the participant. For example, to move the character to the right, the participant must press the left arrow key. This phase aims at “specific memory attenuation” induced by the cue presented in the game’s background. |

The game is played a total of 19 times. The first round is a practice round for the participant to become familiar with the rules. For the subsequent 18 rounds, 9 rounds present conscious stimuli and the other 9 rounds present unconscious stimuli, targeting “specific memory attenuation” for the presented cue.

- **EEG Measurement**

During the participant’s gameplay, EEG measurements will be taken. The experimental procedure is adapted from previous studies that measured EEG during conscious recollection processes [4]. Participants will be seated in a sound- and light-attenuated room, facing a display monitor. They will be instructed in advance that EEG recordings will be taken while playing the game. The Emotiv EPOC wireless headset does not require a wet cap to improve conductivity. In this study, we will use the Emotiv EPOC wireless headset (Emotiv Inc.), which has 14 electrodes (AF3, F7, F3, FC5, T7, P7, O1, O2, P8, T8, FC6, F4, F8, and AF4) positioned according to the American EEG Society standard. The sampling rate is 128 Hz, the bandwidth is 0.2–45 Hz, and digital notch filters are applied at 50 Hz and 60 Hz.


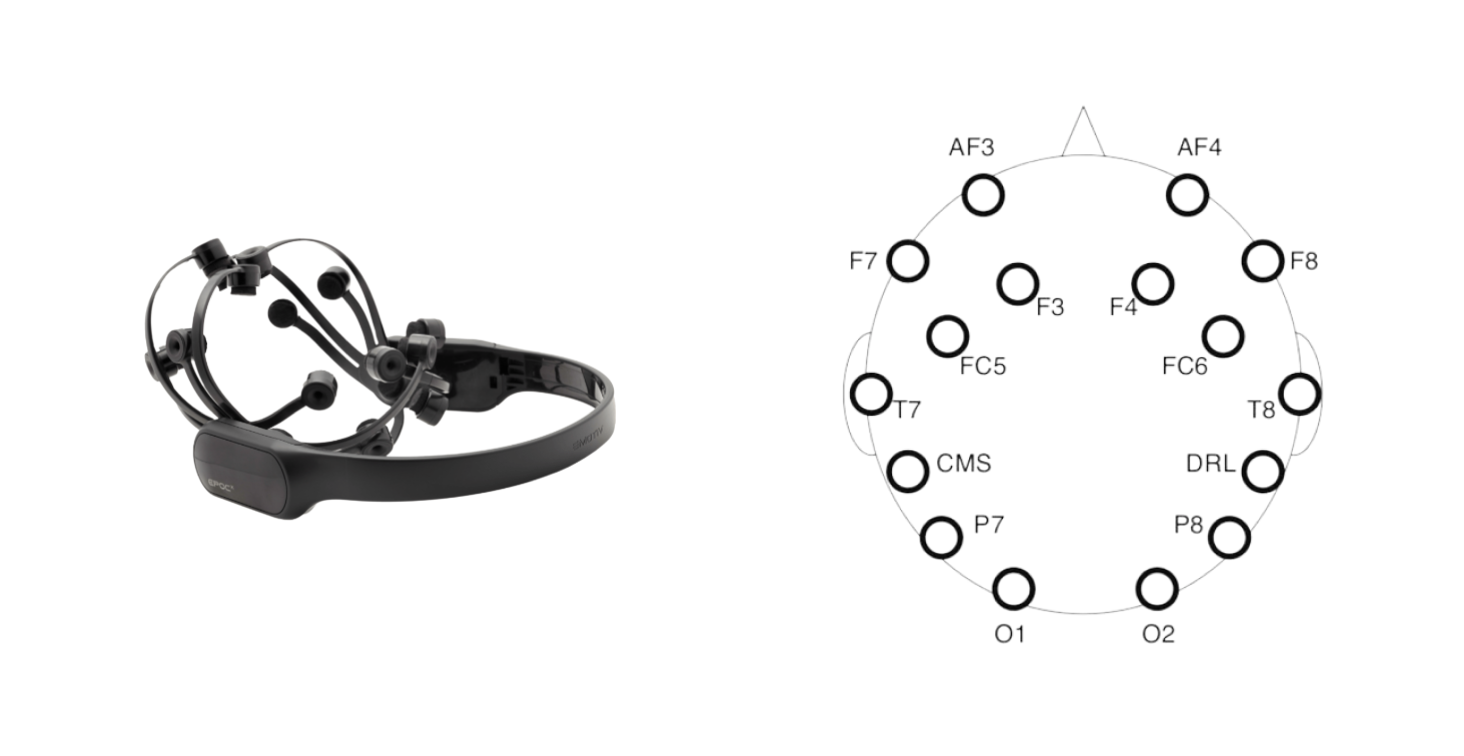


- **Heart Rate Measurement**

Heart rate measurements will also be taken during the participant’s gameplay, following a specific protocol.
We will use a wearable tracker, the Fitbit Charge 5, worn on the participant’s wrist. The Fitbit Charge 5 includes an optical heart rate monitor and a 3-axis accelerometer for tracking movement patterns, enabling continuous measurement of changes in the participant’s heart rate over time [5]. This device is cost-effective, user-friendly, and capable of measuring heart rate over extended periods [6].


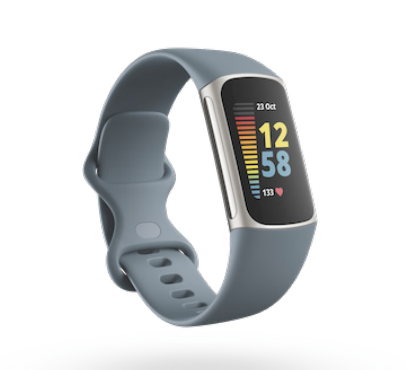


For heart rate measurements, we will follow the protocol provided by Fitbit [7]. Before the start of the Learning Phase, the participant’s left wrist will be fitted with the Fitbit device. We will record the start times of the Forward Phase and Reverse Phase of the Game Phase to synchronize heart rate data with the experimental events.

**2.1.3. Test Phase (TP)**

The Test Phase is conducted in three stages (1st, 2nd, and 3rd) to verify whether the game performed by the participant actually induced memory attenuation between certain cues and targets. Each test involves 12 cue-target pairs, conducted according to the following schedule:

| **Phase** | **Explanation** |
| --- | --- |
| **Test Phase 1** | Immediately after the Game Phase |
| **Test Phase 2** | 24 hours after the Game Phase ends |
| **Test Phase 3** | 3 days after the Game Phase ends |

At each stage, measurements are taken of the control group, Experimental Group 1 (Conscious), and Experimental Group 2 (Unconscious) from the datasets learned during the learning phase. The specific experimental and control sets to be tested are randomly selected using <https://www.randomizer.org/>.

**3. DATA COLLECTION**

At each stage, the following data are collected. The laptop screen and the participant’s frontal view during the learning-gaming-test phases are recorded. During the test phase, a cue is presented for 15 seconds, and the participant is asked to describe the target scene with as many elements as possible. With the participant’s prior consent, the response is recorded using a smartphone. EEG data are also collected while the participant wears the EEG device during the gaming-test phases. If fitting the EEG device takes more than 10 minutes, the attempt is discontinued, and the experiment proceeds to the next phase.

Heart rate data are obtained by having the participant wear a Fitbit device on their wrist. Using the Fitbit Software Development Kit (SDK), the recorded heart rate data are retrieved from the server and saved as a CSV file. Afterward, outlier detection and removal of missing data are performed.

**4. ANALYSIS PROCESS**

**4.1. Overview**

The memory accuracy derived from the test phase analysis is measured using the following metrics: Gist accuracy, Similarity accuracy, and Efficiency accuracy. Gist accuracy measures whether the participant can recall the existing detailed elements of the scene, and Efficiency accuracy measures whether the scene is described efficiently by the ratio of the number of recalled gist items to the total number of words spoken. Similarity accuracy measures the similarity between the participant’s verbal response and the text describing the image generated by AI. The first two metrics are concepts proposed in a previous study [8], and additional details have been designed for this experiment’s purpose regarding the Efficiency and Similarity measures.

**4.2. Analysis Method**

The specific evaluation procedures are as follows:

|  | **Criterion** | Evaluation Procedure |
| --- | --- | --- |
| **Gist** | The average of the maximum similarity values between the participant’s spoken words and the selected gist words | 1. The experimenters reach a mutual agreement on the gist for each image. 2. The 12 external participants are shown each image and asked to provide a detailed description. 3. All responses are translated into English. 4. Various expressions referring to the same gist are identified and grouped into a single category (gist extension). 5. In this experiment, the highest similarity value found among the words in that category is defined as the Gist score. |
| **Similarity** | Comparison with AI-Generated Text | After generating a descriptive text for the image using AI, the similarity between this AI-generated text and the participant’s response is measured. |
| **Efficiency** | Number of Recalled “Gist Items” / Total Number of Spoken Words | By measuring the ratio of gist items recalled to the total amount of spoken words, the efficiency with which the participant describes the scene can be evaluated. |

**5. STATISTICAL CONSIDERATIONS**

**5.1. Observed Variables**

The flow chart of the subjects to be observed in this study is presented below.


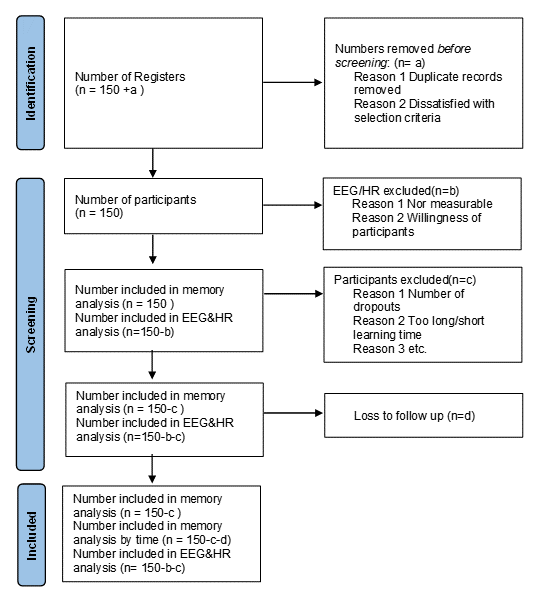


| 1. Items collected through the questionnaire: Korean language proficiency, corrected visual acuity, age, and educational level 2. Items collected during the experiment:  - Changes in EEG and physiological signals during the reverse phase of the game - The duration of the feedback cycle in the learning phase - Audio and video recordings of the participant’s description of the target scene evoked by a specific cue (immediately after the game and two days later) - Whether the participant perceived any subliminal or preconscious stimuli presented during the game |
| --- |

**5.2. Criteria and Methods for Evaluating Effectiveness**

The study aims to confirm the following points:

| - **Aim 1**. Does the therapeutic intervention delivered through the game induce a significant reduction in the participant’s memory retention? - **Aim 2.** If so, how long does this reduction in memory retention persist after the game is played? - **Aim 3.** Is there a difference in the degree of memory reduction induced by consciously presented stimuli versus unconsciously presented stimuli? - **Aim 4.** Are there significant changes in the participant’s objective physiological signals during the reverse phase of the game? - **Aim 5.** Does the memory reduction differ depending on the emotional valence (positive or negative) of the presented images? |
| --- |

Based on the above objectives, the following evaluation criteria and methods are proposed:

| **Aim 1** | - Demonstrate that the memory accuracy of the experimental group (with therapeutic intervention) is lower than that of the control group. (t-test, p<0.05) - Compare the patterns of change between forward and reverse phases for groups showing treatment effects (Aim 1 satisfied) and those not showing treatment effects (Aim 1 unsatisfied). (Group analysis) |
| --- | --- |
| **Aim 2** | - Compare the memory accuracy of the experimental group exposed to conscious stimuli and the experimental group exposed to unconscious stimuli against the control group to verify that memory weakening is effective even when stimuli are presented unconsciously and non-invasively. (t-test, p<0.05) - Compare the effects induced by conscious and unconscious stimulus presentations. |
| **Aim 3** | - Compare and analyze the accuracy at Test 1, Test 2, and Test 3 to observe the temporal persistence of the memory weakening effect of the game. (pairwise t-test, p<0.05) |
| **Aim 4** | - Show that there are significant differences in EEG signals between the learning phase (before the game), the forward phase (during the game), and the reverse phase (during the game).   1. Differences among Learning vs. Game_Forward vs. Game_Reverse phases (ANOVA, p<0.05)   2. Significant changes in EEG patterns between forward and reverse phases (t-test, p<0.05)   3. Once changes in EEG during the reverse phase are confirmed, perform a comparative analysis between reverse_con and reverse_uncon. (t-test, p<0.05) |
| **Aim 5** | - Show that there is a difference in the memory weakening effect of the game for images with positive emotional valence versus those with negative emotional valence. (t-test, p<0.05) |

**5.3. Data Analysis and Statistical Methods**

To verify the game’s effect, an independent samples t-test will be conducted between the experimental group (R_con, R_uncon) and the control group. This test will be performed three times, once for each of the three metrics (gist, similarity, efficiency). If P-value < 0.05, it can be concluded that the average score of the experimental group is significantly lower than that of the control group, thereby confirming the effectiveness of the game.

A repeated measures ANOVA will be performed for the three metrics to compare memory recall accuracy across the three groups (R_con, R_uncon, Control). If a difference among the three groups is found (P<0.05), a post hoc analysis will be conducted. If there is a significant interaction over time, separate ANOVAs will be conducted for each test time point (T1, T2, T3). The subsequent procedures remain the same. Through a Tukey test, pairs of groups showing differences will be identified. A paired t-test will then be conducted within each pair to determine whether the mean scores of R_con and R_uncon are significantly lower than those of the Control group and to clarify the relationship between R_con and R_uncon. The significance level is set at p=0.05. Additionally, Cohen’s effect size will be measured to determine the magnitude of the observed differences.

Pairwise tests will be performed on the T1, T2, and T3 results, and repeated measures ANOVA and t-tests will be conducted to confirm the persistence of the memory attenuation effect over time after the game.

Furthermore, to investigate whether there is a difference in the memory attenuation effect between positive and negative images, the above analysis steps will be repeated separately for positive and negative images, and the results will be compared.

Correlation analysis will also be performed to examine the relationships among the three metrics, verifying whether these three metrics are appropriate measures for describing scenes.

Before conducting these tests, assumptions of normality, homogeneity of variance, independence, and sphericity (for RM ANOVA) will be checked. If these assumptions are not met, non-parametric alternatives such as the Kruskal-Wallis, Friedman, or Wilcoxon signed-rank tests will be used, applying the same underlying hypotheses.

For the measured EEG data, preprocessing steps such as filtering, DC offset removal, ICA, and artifact removal will be conducted. After preprocessing, power spectral analysis will be applied to the EEG data. Using a Fast Fourier Transform (FFT), changes in relative power values for each frequency band will be observed to compare the learning phase (pre-game), forward phase, and reverse phase. ANOVA will be used to confirm whether there are significant differences in EEG values among these three events. After testing assumptions of normality and homogeneity of variance, a paired t-test will be performed to statistically verify differences in mean values between events. When comparing forward and reverse phases, since the reverse phase can be divided into three groups (con+uncon/con/uncon), three t-tests will be conducted. Differences in each frequency band between forward and reverse phases will be examined, and the characteristics of EEG signals that show significant differences will guide the interpretation of the results. Additionally, a comparative analysis between reverse_con and reverse_uncon will be conducted to examine differences between consciously and unconsciously presented images.

Since each participant plays the game 18 times, data from each of the 18 forward and reverse segments will be obtained. Analysis will be performed by averaging these repeatedly measured potentials through Event-Related Potential (ERP) methods. Characteristic peak values will be identified, and paired t-tests will be conducted between forward and reverse phases.

Furthermore, connectivity analysis will be performed to confirm functional connectivity differences between segments. Simple descriptive statistics such as mean values, peak means, and standard deviations per channel and event will also be compared.

Topography analysis will be performed by averaging values from all channels over time to determine how EEG power changes across channel regions. The results of these analyses will then be visualized.

If parametric methods are required, normality, homogeneity of variance, independence, and sphericity (for RM ANOVA) will be tested beforehand. If these assumptions are not satisfied, non-parametric methods such as Kruskal-Wallis, Friedman, or Wilcoxon signed-rank tests will be used under the same hypotheses.

For HR analysis using Fitbit, the start times of the Forward Phase and Reverse Phase will be recorded each time the participant plays the game (36 records in total). After removing outliers and missing values from the measured HR data, the mean heart rate for each phase (learning phase, forward phase, reverse phase) will be calculated based on the recorded timestamps. The mean HR values during each segment (learning phase, forward phase, reverse phase) will be statistically examined for significant differences. After checking the assumptions of normality and homogeneity of variance, a paired t-test will be conducted to statistically determine differences in mean values between the segments.

**6.** **PARTICIPANT RIGHTS AND CONFIDENTIALITY**

**6.1. Anticipated Adverse Effects, Precautions, and Measures**

During the learning phase, the target scenes presented along with CUE-TARGET pairs are mostly affectively negative, which may cause discomfort for some participants. In such cases, participants may request to discontinue the experiment. Other than the above scenario, it is deemed unlikely that any direct harm will be caused to participants.

**6.2. Withdrawal of Consent and Dropouts**

The criteria for withdrawing consent are as follows:
Even if participants voluntarily consent to the study, they may withdraw their consent at any time during the study without any penalty. If a participant expresses the desire to withdraw consent, that participant’s personal information and related data will not be used for analysis.

**6.3. Risks and Benefits to Participants**

Apart from the potential psychological discomfort caused by target scenes in the study’s program, there are no known risks. This potential risk will be monitored continuously by the researcher to prevent any dangerous situations for the participants in advance.

There is no direct benefit to the participants from participating in this study. However, if the study’s effectiveness is confirmed, it could contribute to the development of non-invasive game-based therapeutic interventions for memory attenuation.

**6.4. Compensation for Participation**

Participants who complete up to Test Phase 2 will receive KRW 50,000, and participants who complete up to Test Phase 3 will receive KRW 70,000. No compensation will be given if the participant only completes Test Phase 1. If the participant withdraws consent during the experiment, no compensation will be provided. If a participant drops out for reasons unrelated to the study without notice, no compensation will be provided, and this will be communicated in advance.

**6.5. Safety Measures and Personal Information Protection**

Participants will be informed in advance of the study’s purpose, potential risks during the research, and the collection of personal information before obtaining their consent. The data collected through this study will not be disclosed to anyone other than the research personnel (Seo Da-Won, Baek In-Seong, Jo Yoon-Jin, Park Nan, Kim Ga-Hyun, and the 5 external evaluators). The personal information of the participants will not be viewed or leaked by the 5 evaluators.

In accordance with the Bioethics Law, all research-related records will be retained for 3 years after the completion of the study and then destroyed.

**6.6. Quality Control and Reliability Assurance (Data Safety Monitoring Plan)**

Since this study poses a low level of risk, monitoring will be conducted by a researcher (Seo Da-Won) delegated by the PI under the PI’s supervision. Every 4 weeks, the source documents, CRFs, and protocol will be compared to ensure data completeness, and the participants’ safety data will be reviewed.

**REFERENCES**

1. Zhu Z, Anderson MC, Wang Y. Inducing forgetting of unwanted memories through subliminal reactivation. Nat Commun. 2022;13(1):6496.

2. Abadie A. Semiparametric difference-in-differences estimators. The review of economic studies. 2005;72(1):1-19.

3. Brewin CR, Gregory JD, Lipton M, Burgess N. Intrusive images in psychological disorders: characteristics, neural mechanisms, and treatment implications. Psychological review. 2010;117(1):210.

4. Omidvarnia AH, Azemi G, Boashash B, Toole JMO, Colditz P, Vanhatalo S, editors. Orthogonalized partial directed coherence for functional connectivity analysis of newborn EEG. Neural Information Processing: 19th International Conference, ICONIP 2012, Doha, Qatar, November 12-15, 2012, Proceedings, Part II 19; 2012: Springer.

5. Fuller D, Colwell E, Low J, Orychock K, Tobin MA, Simango B, et al. Reliability and Validity of Commercially Available Wearable Devices for Measuring Steps, Energy Expenditure, and Heart Rate: Systematic Review. JMIR Mhealth Uhealth. 2020;8(9):e18694.

6. Bian J, Guo Y, Xie M, Parish AE, Wardlaw I, Brown R, et al. Exploring the association between self-reported asthma impact and Fitbit-derived sleep quality and physical activity measures in adolescents. JMIR mHealth and uHealth. 2017;5(7):e7346.

7. Diaz KM, Krupka DJ, Chang MJ, Peacock J, Ma Y, Goldsmith J, et al. Fitbit®: An accurate and reliable device for wireless physical activity tracking. International journal of cardiology. 2015;185:138.

8. Küpper CS, Benoit RG, Dalgleish T, Anderson MC. Direct suppression as a mechanism for controlling unpleasant memories in daily life. Journal of Experimental Psychology: General. 2014;143(4):1443.
