# Supplementary material for: Shadow-Induced Forgetting in a Game-Based Paradigm on Nonclinical Adults and Its Effects on Consciousness, Emotional Valence, and Temporal Dynamics: Crossover Study
Source: JMIR Serious Games. 2025 Dec 30;13:e76946. doi: 10.2196/76946 (PMC12753131; doi:10.2196/76946)
Supplement: Multimedia Appendix 4 [file games-v13-e76946-s004.docx]

**Supplementary Data #3**

**Statistical Analysis Plan**

**Table of Contents**

[1 Trial Identifications 2](#_Toc185513669)

[2 Abbreviations and Definitions 2](#_Toc185513670)

[3 Introduction 3](#_Toc185513671)

[3.1 Preface 3](#_Toc185513672)

[3.2 Purpose of the analyses 3](#_Toc185513673)

[4 Study Objectives and Endpoints 4](#_Toc185513674)

[4.1 Study Objectives 4](#_Toc185513675)

[4.2 Endpoints 4](#_Toc185513676)

[4.3 Derived variables 6](#_Toc185513677)

[5 Study Methods 7](#_Toc185513678)

[5.1 General Study Design and Plan 7](#_Toc185513679)

[5.2 Inclusion-Exclusion Criteria and General Study Population 8](#_Toc185513680)

[5.3 Study Variables 8](#_Toc185513681)

[6 Sample Size 11](#_Toc185513682)

[7 General Considerations 11](#_Toc185513683)

[7.1 Timing of Analyses 11](#_Toc185513684)

[7.2 Analysis Populations 11](#_Toc185513685)

[7.2.1 Full Analysis Population 11](#_Toc185513686)

[7.2.2 Per Protocol Population 11](#_Toc185513687)

[7.2.3 Safety Population 12](#_Toc185513688)

[7.3 Covariates and Subgroups 12](#_Toc185513689)

[7.4 Missing Data 13](#_Toc185513690)

[7.5 Interim Analyses and Data Monitoring 13](#_Toc185513691)

[8 Summary of Study Data 13](#_Toc185513692)

[8.1 Subject Disposition 14](#_Toc185513693)

[8.2 Protocol Deviations 14](#_Toc185513694)

[8.3 Demographic and Baseline Variables 15](#_Toc185513695)

[8.4 Concurrent Illnesses and Medical Conditions 15](#_Toc185513696)

[8.5 Prior and Concurrent Medications 16](#_Toc185513697)

[8.6 Treatment Compliance 16](#_Toc185513698)

[9 Efficacy Analyses 16](#_Toc185513699)

[9.1 Primary Efficacy Analysis 16](#_Toc185513700)

[9.2 Secondary Efficacy Analyses 17](#_Toc185513701)

[9.3 Exploratory Efficacy Analyses 19](#_Toc185513702)

[10 Safety Analyses 19](#_Toc185513703)

[10.1 Extent of Exposure 19](#_Toc185513704)

[10.2 Adverse Events 20](#_Toc185513705)

[11 Figures 20](#_Toc185513706)

[12 Reporting Conventions 20](#_Toc185513707)

[13 Technical Details 20](#_Toc185513708)

[14 Summary of Changes to the Protocol 21](#_Toc185513709)

[15 References 22](#_Toc185513710)

[16 Listing of Tables, Listings and Figures 23](#_Toc185513711)

# Trial Identifications

| TRIAL FULL TITLE | Verification of the Efficacy of a Non-invasive Game Therapeutic Intervention with “Specific Memory Attenuation” Effects to Alleviate PTSD Symptoms |
| --- | --- |

# Abbreviations and Definitions

| *AE* | *Adverse Event* |
| --- | --- |
| *SAP* | *Statistical Analysis Plan* |
| *ShIF* | *Shadow-induced forgetting* |

# Introduction

## Preface

Traumatic memories can be intrusively triggered by various factors in daily life, causing distress [1]. Therefore, providing a safe and effective method to weaken the pairing between trauma and its associated triggers is of paramount importance, particularly for individuals suffering from post-traumatic stress disorder (PTSD). This study assumes the existence of an object associated with the memory (e.g., a victim of assault by a person wearing a “yellow hoodie” may form a pairing between the “yellow hoodie” and the traumatic memory) and proposes a game-based process designed to weaken this object-memory association. The process can be summarized as presenting a cue associated with the memory to be weakened during a “Reverse Game” that reduces hippocampal activity. By playing the game and experiencing hippocampal activity reduction, the memory stored in the hippocampus is reactivated by presenting the associated cue during reduced hippocampal activity, thereby weakening the object-memory pairing.

This study is significant for two reasons:

1. It verifies the induction of memory attenuation using a game-based process that is not monotonous, thus suggesting the possibility of providing “enjoyable therapy” for trauma patients.
2. By verifying the effectiveness of both consciously perceivable (Conscious) and unconsciously perceivable (Unconscious) stimulus presentations during the “stimulus presentation” process, the study demonstrates that cues can be presented subliminally (preconsciously) to mitigate the intrusive nature often associated with trauma treatments.

## Purpose of the analyses

These analyses will assess the efficacy of a game process designed to induce memory weakening non-invasively, comparing it with a control group through a clinical study conducted on general college students.

# Study Objectives and Endpoints

## Study Objectives

(ICH E3; 8.)

The purpose of this study is to evaluate the effects of cue exposure conditions (Conscious, Unconscious, Control) on short-term and long-term memory suppression and to determine whether these effects are modulated by emotional valence (Positive, Negative).

Specifically, this study aims to:

1. Short-Term Effects: Assess the immediate impact of conscious and unconscious cue exposure on memory suppression compared to a control condition, using METEOR scores measured immediately after the intervention (Day 1).
2. Long-Term Effects: Investigate whether the suppression observed on Day 1 persists over time (Day 2, Day 4), reflecting long-term forgetting trajectories across exposure conditions.
3. Interaction with Emotional Valence: Determine whether the effects of cue exposure conditions differ for images with positive versus negative emotional valence, highlighting potential emotional modulation of memory suppression processes.
4. Neural Correlates: Explore the underlying neural mechanisms of memory suppression by analyzing EEG activity, focusing on comparisons between exposure conditions (Conscious vs. Unconscious) and valence (Negative vs. Positive) across key frequency bands (alpha, beta, theta, delta, gamma).

This comprehensive approach will provide insights into both the behavioral and neural dimensions of memory suppression, offering a deeper understanding of how cue exposure and emotional content influence forgetting processes.

## Endpoints

(ICH E9; 2.2.2)

1. Primary Endpoint

- Endpoint: Short-term memory suppression (METEOR scores).
- Definition: Memory suppression will be assessed using METEOR scores, a measure of semantic similarity reflecting accurate and context-rich recall.
- Time Point: Day 1 (immediately post-intervention).
- Comparisons:
- Cue exposure conditions: Conscious vs Control, Unconscious vs Control.
- Subgroup analysis: Positive vs Negative emotional valence.
- Hypothesis:
- Total Image Group: Conscious < Control, Unconscious < Control (one-tailed t-test).
- Negative Image Group: Conscious < Control, Unconscious < Control (one-tailed t-test).
- Positive Image Group: Conscious < Control, Unconscious < Control (one-tailed t-test).

2. Secondary Endpoints

- Endpoint 1: Long-term memory suppression (METEOR scores over time).
- Definition: Assessment of METEOR scores on Day 2 (24 hours post-intervention) and Day 4 (72 hours post-intervention) to evaluate memory suppression persistence.
- Time Points: Day 1, Day 2, Day 4.
- Analysis: Repeated paired t-tests (Conscious vs Control, Unconscious vs Control).
- Endpoint 2: Group differences in memory performance trajectories.
- Definition: Evaluation of memory decline over time using Difference-in-Differences (DID) analysis, adjusted for covariates such as: feedback Cycle Times (duration of self-feedback process during the learning phase), and frequency of Image Repetition (number of image viewings during the learning phase).
- Purpose: To supplement time-specific t-test comparisons and better understand group differences in forgetting trajectories.
- Endpoint 3: Emotional valence-based comparisons.
- Definition: Paired two-tailed t-tests comparing METEOR scores across valence conditions at each time point:
- Negative Treatment vs Positive Treatment
- Negative Conscious vs Positive Conscious.
- Negative Unconscious vs Positive Unconscious.
- Negative Conscious vs Positive Unconscious.
- Negative Unconscious vs Positive Conscious.

3. Exploratory Endpoints

- Endpoint: EEG-based neural activity analysis.
- Definition: Neural activity will be compared across cue exposure conditions and emotional valence groups using EEG power spectrum density.
- Frequency Bands: Alpha, beta, theta, delta, and gamma.
- Comparisons:
- Cue Exposure: Conscious vs Unconscious phases.
- Emotional Valence: Negative vs Positive image groups.
- Statistical Method: Two-tailed Wilcoxon signed-rank test for paired comparisons.

## Derived variables

1. Timepoint-Specific METEOR/Gist Score Comparisons

- Definition: A derived variable representing the differences in METEOR and Gist scores between groups (e.g., Control vs Conscious, Control vs Unconscious) at each assessment timepoint: Day 1, Day 2, and Day 4.

This variable allows for the evaluation of whether group differences persist, diminish, or change over time.

2. Timepoint-Specific METEOR/Gist Score Change

- Definition: A derived variable representing the within-group change in METEOR and Gist scores across the three assessment timepoints (Day 1, Day 2, and Day 4).

This variable is used to measure the progression or decline of memory performance over time for each group.

3. EEG-Based Neural Activity Comparisons

- Definition: A derived variable representing the differences in EEG power spectrum density across the following conditions:
- Cue Exposure Condition: Conscious vs Unconscious phases.
- Emotional Valence: Negative vs Positive image categories.

This variable enables the identification of significant neural activity differences associated with exposure conditions and emotional valence.

# Study Methods

## General Study Design and Plan

(ICH E3;9)

This study employs a **within-subject design**, where each participant serves as their own control. This design reduces inter-subject variability, allowing for more precise comparisons across conditions. The study evaluates the interaction between the following three factors, with participants learning a total of 36 cue-target image pairs, categorized as follows:

- **Exposure Condition:** Control, Conscious, Unconscious
- **Valence:** Positive, Negative
- **Test Timepoint:** Day 1 (immediately post-Gaming), Day 2 (24 hours post-Gaming), Day 4 (72 hours post-Gaming)

This factorial design facilitates the assessment of how exposure condition, emotional valence, and time influence memory performance (measured by METEOR scores) and neural activity (assessed through EEG). The within-subject approach ensures that each participant’s performance under one condition can be directly compared to their performance under other conditions.

## Inclusion-Exclusion Criteria and General Study Population

(ICH E3;9.3. ICH E9;2.2.1)

1. **Inclusion Criteria**

- Average corrected binocular visual acuity of 0.5 or higher.
- No notable health issues.
- Proficiency in Korean language.

1. **Exclusion Criteria**

- Participants whose feedback cycle times during the learning phase are too long or too short (outliers determined as those outside the interquartile range [IQR] of all participants’ distributions).
- Participants requiring more than 10 minutes to wear the EEG device are excluded from EEG analysis.
- Additionally, participants whose EEG data were poorly measured or recorded are excluded from EEG analysis.

## Study Variables

(ICH E3; 9.5.1. ICH E9; 2.2.2)

|  | Day1  (pre-gaming) | Day1  (Post-gaming) | Day2  (24 hours Post-Gaming) | Day 4  (72 hours Post-Gaming) |
| --- | --- | --- | --- | --- |
| Feedback cycle times (Learning phase) | x | - | - | - |
| The frequency of Image repetition (Learning phase) | x | - | - | - |
| METEOR Score (Memory performance) | - | x | x | x |
| Gist Score (Memory performance) | - | x | x | x |
| EEG data (Neural activity) | - | x | - | - |
| Adverse event monitoring | x | x | x | x |

**Time-Window Definitions**

- Day 1 Pre-Gaming: observation collected before the gaming phase
- Day 1 Post-Gaming: Assessments conducted immediately after the gaming phase.
- Day 2 and Day 4 Follow-Ups: Assessments conducted within a 2-hour window of the scheduled time (e.g., between 22–26 hours for Day 2). Measurements collected outside this window will be flagged as deviations and handled during analysis.

**Rules for Measurements Outside Scheduled Times**

- For EEG and METEOR Score data collected outside the specified time-windows, observations will be excluded unless determined by the investigator to be reliable for analysis.
- Missing EEG epochs or METEOR scores due to technical issues will not be carried forward; instead, the remaining valid observations will be analyzed.

**Detailed Description of Variables**

1. Feedback cycle times (Learning phase) :

- Definition: The time participants take to process and respond to feedback during the learning phase
- Numeric Range: Recorded in seconds
- Exclusion Rule: Outliers defined as those outside the interquartile range (IQR) will be excluded from analysis.

1. The frequency of Image repetition (Learning phase):

- Definition: The number of times participants repeatedly viewed images during the learning phase.

1. METEOR Score (Memory Performance):

- Definition: Semantic similarity score reflecting accurate and context-rich recall of cue-target pairs.
- Numeric Range: continuous variables with no predefined range. Higher scores indicate greater semantic recall accuracy.
- Handling Missing Data: Missing scores will not be imputed.

1. Gist Score (Memory Performance):

- Definition: Percentage of core narrative elements (Gist items) accurately recollected from each scene, reflecting participants’ ability to recall essential story components.
- Numeric Range: expressed as percentages, with a theoretical range of 0% (no Gist items recollected) to 100% (all Gist items recollected).
- Handling Missing Data: Missing scores will not be imputed.

1. EEG Data (Neural Activity):

- Definition: Neural activity recorded during the gaming phase, segmented into 2-second epochs for analysis.
- Exclusion Rule: EEG data with significant artifacts (e.g., noise, poor electrode contact) will be excluded.

1. Adverse Event Monitoring:

- Definition: Documentation of any unexpected physiological or psychological effects during the study period.

# Sample Size

(ICH E3; 9.7.2. ICH E9; 3.5)

The sample size for the study was determined to be 150 participants based on a prior study [2], which measured EEG activity in adult males to investigate the therapeutic effects of NF training.

# General Considerations

## Timing of Analyses

The final analysis will be conducted after all participants have completed the study protocol, including the follow-up assessments on Day 4.

## Analysis Populations

(ICH E3; 9.7.1, 11.4.2.5. ICH E9; 5.2)

### Full Analysis Population

- All participants who completed the learning phase and provided at least one valid METEOR score or Gist score during the testing phases (Day 1, Day 2, Day 4).

### Per Protocol Population

- Successfully completed the gaming intervention and all post-gaming assessments without protocol deviations.

### Safety Population

- All participants who completed the learning phase participated in the gaming intervention (Day 1).
- Participants monitored for adverse events during or after the gaming phase (e.g., discomfort with EEG equipment).

## Covariates and Subgroups

(ICH E3; 9.7.1, 11.4.2.1. ICH E9; 5.7)

**1. Covariates**

The following covariates will be included in the analysis, particularly for difference in differences (DID) analysis conducted for secondary outcome:

- **Feedback Cycle Times:** The time taken during the learning phase will be analyzed to control for learning efficiency as a potential confounding factor.
- **Frequency of Image Repetition:** The number of times participants repeatedly viewed images during the learning phase analyzed as a measure of potential confounding factor on memory performance.

**2. Subgroups**

Subgroup analyses will be conducted to evaluate the interaction effects between the following variables:

- **Exposure Condition:**
  - Control, Conscious, Unconscious.
- **Emotional Valence:**
  - Positive, Negative.

**3. A Priori Hypotheses**

- **Hypothesis:** On Day 1, participants will exhibit lower METEOR and Gist scores for images cued through conscious or unconscious exposure compared to images in the control condition.

## Missing Data

(ICH E3; 9.7.1, 11.4.2.2. ICH E9;5.3. EMA Guideline on Missing Data in Confirmatory Clinical Trials)

Missing data will be quantified as a percentage for each variable, including METEOR scores, Gist scores, and EEG epochs.

- **Patterns of Missingness:** Missing data will be categorized as permanent, transient, or monotonous to identify potential causes.
- **Handling Missing Data:**
  - Missing METEOR and Gist scores will not be imputed; analyses will use available data (complete case analysis).
  - Missing EEG epochs due to artifacts will be excluded from analysis.

## Interim Analyses and Data Monitoring

(ICH E3; 9.7.1, 11.4.2.3. ICH E9; 4.1, FDA Feb 2010 “Guidance for Industry Adaptive Design Clinical Trials for Drugs and Biologics”)

No interim analyses or formal data monitoring procedures are planned for this study. The study will proceed as designed without modifications based on interim data reviews.

# Summary of Study Data

All continuous variables will be summarized using the following descriptive statistics: n (non-missing sample size), mean, standard deviation, median, maximum and minimum. The frequency and percentages (based on the non-missing sample size) of observed levels will be reported for all categorical measures. In general, all data will be listed, sorted by treatment and subject, and when appropriate by visit number within subject. All summary tables will be structured with a column for each treatment in the order (Control, Treatment) and will be annotated with the total population size relevant to that table/treatment, including any missing observations.

## Subject Disposition

**Study Stages and Participation:**

- Number of participants screened.
- Number of participants who completed the learning phase (Day 1).
- Number of participants who completed the gaming phase (Day 1).
- Number of participants who completed follow-up assessments on Day 2 (24 hours post-gaming) and Day 4 (72 hours post-gaming).
- Number of participants who dropped out, with reasons categorized as follows:
- Withdrawal of consent.
- Technical issues (e.g., EEG recording failure).
- Failure to adhere to protocol (e.g., excessive feedback cycle time deviations).

The summary statistics will be produced in accordance with section 9.

## Protocol Deviations

**1. Definition of Major Deviations**

Major deviations are defined as those that significantly affect the integrity of the data or the primary and secondary outcomes. Examples include:

- Participants whose feedback cycle times during the learning phase fall outside the interquartile range (IQR), indicating abnormal or inconsistent learning performance.
- Participants with significant EEG recording issues (e.g., excessive artifacts, technical failure) that result in unusable EEG data.
- Participants who failed to complete the gaming phase or any of the required assessments on Day 1, Day 2, or Day 4.

**2. Impact on Analysis Populations**

- **Full Analysis Population (FAP):**
  - Participants with minor deviations (e.g., small timing discrepancies) will remain included.
  - Major deviations will not exclude participants from the Full Analysis Population.
- **Per Protocol Population (PPP):**
  - Participants with major protocol deviations (e.g., unusable EEG data, extreme feedback cycle outliers) will be excluded.

The summary statistics will be produced in accordance with section 9.

## Demographic and Baseline Variables

As this study employs a within-subject design, detailed baseline comparisons between groups are not required. Only the following demographic variables will be recorded and summarized: Age, Gender, Corrected Visual Acuity (Binocular average)

The summary statistics for these variables will be produced in accordance with Section 9 of this SAP.

## Concurrent Illnesses and Medical Conditions

There are no concurrent illnesses or medical conditions recorded for this study, as they are not relevant to the study design or analysis.

## Prior and Concurrent Medications

No prior or concurrent medications were recorded in this study, as they are not relevant to the study design or outcomes.

## Treatment Compliance

In this study, treatment compliance will be evaluated to determine how well participants adhered to the experimental procedures. Since the study relies on experimental tools such as the gaming intervention and EEG recordings, compliance will be assessed based on two main criteria. First, usage records will be used to track the time participants spent engaging with the gaming intervention during the experimental phase. Second, the quality of EEG data will be considered, specifically focusing on the number of valid EEG epochs collected during the gaming session. These measures reflect the participants’ adherence to the study protocol and the reliability of the data collected.

# Efficacy Analyses

The efficacy variables in this study will be summarized to evaluate the effects of exposure condition (Control, Conscious, Unconscious), emotional valence (Positive, Negative), and test timepoint (Day 1, Day 2, Day 4).

For each combination of exposure condition, valence, and timepoint, the continuous efficacy variables, METEOR scores, will be summarized using descriptive statistics. Specifically, the mean and standard deviation (SD) will be calculated to provide an overview of performance across the different treatment groups. To formally compare METEOR scores between groups, paired t-tests will be conducted at a 5% significance level.

## Primary Efficacy Analysis

The primary efficacy analysis will assess the effect of cue exposure conditions (Conscious, Unconscious, Control) on short-term memory suppression, measured by METEOR scores. This analysis will be conducted across the total image group (include all images) as well as within the negative and positive image subgroups focusing on data collected at day 1.

For the total image group, the analysis will test whether METEOR scores for images in the Conscious and Unconscious exposure conditions are lower than those in the Control condition. Similarly, separate analyses will be performed for the negative image group and positive image group to determine if the same pattern holds within these subgroups. The following hypotheses will be tested using one-tailed paired t-tests at a 5% significance level:

- Total Image Group: METEOR scores for the Conscious and Unconscious conditions will be lower than for the Control condition.
- Negative Image Group: METEOR scores for the Conscious and Unconscious conditions will be lower than for the Control condition.
- Positive Image Group: METEOR scores for the Conscious and Unconscious conditions will be lower than for the Control condition.

To support these comparisons, descriptive statistics (mean, standard deviation, t statistics, p-values) will be calculated for each combination of exposure condition (Conscious, Unconscious, Control) and valence (Positive, Negative). These statistics will provide a clear summary of the METEOR scores across conditions and groups. The results of the primary efficacy analysis, including both descriptive summaries and statistical tests, will be presented in accordance with Section 8 of this SAP.

## Secondary Efficacy Analyses

The secondary efficacy analyses will extend the evaluation of short-term memory suppression and further explore long-term effects, subgroup differences, and comparisons across emotional valence.

1. Long-Term Memory Suppression Analysis

The primary endpoint analysis conducted on Day 1 will be repeated on Day 2 (24 hours post-gaming) and Day 4 (72 hours post-gaming). This will determine whether the suppression effects observed (Conscious < Control, Unconscious < Control) persist over time, indicating long-term memory suppression.

1. Time-Based Changes in Memory Performance

To assess how memory performance changes over time for each subgroup (control, treatment, conscious, unconscious), a Difference-in-Difference (DID) analysis will be conducted. This analysis will be adjusted for the following covariates:

- Total duration of the self-feedback process during the Learning phase.
- Repetition frequency of each image during the Learning phase.

1. Emotional Valence Comparisons

To examine the differences between negative and positive emotional valence at each timepoint (Day 1, Day 2, Day 4), paired two-tailed t-tests will be performed. The comparisons will include:

- Negative Treatment vs Positive Treatment
- Negative Conscious vs Positive Conscious
- Negative Unconscious vs Positive Unconscious
- Negative Conscious vs Positive Unconscious
- Negative Unconscious vs Positive Conscious

These analyses will evaluate whether emotional valence influences memory suppression and whether differences between groups change over time.

To support these comparisons, descriptive statistics (mean, standard deviation, t statistics, coefficient estimates, p-values) will be calculated. The summary statistics and results for the secondary efficacy analyses will be produced in accordance with Section 8 of this SAP.

## Exploratory Efficacy Analyses

The exploratory efficacy analysis will evaluate differences in neural activity (EEG data) across two comparisons:

1. Cue Exposure Condition: Neural activity during the Conscious phase will be compared to the Unconscious phase, regardless of emotional valence.
2. Emotional Valence: Neural activity for Negative images will be compared to Positive images, regardless of cue exposure condition.

To minimize individual variability, EEG segments will be paired within participants:

- For cue exposure comparisons, the i-th EEG segment from the Conscious phase will be paired with the corresponding i-th segment from the Unconscious phase.
- For emotional valence comparisons, EEG segments from Negative and Positive image groups will be paired for the same participant.

Statistical significance will be assessed using two-sided Wilcoxon signed-rank tests for each set of paired comparisons. To support these comparisons, descriptive statistics (median value of power spectral density for each channel and frequency band, W statistics, p-values) will be calculated. The summary statistics and results will be reported in accordance with Section 9 of this SAP.

# Safety Analyses

The safety analyses for this study will focus on evaluating any potential discomfort or adverse events related to EEG equipment use and the gaming intervention.

## Extent of Exposure

Exposure will be assessed by summarizing the number of completed game sessions (out of 18 total) and the total EEG recording duration per participant.

## Adverse Events

No serious adverse events (AEs) are anticipated due to the non-invasive nature of the study. However, any minor discomfort, such as physical irritation caused by EEG equipment or self-reported stress during the gaming phase, will be documented. Each participant will only be counted once for any reported AE type, and the denominator will be the total number of participants.

# Figures

**Primary and Secondary Endpoints:**

- Line graphs and boxplots will display METEOR scores across exposure conditions (Conscious, Unconscious, Control), emotional valence (positive, negative) and timepoints (Day 1, Day 2, Day 4).

**Exploratory Endpoints**

- Topographical Brain Plots: Visualizations of power spectrum density will be created to display the median differences in each EEG channel across the following frequency bands: alpha, beta, theta, delta, and gamma.
  - Comparisons will include:
- Conscious vs Unconscious
- Negative vs Positive

# Reporting Conventions

P-values ≥0.001 will be reported to 3 decimal places; p-values less than 0.001 will be reported as “<0.001.” Estimates, means, standard deviations, and other statistics will be reported to three decimal places and will match the precision of the original data where applicable.

# Technical Details

All analyses will be conducted using **Python (Version 3.12.0)** and **R (Version 4.4.0)**. Data, analysis code, and output documents will be stored in the following url: *“https://osf.io/qbdu7/files/osfstorage”*

# References

1. Brewin CR, Gregory JD, Lipton M, Burgess N. Intrusive images in psychological disorders: characteristics, neural mechanisms, and treatment implications. *Psychological Review.* 2010;117:210.
2. Keynan JN, Cohen A, Jackont G, et al. Electrical fingerprint of the amygdala guides neurofeedback training for stress resilience. *Nature Human Behavior.* 2019;3**:**63-73.
3. Ruxton GD, Neuhäuser M. When should we use one‑tailed hypothesis testing? *Methods in Ecology and Evolution.* 2010;1:114-117.

# Listing of Tables, Listings and Figures

| **Table Title** | **Number** | **Population** | **Endpoint** | **Time Points or how to conglomerate** | **Covariates or Subgroups** | **Summary Statistics** | **Formal Analysis** | **Foot Notes** | |  |
| --- | --- | --- | --- | --- | --- | --- | --- | --- | --- | --- |
| Disposition | 1 | NA | Disposition | enroll-day4 | Treatment | count | NA |  | |  |
| Summary of Demographic Variable | 2 | Full Analysis | Age | Baseline | Treatment | n, mean, SD | NA |  | |  |
|  |  |  | Gender | Baseline | Treatment | p% (x/n) | NA |  | |  |
|  |  |  | Corrected Vision | Baseline | Treatment | n, mean, SD | NA |  | |  |
| Summary of scores (METEOR, Gist) | 3.1 | Full Analysis | METEOR scores | Day1 | Full(Treatment, Conscious, Unconscious), Negative(Treatment, Conscious, Unconscious), Positive(Treatment, Conscious, Unconscious) | n, mean, SD | NA |  | |  |
|  |  | Full Analysis | Gist scores | Day1 | As above | n, mean, SD | NA |  | |  |
|  | 3.2 | Full Analysis | METEOR scores | Day2 | As above | n, mean, SD | NA |  | |  |
|  |  | Full Analysis | Gist scores | Day2 | As above | n, mean, SD | NA |  | |  |
|  | 3.3 | Full Analysis | METEOR scores | Day4 | As above | n, mean, SD | NA |  | |  |
|  |  | Full Analysis | Gist scores | Day4 | As above | n, mean, SD | NA |  | |  |
| ShIF in short-term (paired t test) | 4.1 | Full Analysis | METEOR scores | Day1 | As above | n, mean, t statistics, p | NA |  | |  |
|  |  | Full Analysis | Gist scores | Day1 | As above | n, mean, t statistics, p | NA | | |  |
| ShIF in long-term (paired t test) | 4.2 | Full Analysis | METEOR scores | Day2-Day4 | As above | n, mean, t statistics, p | NA | | |  |
|  |  | Full Analysis | Gist scores | Day2-Day4 | As above | n, mean, t statistics, p | NA | | |  |
| DID analysis | 5.1 | Full Analysis | METEOR scores | Day1-Day2, Day2-Day4 | As above | Estimate, standard error, p | NA | |  |  |
|  | 5.2 | Full Analysis | Gist scores | Day1-Day2, Day2-Day4 | As above | Estimate, standard error, p | NA | |  |  |
| The difference in the Power Spectrum Density between “conscious and unconscious” | 6.1 | Full Analysis | PSD values | Day1 | Comparison (Conscious - Unconscious) | PSD, W statistics, p | NA | |  |  |
| The difference in the Power Spectrum Density between “positive and negative” | 6.1 | Full Analysis | PSD values | Day1 | Comparison (Positive - Negative) | PSD, W statistics, p | NA | |  |  |

| **Title** | **Number** | **Population** | **Type of graph** | **Horizontal Variables** | **Vertical Variables** | **Groupings** | **Statistics** | **Facets** |
| --- | --- | --- | --- | --- | --- | --- | --- | --- |
| Boxplot of short-term ShIF | 6.1 | Full Analysis | Boxplot | Treatment | METEOR score | Exposure condition, Emotional valence | Mean, Median, IQR, minimum, maximum | NA |
| Long-term analysis across exposure conditions of image | 6.2 | Full Analysis | Line graph | time | METEOR score | Exposure condition | Mean, DID estimates | NA |
| Long-term analysis across emotional valence of image | 6.3 | Full Analysis | Line graph | time | METEOR score | Exposure condition, Emotional valence | Mean | NA |
| The median difference in EEG power spectrum density | 6.4 | Full Analysis | Topographical brain plot | - | -- | Comparison pairs (conscious-unconscious, positive-negative) | W statistics, power spectrum density | NA |

Having reached the end you should edit the headers and footers to add in the correct study title, change the version number, the date you finalized the current version (do not use the automatic “today’s date” as this will change each time you open the document). Also check the accuracy of the table at the top of the document. Update the table of contents. Leave in this reminder paragraph until the final version is confirmed.
